# The Impact of Exercise Prescription Variables on Intervention Outcomes in Musculoskeletal Pain: An Umbrella Review of Systematic Reviews

**DOI:** 10.1007/s40279-023-01966-2

**Published:** 2023-12-14

**Authors:** Nitin Kumar Arora, Lars Donath, Patrick J. Owen, Clint T. Miller, Tobias Saueressig, Felicitas Winter, Marina Hambloch, Christopher Neason, Vera Karner, Daniel L. Belavy

**Affiliations:** 1 Division of Physiotherapy, Department of Applied Health Sciences, Hochschule für Gesundheit (University of Applied Sciences), Gesundheitscampus 6-8, 44801 Bochum, Germany; 2 Department of Intervention Research in Exercise Training, German Sport University Cologne, Cologne, Germany; 3 Institute for Physical Activity and Nutrition (IPAN), School of Exercise and Nutrition Sciences, Deakin University, Geelong, VIC Australia; 4 Science and Research, Physio Meets Science GmbH, Leimen, Baden-Württemberg Germany

## Abstract

**Background:**

Musculoskeletal pain conditions are the largest contributors to disability and healthcare burden globally. Exercise interventions improve physical function and quality of life in individuals with musculoskeletal pain, yet optimal exercise prescription variables (e.g. duration, frequency, intensity) are unclear.

**Objective:**

We aimed to examine evidence gaps, methodological quality and exercise prescription recommendations in systematic reviews of exercise for musculoskeletal pain.

**Methods:**

In our prospectively registered umbrella review, PubMed, SPORTDiscus, Cochrane Database of Systematic Reviews, EMBASE, and CINAHL were searched from inception to 14 February 2023. Backward citation tracking was performed. We included peer-reviewed, English language, systematic reviews and meta-analyses of randomized controlled trials (RCTs) and controlled clinical trials (CCTs) that compared exercise with conservative treatment, placebo or other exercise interventions in adults with musculoskeletal pain. Data were extracted from the following groups of reviews based on their reporting of exercise prescription data and analysis of the relationship between prescription variables and outcomes: (1) those that did not report any exercise prescription data, (2) those that reported exercise prescription data but did not perform a quantitative analysis and (3) those that performed a quantitative analysis of the relationship between exercise prescription variables and outcomes. Outcome measures were physical function, pain, mental health, adverse effects and adherence to treatment. AMSTAR-2 (A MeaSurement Tool to Assess systematic Reviews) was used to assess methodological quality.

**Results:**

From 6757 records, 274 systematic reviews were included. 6.6% of reviews did not report any exercise prescription data, and only 10.9% quantitatively analyzed the relationship between prescription variables and the outcome(s). The overall methodological quality was critically low in 85% of reviews.

**Conclusion:**

High methodological quality evidence is lacking for optimal exercise training prescription variables in individuals with musculoskeletal pain. To better inform practice and evidence gaps, future systematic reviews should (1) identify optimum exercise prescription variables, for example, via dose–response (network) meta-analysis, (2) perform high-quality reviews per AMSTAR-2 criteria and (3) include outcomes of mental health, adverse events and exercise adherence.

**PROSPERO registration number:**

CRD42021287440 (https://www.crd.york.ac.uk/prospero/display_record.php?ID=CRD42021287440).

**Supplementary Information:**

The online version contains supplementary material available at 10.1007/s40279-023-01966-2.

## Key Points

**Table:** 

The majority of the included systematic reviews assessing the impact of exercise prescription variables on intervention outcomes in musculoskeletal pain had critically low methodological quality.
Knee pain and low back pain were the most commonly reviewed musculoskeletal pain conditions.
Most reviews reported only the frequency and duration of exercise prescription, with around 11% of the available evidence analyzing exercise prescription for physical function, pain and mental health.
More frequent exercise training leads to greater improvements in musculoskeletal pain and physical function.

## Introduction

Musculoskeletal pain contributes to a considerable burden on health care with an average prevalence of 40%, translating to approximately 1.7 billion individuals with pain globally [1]. Musculoskeletal disorders tend to progress with advancing age [2]. Based on the duration of symptoms, musculoskeletal pain can be defined as acute, sub-acute or chronic presentation, with chronic musculoskeletal pain being most common (> 40%) [3, 4]. Musculoskeletal pain is referred to as *“pain as a result of injuries or disorders affecting the bone, joints, muscles, or other associated soft tissues”* [5]. Among the various pain conditions, neck and low back pain are the leading contributors to years lived with disability, work absenteeism, and increased health care expenses [6]. A vast majority (70–80%) of individuals in developed countries experience low back pain at some point in their lives [7]. The majority of developed and developing nations experienced a rise in socioeconomic burden, with health care costs for musculoskeletal pain exceeding €250 billion in Europe [8], approximately US$180 billion in the United States [9] and around £10 billion for back pain alone in the United Kingdom [10]. Hence, identifying an effective treatment for the management of musculoskeletal pain is a vital step in reducing the healthcare-related costs and improving the quality of life of individuals with musculoskeletal pain globally.

Several surgical and invasive management strategies have been proposed in the past but research is increasingly focusing on approaches like exercise interventions that are less expensive and have fewer adverse events but still provide similar outcomes [11]. Exercise training improves pain intensity, physical function and quality of life in individuals with chronic pain [12, 13]. Exercise training is defined as “*a specific type of physical activity that is planned, structured, repetitive and purposeful with a final or intermediate goal of improving or maintaining physical fitness*” [14].

Despite the presence of various exercise types for managing musculoskeletal pain, there is a paucity of evidence within clinical practice guidelines to guide the optimal dose of exercise training [15]. Moreover, the effects of exercise interventions are likely to vary in different meta-analyses of musculoskeletal pain as the exercise dose prescription (frequency, intensity, duration, and volume of training) differs widely in individual trials [16]. Previous research suggests that individualization of exercise dose requires an efficient alteration of the exercise prescription variables (dose) for an improvement in musculoskeletal pain (response) and it could also contribute to the differences in effect observed because of inter-individual differences [17, 18]. Over-prescription can contribute to an increased chance of injury while an under-prescription of exercise dose might fail to provide adequate relief of symptoms. In recent years, pain research has focused on quantifying an ‘optimal’ dose–response relationship for individualization of exercise training to reduce pain and disability in musculoskeletal pain [19, 20]. Previous studies have shown the positive impact of exercise prescription in reducing the risk of various health conditions, including coronary heart disease, heart failure, dementia, etc. [21–23]. With the increasing number of systematic reviews of exercise for musculoskeletal pain, it is important to identify the quality and quantity of reviews that compare exercise interventions with other conservative treatments, exercise or placebo, and identify evidence gaps and exercise prescription recommendations for different pain conditions.

The aim of this umbrella review was to systematically synthesize the current systematic review evidence on exercise interventions for musculoskeletal pain and evaluate methodological quality, reporting of exercise prescription variables (e.g. frequency, intensity, duration, volume) and analysis of exercise prescription variables in relation to the outcome(s) assessed.

## Methods

We followed the current Cochrane guidance on ‘Overview of reviews’ for conducting this umbrella review and reported the results in accordance with the guidelines of the Preferred Reporting Items for Overviews of Reviews (PRIOR) overview for reporting healthcare interventions (Supplementary File 1, see electronic supplementary material [ESM]) [24, 25]. This umbrella review was prospectively registered on PROSPERO (CRD42021287440) before initiating the screening process.

### Data Sources and Search Strategy

**Fig. 1:**
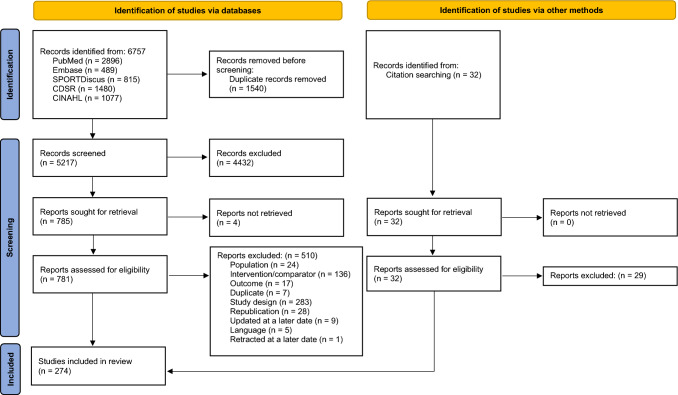
Preferred Reporting Items for Systematic review and Meta-Analysis flow diagram of studies [85]

Five electronic databases (PubMed, SPORTDiscus, Cochrane Database of Systematic Reviews (CDSR), EMBASE and CINAHL) were searched from inception to 1 October 2021 and updated on 14 February 2023 (Fig. 1). A combination of MeSH terms and keywords for exercise, pain and review was used with one or more Boolean operators (AND, OR, NOT). The full search strategy is available in Supplementary File 2 (see ESM). We also conducted backward citation tracking of the included systematic reviews to identify further relevant records.

### Eligibility Criteria

We included systematic reviews that fulfilled the following criteria:

*Population* Reviews should have included adults (> 18 years) with acute, sub-acute or chronic musculoskeletal pain [5, 26]. We excluded reviews of non-musculoskeletal conditions (fracture, metastasis, visceral pain, iatrogenic pain, pregnancy, spinal cord injury, intermittent claudication, etc.). A complete list of exclusion criteria is described in Supplementary File 3 (see ESM).

*Intervention and Comparator* Exercise interventions compared with active control exercise (head-to-head comparisons), passive control (non-pharmacological/non-surgical conservative treatment, sham/placebo, usual care) or true control (no treatment, waitlist control).

*Outcome* Physical function, pain, psychological function, adverse events or adherence rates.

*Study Design* English language systematic review and meta-analysis of randomized controlled trials (RCTs) and controlled clinical trials (CCTs) published in a peer-reviewed journal without any restriction on the year of publication. The systematic review should have defined a strategy for searching, appraising and synthesizing data from studies [27].

### Study Selection

We uploaded all search results to Covidence [28] for title abstract and full-text screening. Duplicates were automatically removed by Covidence [28] before starting the title and abstract screening. Seven reviewers (NKA, DLB, PJO, CTM, VK, CN, FBJ) performed a pilot screening on 100 records to test and refine the eligibility criteria followed by the title-abstract and full-text assessment of the search results by four reviewers (NKA, MH, VK, FBJ). One reviewer (VK) performed random cross checking of 200 titles from the list of excluded studies as a quality control measure in the process of screening. Disagreements in the selection process were resolved by discussion. Another reviewer (PJO) was contacted when disagreement persisted following discussion.

### Data Extraction

Two reviewers (NKA, VK) performed duplicate independent pilot data extraction of 10 reviews to test the data extraction (sample extraction template can be accessed via this https://osf.io/hgu9p/?view_only=c6958c60516e49fe81bad83f1f4659f0) and then four (NKA, CN, FW, VK) reviewers extracted data independently in duplicate from the included systematic reviews and meta-analyses. No additional data were extracted from the primary studies. Random cross-checking of the extracted data was conducted by one reviewer (VK) to ensure optimal quality of data extraction. One reviewer (NKA) independently performed the process of overlap analysis. Conflicts in data extraction were assessed by a custom-written code in the R statistical environment (version 4.2.1, www.r-project.org; custom-made code for sheet comparison available https://osf.io/hgu9p/?view_only=c6958c60516e49fe81bad83f1f4659f0) [29]. Conflicts were resolved by discussion. An adjudicator (PJO) was contacted when disagreement persisted following discussion.

#### Data Items

We extracted data for review inclusion characteristics (number of participants, number of included primary studies), search strategy of the review (name and number of databases searched, start and end date of searches, if available), population (age, health conditions, number of females, pain chronicity), intervention/comparator (types of exercise and comparators analyzed), quantitatively analyzed outcomes (physical function, pain, mental health, adverse events, adherence rate), exercise prescription reporting (includes information about whether the review extracted details about exercise prescription variables from individual studies; any analysis carried out to compare exercise prescription dose in the reviews). For systematic reviews and meta-analyses of exercise prescription variables, we extracted the following information: effect size, confidence interval, number of studies and participants included in the meta-analysis, type of exercise, details about the exercise prescription variables (duration, frequency, intensity or volume of exercise training), *p*-value for the summary effect, I^2^ value, R^2^ value, *p*-value for the chi-square test of the null hypothesis stating there is no heterogeneity, type of regression and follow-up time. In case of multiple updates of systematic reviews, we only included the most recent and detailed review [24]. Pending extraction item, we extracted data in the form of raw numbers, percentages or dichotomous responses (yes/no). We classified the information extracted from the reviews into various subgroups of pain diagnosis, viz., lower back pain, knee pain, shoulder pain, chronic widespread pain, rheumatoid arthritis, neck pain, ankle and foot pain, elbow pain, hip pain, hand pain and combination of chronic musculoskeletal disorders. For each review describing dose prescription, we extracted the identifying information for primary studies used in the included reviews to analyze the degree of overlap (available https://osf.io/hgu9p/?view_only=c6958c60516e49fe81bad83f1f4659f0). We used a citation matrix to visualize the degree of overlap of primary studies across the included systematic reviews. The citation matrix was created with the rows containing all the primary studies and columns containing names of all the reviews that analyzed exercise prescription. Each primary study was marked with a rating ‘1’ at the point of first appearance (unique publications) and all the duplicate studies were removed.

### Methodological Quality

The methodological quality of the selected reviews was assessed independently in duplicate by two of four assessors (NKA, FW, CN, VK) using the AMSTAR-2 (A MeaSurement Tool to Assess systematic Reviews) [30]. Disagreements were resolved by discussion. AMSTAR-2 is a widely used instrument for critical appraisal of reviews that include randomized and non-randomized studies. It consists of 16 critical and non-critical questions that can have responses like ‘yes’, ‘no’ or ‘partial yes’.

### Degree of Primary Study Overlap

We calculated the covered area (CA; formula: *N/rc*) and corrected covered area (CCA; formula: (*N* – *r*)/((r*c) – *r*)) for quantifying the overlap as slight (CCA 0–5%), moderate (CCA 6–10%), high (CCA 11–15%) and very high (CCA > 15%) [31, 32], where ‘N’ is the total number of publications (obtained by adding all the tick boxes rated as ‘1’), ‘*r*’ indicates the total number of rows (i.e., the unique, non-duplicate primary studies) and ‘c’ indicates the total number of columns (i.e., the total number of included reviews). Complete data for the primary studies and overlap analysis can be found in an online repository (https://osf.io/hgu9p/?view_only=c6958c60516e49fe81bad83f1f4659f0).

### Data Synthesis of Included Reviews

We summarized the characteristics of the included reviews in tabular form. As reviews utilized different types of meta-analyses (e.g., meta-regression, subgroup analysis, sensitivity analysis), we used a narrative synthesis rather than performing a meta-analysis of the results of multiple reviews. We synthesized the data from the systematic reviews/meta-analyses that used exercise interventions into three groups: (1) those that did not extract any exercise prescription data, (2) those that extracted exercise prescription data from the included trials but did not perform a quantitative analysis of the relationship between prescription variables and the outcome(s), and (3) those that performed a quantitative analysis of the relationship between prescription variables and the outcome(s). We then summarized data in terms of means and percentages. Where quantitative sub-group analysis results for exercise dose variables were available in the included reviews, these results were visualized and forest plots were generated for each exercise prescription variable and pain condition using R (v4.2.1) (https://www.rstudio.com/) [29], ggplot 2 [33], and meta (v5.5.0) [34] packages. Statistical codes for plotting the forest plots can be found in an online repository (https://osf.io/hgu9p/?view_only=c6958c60516e49fe81bad83f1f4659f0). Where meta-regression was performed in the included reviews, results from meta-regression were summarized in a table. A narrative summary of the findings on exercise prescription was performed.

Data for AMSTAR-2 ratings were summarized as an overall rating (critically low, low, moderate, and high) based on critical (question number 2, 4, 7, 9, 11, 13, 15) and non-critical weaknesses (question number 1, 3, 5, 6, 8, 10, 12, 14, 16) in the selected reviews [30]. We also calculated the percentage of reviews rating ‘yes’ on a particular question in order to identify the degree of adherence to individual items in the AMSTAR-2 tool.

For the overlap analysis, we calculated the corrected covered area and graphically presented the overall corrected covered area for the included reviews using ‘ccaR’ package [35]. We also conducted a sequential pairwise comparison of different reviews for each pain condition to assess the degree of overlap between reviews.

## Results

### Study Selection

The databases yielded a total of 6757 records (1540 duplicates removed via Covidence). Out of the remaining 5217 records, we excluded 4432 results at the title/abstract screening stage. Four full texts could not be retrieved and hence we assessed the full texts of the remaining 781 reviews and finally included 274 systematic reviews and meta-analyses for this umbrella review. Three of these included records were identified via backward citation tracking. Supplementary File 4 provides a detailed list of excluded studies along with the reasons for exclusion (see ESM).

### Study Characteristics

The characteristics of the included reviews (*N* = 274) are summarized in Supplementary File 5 (complete reference list available in Supplementary File 6, see ESM). All the included reviews used more than two databases to search for primary studies, with Cochrane CENTRAL (*n* = 215), Medline (*n* = 172), Embase (*n* = 189), Cumulative Index to Nursing and Allied Health Literature (CINAHL) (*n* = 153) and Physiotherapy Evidence Database (PEDro) (*n* = 138) being the most commonly used databases. Of the included reviews, 191 (69.71%) focused on chronic pain, 82 (29.93%) on a combination of pain duration levels and one (0.4%) on sub-acute pain in adults (> 18 years). The majority of included reviews used the Cochrane risk-of-bias tool (59.9%, *n* = 164) followed by PEDro (27.4%, *n* = 75) and Jadad (3.3%, *n* = 9) to assess the methodological quality of the included studies.

### Methodological Quality of Included Reviews

**Fig. 2:**
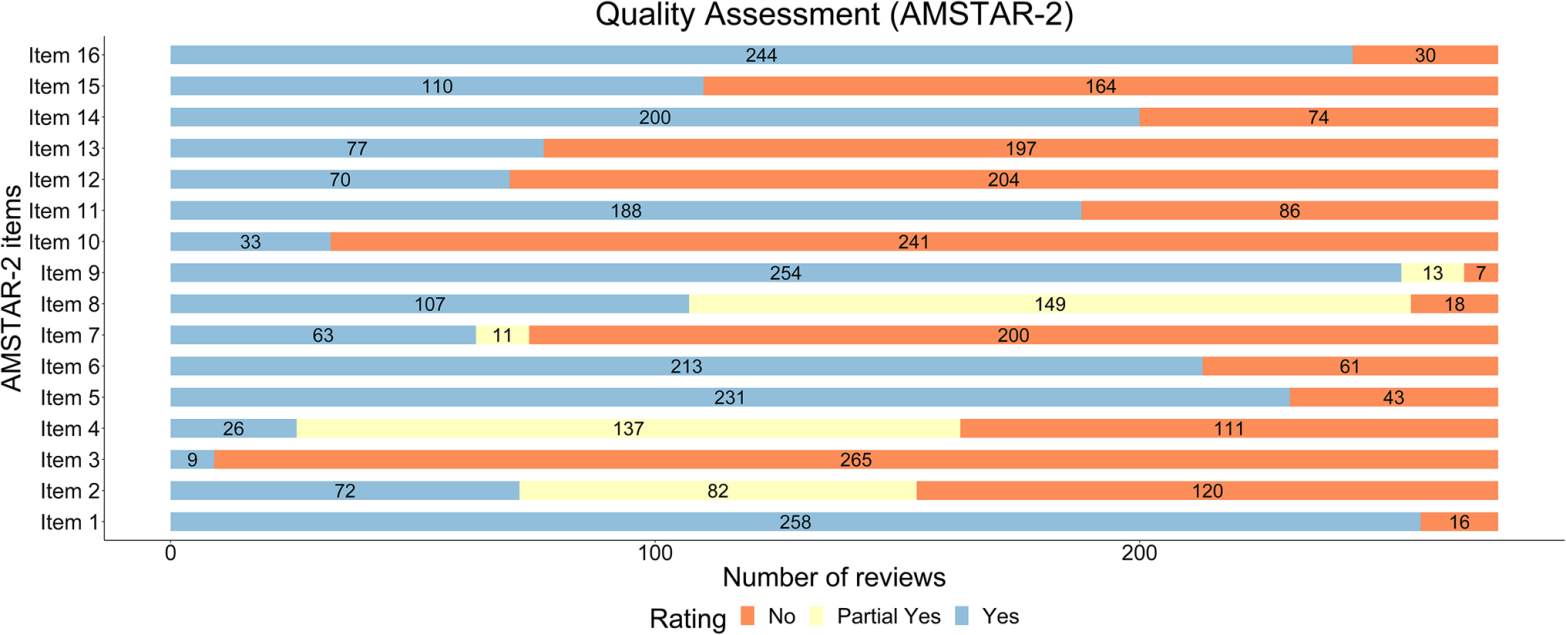
Number of reviews adhering to the signaling questions of the AMSTAR-2 tool (A MeaSurement Tool to Assess systematic Reviews-2). [30] The number of ‘No’ ratings on critical (question number 2, 4, 7, 9, 11, 13, 15) and non-critical (question number 1, 3, 5, 6, 8, 10, 12, 14, 16) items was used in allocating an overall rating of critically low, low, moderate or high

Out of the 274 reviews included in this umbrella review, most (*n* = 233) were rated as ‘critically low’ quality per AMSTAR-2. Only seven reviews were rated ‘high’ quality [36–42], nine were rated as having a ‘moderate’ quality and 25 were rated ‘low’ quality. The reviews did not adequately report on the following quality items: item 3 *(rationale for selection of RCTs*) (96.7%, *n* = 265), item 10 *(information about funding sources of included primary studies)* (87.9%, *n* = 241), item 12 *(assessed the impact of risk of bias in included studies on meta-analysis results)* (74.5%, *n* = 204), item 7 *(provided a list of excluded studies with rationale for exclusion)* (72.9%, *n* = 200), item 13 *(discussed the impact of risk of bias in individual studies on review results)* (71.9%, *n* = 197) and item 15 *(assessment for publication bias)* (59.9%, *n* = 164). Figure 2 presents the individual items of AMSTAR-2 and their relative contribution to the overall score (complete AMSTAR-2 rating for all the included reviews is available in Supplementary File 7, see ESM).

### Results of Individual Reviews: Narrative Summary of All Reviews

The included reviews examined low back pain (*n* = 72), knee pain (*n* = 65), shoulder pain (*n* = 17), neck pain (*n* = 23), rheumatoid arthritis (*n* = 8), hip pain (*n* = 7), ankle and foot pain (*n* = 6), chronic widespread pain (*n* = 23), hand pain (*n* = 3) and elbow pain (*n* = 3). All the included reviews used one of the following types of exercise training: aerobic (*n* = 10), mind–body (*n* = 40), resistance (*n* = 44), stabilization (*n* = 23), stretching (*n* = 2), other and water-based (*n* = 21) and a combination of two or more exercise types (*n* = 134). The exercise interventions were compared with conservative interventions (*n* = 25), no or minimal intervention (placebo, sham, usual care, no treatment or waitlist control) (*n* = 25), other exercise interventions (*n* = 34), a combination of exercise and conservative comparators (*n* = 66), a combination of conservative and minimal intervention (*n* = 48), a combination of exercise and minimal intervention (*n* = 22) or a combination of exercise, conservative and minimal intervention (*n* = 54).

Pain intensity (*n* = 267, 97.4%) and physical function (*n* = 230, 83.9%) were the most commonly assessed outcomes, followed by mental health (*n* = 40, 14.6%), adherence rate (*n* = 12, 6.9%) and adverse events (*n* = 12, 6.9%). Amongst exercise prescription variables, duration (*n* = 256, 93.4%) was the most commonly reported, followed by frequency (*n* = 225, 82.1%), intensity (*n* = 64, 23.4%) and volume (*n* = 58, 21.2%). A narrative summary of the included studies per diagnosis is available in Supplementary File 8 (see ESM).

### Findings from Reviews Assessing Exercise Prescription

Of the 274 studies, 93.4% (*n* = 256) reported data on the prescription variables, but only 10.9% (*n* = 30; 17 for physical function and pain intensity, 20 for physical function, 25 for pain intensity and 2 for mental health) quantitatively analyzed the relationship between prescription variables and the outcome(s) [16, 19, 20, 43–69]. A narrative summary of exercise prescription from the included studies per diagnosis is available in Supplementary File 9 (see ESM).

**Fig. 3:**
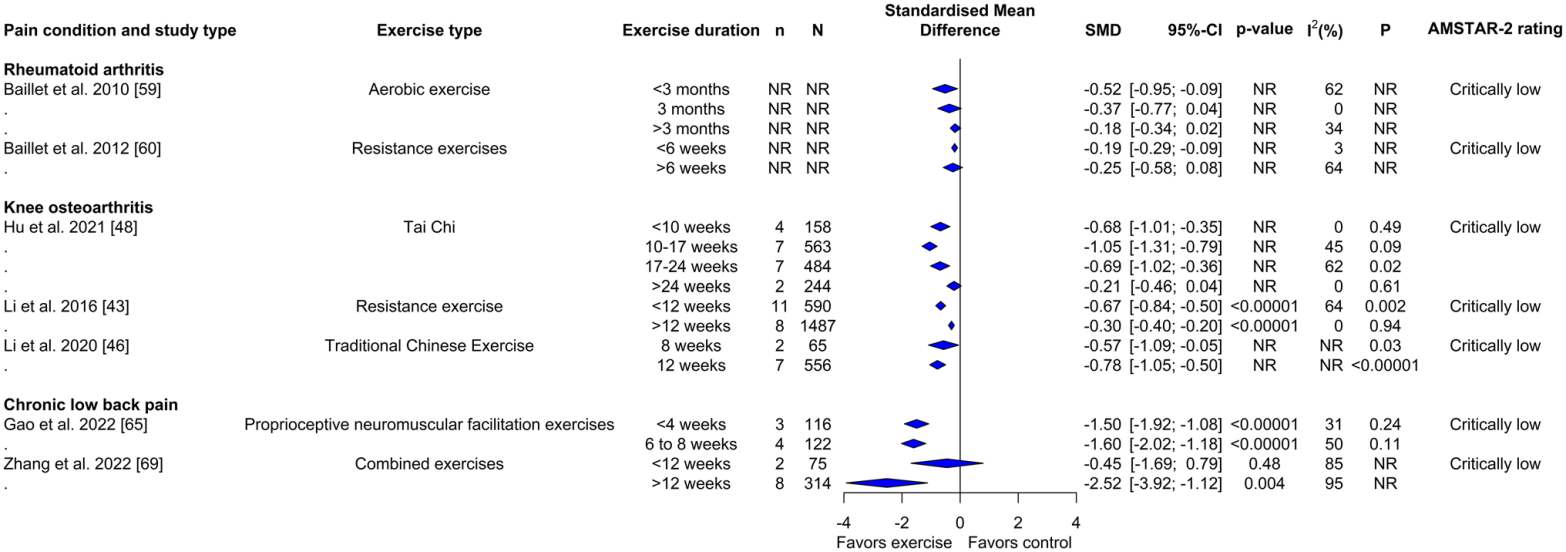
Forest plot demonstrating the effects of exercise duration in reviews of different musculoskeletal pain conditions for change in physical function outcome. Standardized mean differences (SMDs) and other data are as reported in the meta-analyses of individual studies. The results are reverse scaled and negative values indicate an improvement with exercise interventions. See supplementary file 10 in the electronic supplementary material for results from meta-regressions. *95%-CI* 95% confidence interval, *n* Number of studies in meta-analysis, *N* number of participants in meta-analysis, *NR* not reported, *P p*-value for the chi-square test of the null hypothesis stating that there is no heterogeneity

**Fig. 4:**
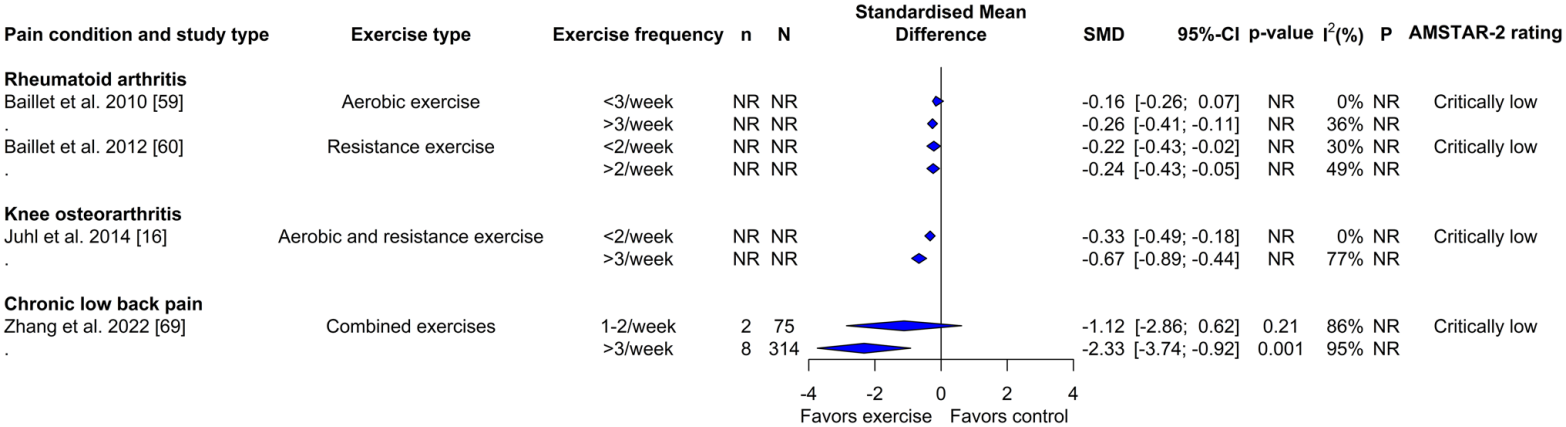
Forest plot demonstrating the effects of exercise frequency in reviews of different musculoskeletal pain conditions for change in physical function outcome. Standardized mean differences (SMDs) and other data are as reported in the meta-analyses of individual studies. The results are reverse scaled and negative values indicate an improvement with exercise interventions. See supplementary file 10 in the electronic supplementary material for results from meta-regressions. *95%-CI* 95% confidence interval, *n* Number of studies in meta-analysis, *N* number of participants in meta-analysis, *NR* not reported, *P p*-value for the chi-square test of the null hypothesis stating that there is no heterogeneity

**Fig. 5:**
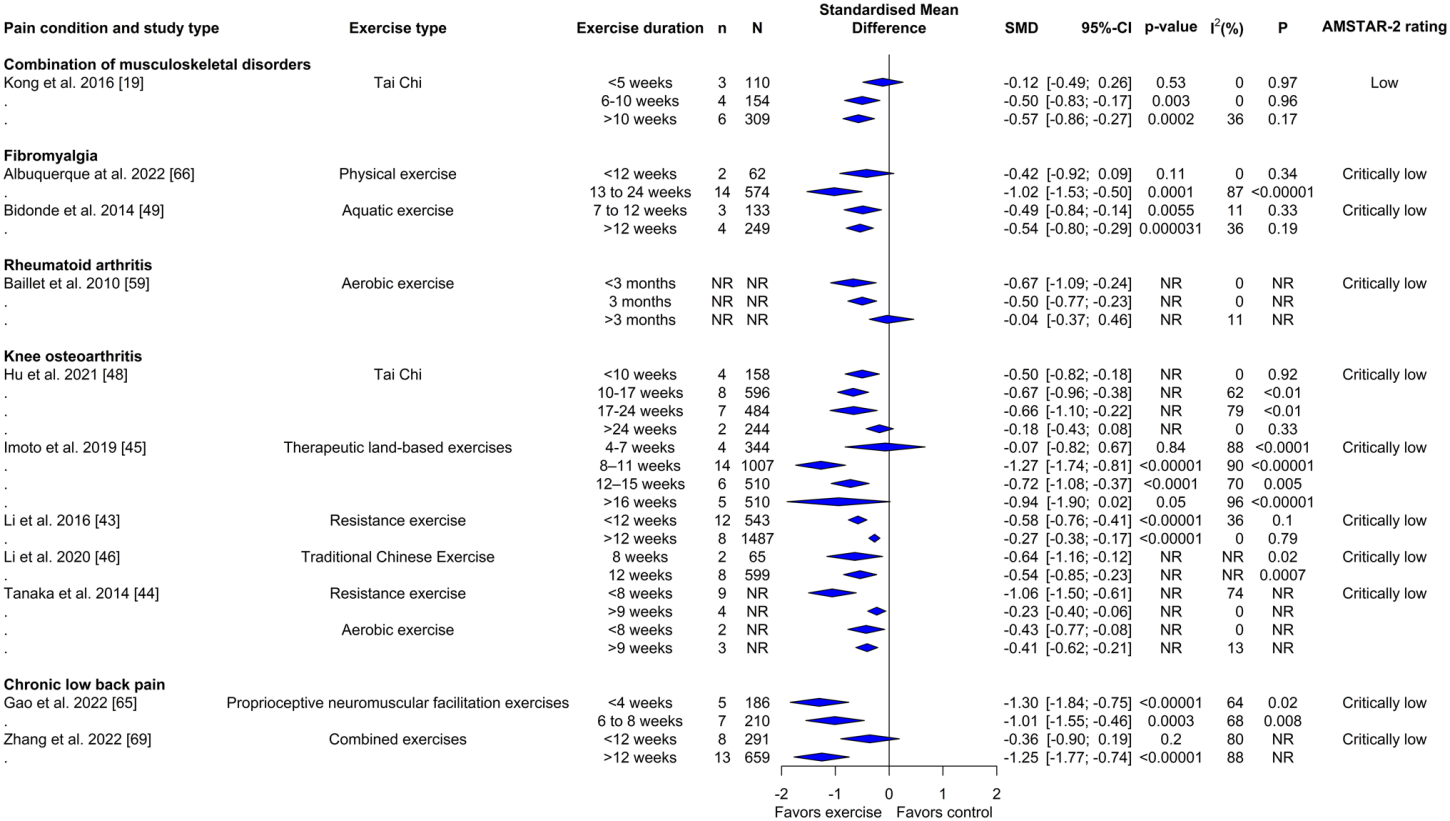
Forest plot demonstrating the effects of exercise duration in reviews of different musculoskeletal pain conditions for change in pain intensity outcome. Standardized mean differences (SMDs) and other data are as reported in the meta-analyses of individual studies. The results are reverse scaled and negative values indicate an improvement with exercise interventions. See supplementary file 11 in the electronic supplementary material for results from meta-regressions. *95%-CI* 95% confidence interval, *n* Number of studies in meta-analysis, *N* number of participants in meta-analysis, *NR* not reported, *P p*-value for the chi-square test of the null hypothesis stating that there is no heterogeneity

**Fig. 6:**
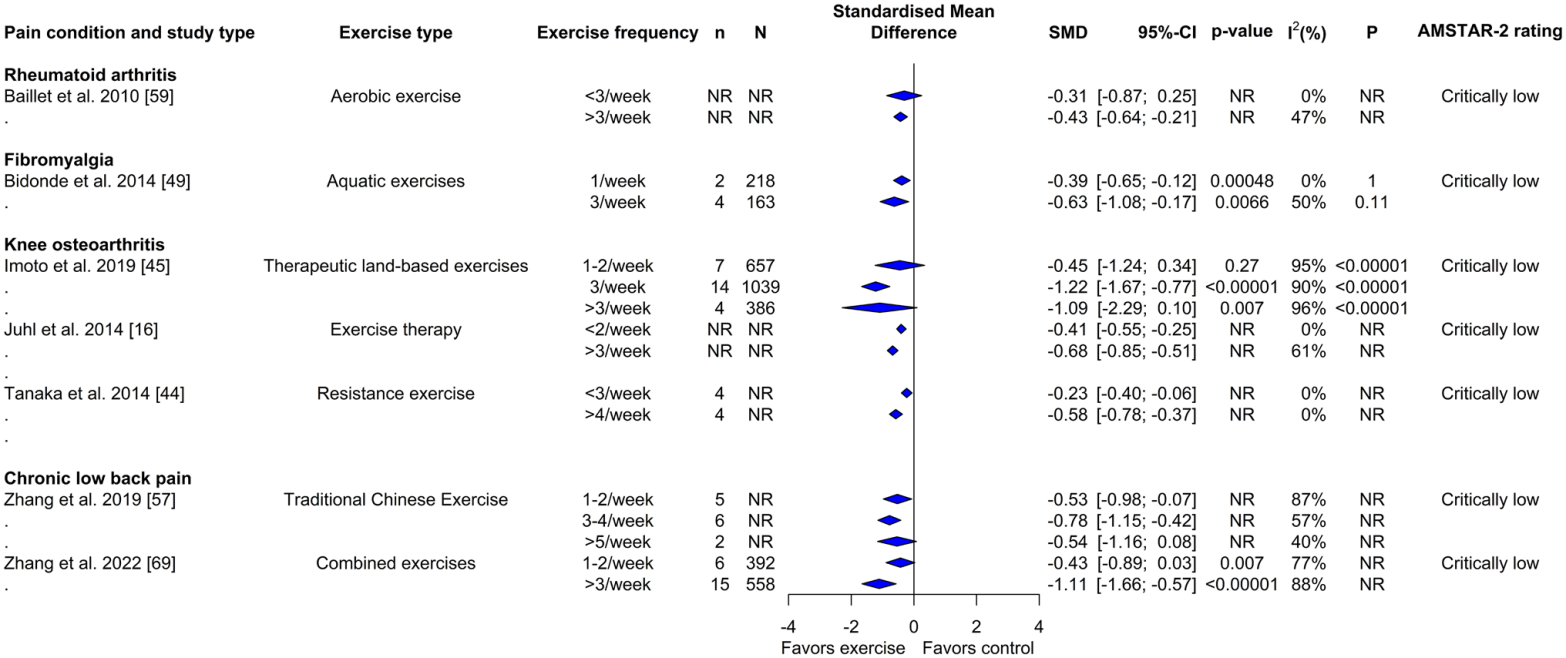
Forest plot demonstrating the effects of exercise frequency in reviews of different musculoskeletal pain conditions for change in pain intensity outcome. Standardized mean differences (SMDs) and other data are as reported in the meta-analyses of individual studies. The results are reverse scaled and negative values indicate an improvement with exercise interventions. See supplementary file 11 in the electronic supplementary material for results from meta-regressions. *95%-CI* 95% confidence interval, *n* Number of studies in meta-analysis, *N* number of participants in meta-analysis, *NR* not reported, *P p*-value for the chi-square test of the null hypothesis stating that there is no heterogeneity

Of the 20 studies quantitatively assessing the role of exercise dose on physical function, 18/20 (90%) examined duration (Fig. 3), 8/20 (40%) examined the frequency (Fig. 4), and 4/20 (20%) examined the intensity of exercise training sessions (Supplementary File 9 and 10, see ESM). Of the 25 studies that performed quantitative analyses of exercise dose for alteration of pain intensity, 24/25 (96%) examined exercise duration (Fig. 5), 13/25 (52%) examined exercise frequency (Fig. 6), and 2/25 (8%) examined the intensity of exercise training (Supplementary File 9 and 11, see ESM). One of the two studies that conducted a quantitative analysis of the effects of exercise on mental health examined the aspects of duration and frequency, while both reviews analyzed the impact of exercise intensity. None of the reviews analyzed the impact of exercise dose on adverse events and adherence rate.

#### Physical Function

Subgroup analysis (Fig. 3) and meta-regressions (Supplementary File 10, see ESM) with different exercise duration showed medium to large effect estimates of Tai Chi [SMD: − 1.05; 95% CI − 1.31 to − 0.79; 10–17 weeks], Tai Chi and Baduanjin exercises [SMD: − 0.78; 95% CI − 1.06 to − 0.50; 12 weeks] and resistance exercise [SMD: − 0.67; 95% CI − 0.84 to − 0.50; < 12 weeks] for knee osteoarthritis. Resistance [SMD: − 0.19; 95% CI − 0.29 to − 0.09; < 6 weeks] and aerobic [SMD: − 0.52; 95% CI − 0.95 to − 0.09; < 13 weeks] exercises produced small to large improvements in disability in rheumatoid arthritis. Likewise, proprioceptive neuromuscular facilitation exercises [SMD: − 1.60; 95% CI − 2.02 to − 1.18; 6–8 weeks] and combined exercise programs (aerobic, resistance, mind–body exercises and aquatic exercises) [SMD: − 2.52; 95% CI − 3.92 to − 1.12; > 12 weeks] showed a large effect size for improvements in disability in individuals with low back pain.

In subgroup analysis (Fig. 4) and meta-regressions (Supplementary File 10, see ESM) with different exercise frequencies, there was a small to moderate improvement in knee pain and rheumatoid arthritis with aerobic and resistance exercises [SMD: − 0.67; 95% CI − 0.89 to − 0.44; > 3/week] and aerobic exercises alone [SMD: − 0.26; 95% CI − 0.41 to − 0.11; > 3/week], respectively. Large effects were observed in disability for individuals with low back pain following a combined exercise training program (aerobic, resistance, mind–body exercises and aquatic exercises) [SMD: − 2.33; 95% CI − 3.74 to − 0.92; > 3/week].

#### Pain Intensity

Subgroup analysis (Fig. 5) and meta-regressions (Supplementary File 11, see ESM) with different exercise durations showed small to large effect estimates in reviews of knee osteoarthritis examining Tai Chi and Baduanjin exercise [SMD: − 0.64; 95% CI − 1.16 to − 0.12; 8 weeks], resistance [SMD: − 1.06; 95% CI − 1.50 to − 0.61; < 8 weeks], aerobic [SMD: − 0.43; 95% CI − 0.77 to − 0.08; < 8 weeks], therapeutic land-based exercises [SMD: − 1.27; 95% CI − 1.74 to − 0.81; 8–11 weeks] and Tai Chi alone [SMD: − 0.67; 95% CI − 0.96 to − 0.38; 10–17 weeks]. For fibromyalgia, aquatic exercises [SMD: − 0.54; 95% CI − 0.8 to − 0.29; > 12 weeks] and physical exercises [SMD: − 1.02; 95% CI − 1.53 to − 0.50; 13–24 weeks] led to an effect of medium to large magnitude. For rheumatoid arthritis, aerobic exercises for < 3 months’ duration led to a medium effect [SMD: − 0.67; 95% CI − 1.09 to − 0.24]. For low back pain, proprioceptive neuromuscular facilitation exercises [SMD: − 1.30; 95% CI − 1.84 to − 0.75; < 4 weeks] and combined exercise training program (aerobic, resistance, mind–body exercises and aquatic exercises) [SMD: − 1.25; 95% CI − 1.77 to − 0.74; > 12 weeks] were found to have a large effect estimate.

Subgroup analysis (Fig. 6) and meta-regressions (Supplementary File 11, see ESM) with different exercise frequencies showed medium to large effect estimates for therapeutic land-based exercises [SMD: − 1.22; 95% CI − 1.67 to − 0.77; 3/week], aerobic and resistance exercises [SMD: − 0.68; 95% CI − 0.85 to − 0.51; > 3/week] and resistance exercise alone [SMD: − 0.58; 95% CI − 0.78 to − 0.37; > 4/week] for knee osteoarthritis. Aquatic [SMD: − 0.63; 95% CI − 1.08 to − 0.17; 3/week] and aerobic [SMD: − 0.43; 95% CI − 0.64 to − 0.21; > 3/week] exercises produced small to medium improvements in pain intensity in fibromyalgia and rheumatoid arthritis, respectively. Frequency of traditional Chinese exercises [SMD: − 0.78; 95% CI − 1.15 to − 0.42; 3–4/week] and combined exercise training (aerobic, resistance, mind–body exercises and aquatic exercises) [SMD: − 1.11; 95% CI − 1.66 to − 0.57; > 3/week] was also found to be a predictor for improvements of pain intensity in low back pain.

#### Mental Health

A univariate moderator analysis demonstrated higher mental health improvements in fibromyalgia with exercise training programs conducted four times a week for more than 26 weeks compared with a lower dose (1–3 sessions per week for 13–26 weeks).

### Results of Individual Studies: Overlap in Primary Studies

We performed an overlap analysis across the 30 systematic reviews of musculoskeletal pain that quantitatively assessed the relationship between exercise dose and the outcome. These 30 reviews included 460 primary studies in meta-analysis. The covered area for the 30 systematic reviews and meta-analyses assessing dose prescription was 4.6% and the corrected covered area was 1.34%, indicating a slight degree of overlap. The median overlap in citations was 11.1% (range 0–37.5%) between knee pain reviews and 12% (range 9.7–18.2%) for reviews of fibromyalgia. We also plotted a citation matrix (available here) and heat map to assess the overlap in individual reviews in different pain conditions (overlap analysis heat map and sequential pair-wise comparisons) (Supplementary File 12a and b, see ESM). Based on the corrected covered area method, we found that knee pain reviews had the highest number of comparisons (11 out of 28 comparison pairs) with a ‘very high’ degree of primary study overlap (more than 15% overlap), indicating the repeated use of the same primary studies in multiple reviews under consideration. Only one pair (out of 55 comparison pairs) of low back pain and one pair (out of three comparison pairs) of fibromyalgia comparisons had a ‘very high’ overlap. The reviews assessing a combination of pain conditions presented with a ‘very high’ overlap of primary studies with a knee pain review (one out of 16 comparison pairs). ‘High’ overlap (11% to 15%) was present in knee pain reviews (five out of 28 comparison pairs) and also with knee pain reviews compared with reviews assessing a combination of pain conditions (two out of 16 comparison pairs). The other reviews presented with either a ‘moderate’ (14 pairs of comparison) or ‘slight’ (400 pairs of comparison) degree of overlap in primary studies. Detailed information about the results of sequential comparisons is available in Supplementary File 12b (see ESM).

### Protocol Amendments Since PROSPERO Registration

We removed ROBIS (Risk of Bias Assessment Tool for Systematic Reviews) [70] and only used AMSTAR-2 for the assessment of methodological quality given considerable overlap between the rating items in the two tools. Moreover, AMSTAR-2 has a higher inter-rater reliability (IRR 0.49) than ROBIS (IRR 0.09–0.38) [71]. ROBIS also lacks questions related to ‘conflict of interest’ and ‘reason for excluding studies’ that are potential sources of bias in a review [72]. We elected not to extract data on follow-up duration because they were not clearly and consistently reported in the included reviews. In our PROSPERO registration, we stated that we would only include reviews that conducted a quantitative analysis of the impact of exercise prescription variables on musculoskeletal pain outcomes. However, in order to encompass a broader range of literature and include all pertinent information regarding exercise reporting and prescription, we expanded the inclusion criteria in our protocol and included all the available systematic reviews and meta-analyses for musculoskeletal pain conditions. By expanding our criteria, we were able to incorporate the total volume of the available evidence and hence evaluate the quality of exercise prescription reporting in the musculoskeletal pain literature more widely.

## Discussion

This is the first umbrella review of available systematic reviews and meta-analyses literature to evaluate the reporting and quality of evidence for exercise prescription in individuals with musculoskeletal pain to improve intervention outcomes. Our umbrella review showed that only a small proportion of the available evidence analyzed the impact of exercise dose on outcomes. Furthermore, the results of these reviews should be interpreted with caution given most were of ‘critically low’ quality.

The publication of seven reviews with a ‘high’ methodological quality in recent years suggests an improvement in review quality over time, which may in part be due to many journals adopting reporting guidelines such as PRISMA [73]. However, most of the recent reviews were still rated as having a ‘critically low’ methodological quality. The poor quality of reviews could be attributed to the use of non-standard approaches in the conduct and reporting of the reviews, thereby leading to a lower score on the AMSTAR-2 criteria. Another possible explanation for the poor methodological quality could be the word limit of journals that leads to the reporting of only part of the methodology. However, as the AMSTAR-2 tool requires the review authors to report all the items in detail to obtain a higher rating, these reviews would have achieved a lower score if the reporting was not good, even if they might have followed all the steps as per the standard recommendations [24].

Of the 30 reviews that extracted and reported information on exercise dose, the most commonly analyzed exercise prescription variables were duration, frequency and intensity, in 86.7%, 50% and 23.3% of reviews, respectively. There is a need to also examine exercise volume, which was reported in 21.2% of the included reviews but was not analyzed for an interaction with outcomes. The poor reporting and analysis of exercise intensity and volume in the reviews could be related to the unclear operational definitions in the included primary studies. To improve the reporting of exercise interventions, TIDieR (Template for Intervention Description and Replication) [74] and CERT (Consensus on Exercise Reporting Template) [75] checklists were published in the years 2014 and 2016, respectively. Consistent with that, 26 out of 30 reviews that analyzed exercise dose were published after 2014, indicating an improvement in reporting following the introduction of these reporting checklists. In line with these results, scientific journals should mandate the use of TIDieR and CERT checklists as this could be a helpful step in improving the reporting quality of exercise trials, thereby improving the overall systematic review evidence [76].

### Physical Function

Reviews assessing physical function used an exercise duration of < 4 weeks to > 24 weeks and a frequency of once a week to more than three times per week. However, a duration of up to 17 weeks and frequency of three or more sessions per week was shown to result in an improvement of small to large effect size. Our review's findings align with the current literature that suggests an improvement in physical function with more frequent exercise training [77].

### Pain Intensity

Reviews assessing pain intensity used an exercise duration of 4 weeks to > 24 weeks; however, a duration range of < 4 weeks up to 24 weeks led to an improvement of small to large effect size. The frequency ranged from once a week to more than five sessions per week, but a frequency of three or more sessions per week showed an improvement of small to large effect size. This is consistent with the previous evidence that suggests using a longer and more frequent exercise intervention for an effective reduction in pain intensity [12]. The improvement in pain and physical function with a similar dose was in line with previous evidence that suggested a strong association between pain reduction and improvement of physical function [78].

Systematic reviews of both unimodal and multimodal exercise interventions showed significant improvements in pain and physical function, whereas mental health improved in studies that assessed a multimodal exercise intervention. The observed differences in the treatment effects might be attributed to the combination of a wide variety of exercises in different reviews that might lead to inconsistency in the findings of these reviews. A large proportion of reviews (50%) focused on knee and low back pain, with knee osteoarthritis (17.9%) and non-specific low back pain (9.9%) being the most commonly assessed conditions in the available musculoskeletal pain literature. None of the reviews assessed exercise prescription variables in studies of hand, elbow or hip pain.

Primary studies overlapped substantially in most of the knee pain (mean overlap 12.3%) reviews according to the corrected covered area method. This means that, of the included reviews on knee pain, the included trials overlapped on average 12.3% between pairs of reviews. However, as the overall corrected covered area for the included reviews was low (1.34%), this indicates that the assessed reviews included unique primary studies and hence the available evidence can be trusted for the different pain conditions (i.e., populations).

A recent umbrella review summarized the reporting quality of exercise trials in chronic diseases and suggested that evidence is lacking for an ‘optimal’ exercise dose for these conditions [79]. The restrictive inclusion criteria imposed by the authors of this prior review led to the inclusion of only 23 reviews (i.e., reviews that used either the CERT or TIDIER checklist). An umbrella review by the Cochrane group examined the role of exercise and physical activity in chronic pain [12]. In this review, only Cochrane reviews were included (*N* = 21) and whilst exercise dose data were considered, information on the impact of exercise dose on outcomes was not available. Our current work extends these prior studies by including a wider body of evidence and capturing further systematic reviews analyzing exercise prescription in a more specific and clinically important research area (i.e., musculoskeletal pain).

### Limitations

Though we followed standardized criteria for conducting the review and reporting the findings, this review still has certain limitations. First, we relied on information provided by review authors in the articles and did not re-assess the GRADE rating for the included reviews. PRIOR guidelines recommend extracting the data from the review reports, but none of the included systematic reviews and meta-analyses provided a GRADE rating for the subgroup of analysis on exercise prescription. As a result, the certainty of the evidence for each outcome could not be assessed using GRADE [25]. Second, even though we performed a comprehensive search of multiple databases, we might have missed potential reviews because of limiting the search to English language [80]. However, Cochrane recommendations are equivocal concerning including English language studies and do not suggest any likelihood of bias in the review results as a result of including only these studies. Third, we summarized the data narratively in terms of numbers and percentages rather than performing a statistical analysis. However, the scope of this umbrella review was to summarize the quality of reviews that report exercise prescription in musculoskeletal disorders and identify any potential reviews that might have analyzed the role of exercise dose on musculoskeletal pain conditions. Fourth, we relied on the meta-analyses reported in the included reviews and did not extract or verify data from the original studies or re-run a meta-analysis, as this was beyond the scope of this umbrella review. Of note, exercise prescription data may have been available in the primary studies but not extracted by authors of the included reviews.

### Future Directions

We provide a systematic, comprehensive and detailed summary of the current evidence base. We found several research gaps that need to be addressed by future studies in order to improve the exercise-based management of musculoskeletal disorders. The results of this review highlight the importance of improving the reporting of exercise interventions as this would help to better translate research evidence to clinical practice. Future research synthesis should extract exercise dose data and utilize relevant network meta-analytic methods for assessing the dose–response relationship in RCTs [81, 82]. These analysis methods could be applied to the current research question without the limitations of meta-regression that typically assumes dose–response in exercise to be linear. Further, future reviews should focus on performing and reporting methods in line with AMSTAR-2 criteria, and assessment of exercise dose prescription in mental health outcomes, adherence and adverse events would add to the current body of literature. Future studies should analyze exercise dose across chronic musculoskeletal pain conditions whilst considering relevant sub-group analyses, as the impact of exercise dose may be consistent for a number of diagnoses [83]. Finally, a living systematic review could also be considered in future studies in order to tackle the increasing evidence in the field of musculoskeletal rehabilitation, thereby reducing duplication and research waste [84].

## Conclusion

There is limited evidence that quantifies the ideal exercise dose for improving clinical outcomes in musculoskeletal pain disorders. The overall quality of systematic reviews needs to be improved to derive stronger recommendations. Additionally, future primary studies should focus on improving the reporting of exercise variables, while systematic reviews should focus on analyzing these exercise prescription variables in greater detail to identify optimal, conservative and cost-effective solutions for the ever-increasing prevalence of musculoskeletal pain disorders. More rigorous and robust systematic reviews and meta-analyses are required before making any recommendations for an ‘optimal’ dose of exercise for musculoskeletal pain conditions.

### Supplementary Information

Below is the link to the electronic supplementary material.

Supplementary file1 (DOCX 6330 KB)

## Declarations

### Availability of Data and Material

The data that support the findings of this review are freely available https://osf.io/hgu9p/?view_only=c6958c60516e49fe81bad83f1f4659f0.

### Code Availability

R code for plotting forest plots and AMSTAR-2 figures can be accessed via this https://osf.io/hgu9p/?view_only=c6958c60516e49fe81bad83f1f4659f0.
