# Supplementary material for: The Impact of Exercise Prescription Variables on Intervention Outcomes in Musculoskeletal Pain: An Umbrella Review of Systematic Reviews
Source: Sports Med. 2023 Dec 14;54(3):711–25. doi: 10.1007/s40279-023-01966-2 (PMC10978700; doi:10.1007/s40279-023-01966-2)
Supplement: Supplementary file 1 — Supplementary file1 (DOCX 6330 KB) [file 40279_2023_1966_MOESM1_ESM.docx]

**SUPPLEMENTARY MATERIAL**

**The impact of exercise prescription variables on intervention outcomes in musculoskeletal pain: umbrella review of systematic reviews**

**SPORTS MEDICINE**

Nitin Kumar Arora ^1,2^, Lars Donath ^2^ , Patrick J Owen ^3^, Clint T Miller ^3^, Tobias Saueressig ^4^, Felicitas Winter ^1^, Marina Hambloch ^1^, Christopher Neason ^3^, Vera Karner ^1^, Daniel L Belavy ^1^

^1^ Hochschule für Gesundheit [University of Applied Sciences], Department of Applied Health Sciences, Division of Physiotherapy, Bochum, Germany

^2^ German Sport University Cologne, Department of Intervention Research in Exercise Training, Cologne, Germany

^3^ Deakin University, Institute for Physical Activity and Nutrition (IPAN), School of Exercise and Nutrition Sciences, Geelong, Victoria, Australia

^4^ Science and Research, Physio Meets Science GmbH, Leimen, Baden-Württemberg, Germany

**Table of contents**

[Supplementary file 1. PRIOR checklist 3](#_Toc139843449)

[Supplementary file 2. Search strategy 9](#_Toc139843450)

[Supplementary file 3. Exclusion criteria as list 19](#_Toc139843451)

[Supplementary file 4. List of studies excluded at full-text review with reasons 24](#_Toc139843452)

[Supplementary file 5. Characteristics of included reviews 60](#_Toc139843453)

[Supplementary file 6. References list of included studies 88](#_Toc139843454)

[Supplementary file 7. AMSTAR-2 ratings for included reviews 113](#_Toc139843455)

[Supplementary file 8. Narrative summary of the included reviews (per diagnosis) 123](#_Toc139843456)

[Supplementary file 9. Narrative summary of the included reviews (exercise dose prescription per diagnosis) 127](#_Toc139843457)

[Supplementary file 10. Meta-regression of exercise dose in different pain conditions (Physical function outcome) 130](#_Toc139843458)

[Supplementary file 11. Meta-regression of exercise dose in different pain conditions (pain outcome) 131](#_Toc139843459)

[Supplementary file 12a. Heat map for primary study overlap analysis 132](#_Toc139843460)

[Supplementary file 12b. Sequential pairwise comparisons for primary study overlap analysis 133](#_Toc139843461)

# Supplementary file 1. PRIOR checklist

| **Section**  Topic | **#** | **Item** | **Location reported** |
| --- | --- | --- | --- |
| **TITLE** | | |  |
| Title | 1 | Identify the report as an overview of reviews. | 1 |
| **ABSTRACT** | | |  |
| Abstract | 2 | Provide a comprehensive and accurate summary of the purpose, methods, and results of the overview of reviews. | 3 |
| **INTRODUCTION** | | |  |
| Rationale | 3 | Describe the rationale for conducting the overview of reviews in the context of existing knowledge. | 6-7 |
| Objectives | 4 | Provide an explicit statement of the objective(s) or question(s) addressed by the overview of reviews. | 7 |
| **METHODS** | | |  |
| Eligibility criteria | 5a | Specify the inclusion and exclusion criteria for the overview of reviews. If supplemental primary studies were included, this should be stated, with a rationale. | 8 |
|  | 5b | Specify the definition of ‘systematic review’ as used in the inclusion criteria for the overview of reviews. | 8 |
| Information sources | 6 | Specify all databases, registers, websites, organizations, reference lists, and other sources searched or consulted to identify systematic reviews and supplemental primary studies (if included).  Specify the date when each source was last searched or consulted. | 8 |
| Search strategy | 7 | Present the full search strategies for all databases, registers and websites, such that they could be reproduced. Describe any search filters and limits applied. | SF-2 |
| Selection process | 8a | Describe the methods used to decide whether a systematic review or supplemental primary study (if included) met the inclusion criteria of the overview of reviews. | 9 |
|  | 8b | Describe how overlap in the populations, interventions, comparators, and/or outcomes of systematic reviews was identified and managed during study selection. | 9 |
| Data collection process | 9a | Describe the methods used to collect data from reports. | 9 |
|  | 9b | If applicable, describe the methods used to identify and manage primary study overlap at the level  of the comparison and outcome during data collection. For each outcome, specify the method used to illustrate and/or quantify the degree of primary study overlap across systematic reviews. | 10 |
|  | 9c | If applicable, specify the methods used to manage discrepant data across systematic reviews during data collection. | NA |
| Data items | 10 | List and define all variables and outcomes for which data were sought. Describe any assumptions made and/or measures taken to identify and clarify missing or unclear information. | 9-10 |
| Risk of bias assessment | 11a | Describe the methods used to *assess* risk of bias or methodological quality of the included systematic reviews. | 10 |
|  | 11b | Describe the methods used to *collect* data on (from the systematic reviews) and/or *assess* the risk of bias of the primary studies included in the systematic reviews. Provide a justification for instances where flawed, incomplete, or missing assessments are identified but not re-assessed. | 10 |
|  | 11c | Describe the methods used to *assess* the risk of bias of supplemental primary studies (if included). | NA |
| Synthesis methods | 12a | Describe the methods used to summarize or synthesize results and provide a rationale for the choice(s). | 10-11 |
|  | 12b | Describe any methods used to explore possible causes of heterogeneity among results. | No meta-analysis |
|  | 12c | Describe any sensitivity analyses conducted to assess the robustness of the synthesized results. | No meta-analysis |
| Reporting bias assessment | 13 | Describe the methods used to *collect* data on (from the systematic reviews) and/or *assess* the risk of bias due to missing results in a summary or synthesis (arising from reporting biases at the levels of the systematic reviews, primary studies, and supplemental primary studies, if included). | NA |
| Certainty assessment | 14 | Describe the methods used to *collect* data on (from the systematic reviews) and/or *assess* certainty (or confidence) in the body of evidence for an outcome. | NA |
| **RESULTS** | | |  |
| Systematic review and supplemental primary study selection | 15a | Describe the results of the search and selection process, including the number of records screened, assessed for eligibility, and included in the overview of reviews, ideally with a flow diagram. | 12 |
|  | 15b | Provide a list of studies that might appear to meet the inclusion criteria, but were excluded, with the main reason for exclusion. | SF-4 |

| **Section**  Topic | **#** | **Item** | **Location**  **reported** |  |
| --- | --- | --- | --- | --- |
| Characteristics of systematic reviews and  supplemental primary studies | 16 | Cite each included systematic review and supplemental primary study (if included) and present its characteristics. | 12-15 and SF-5, 8 and 9 |  |
| Primary study overlap | 17 | Describe the extent of primary study overlap across the included systematic reviews. | 15-16 |  |
| Risk of bias in systematic reviews, primary studies, and  supplemental primary studies | 18a | Present assessments of risk of bias or methodological quality for each included systematic review. | 12 |  |
|  | 18b | Present assessments (*collected* from systematic reviews or *assessed* anew) of the risk of bias of the primary studies included in the systematic reviews. | NA |  |
|  | 18c | Present assessments of the risk of bias of supplemental primary studies (if included). | NA |  |
| Summary or synthesis of results | 19a | For all outcomes, summarize the evidence from the systematic reviews and supplemental primary studies (if included). If meta-analyses were done, present for each the summary estimate and its precision and measures of statistical heterogeneity. If comparing groups, describe the direction of the effect. | 13, 14, and 15 & SF-10 |  |
|  | 19b | If meta-analyses were done, present results of all investigations of possible causes of heterogeneity. | No meta-analysis |  |
|  | 19c | If meta-analyses were done, present results of all sensitivity analyses conducted to assess the robustness of synthesized results. | No meta-analysis |  |
| Reporting biases | 20 | Present assessments (*collected* from systematic reviews and/or *assessed* anew) of the risk of bias due to missing primary studies, analyses, or results in a summary or synthesis (arising from reporting biases at the levels of the systematic reviews, primary studies, and supplemental primary  studies, if included) for each summary or synthesis assessed. | NA |  |
| Certainty of  evidence | 21 | Present assessments (*collected* or *assessed* anew) of certainty (or confidence) in the body of evidence for each outcome. | NA |  |
| **DISCUSSION** | | |  |  |
| Discussion | 22a | Summarize the main findings, including any discrepancies in findings across the included systematic reviews and supplemental primary studies (if included). | 17-19 |  |
|  | 22b | Provide a general interpretation of the results in the context of other evidence. | 17-19 |  |
|  | 22c | Discuss any limitations of the evidence from systematic reviews, their primary studies, and supplemental primary studies (if included) included in the overview of reviews. Discuss any  limitations of the overview of reviews methods used. | 19 |  |
|  | 22d | Discuss implications for practice, policy, and future research (both systematic reviews and primary research). Consider the relevance of the findings to the end users of the overview of reviews, e.g., healthcare providers, policymakers, patients, among others. | 19-20 |  |
| **OTHER INFORMATION** | | |  |  |
| Registration and protocol | 23a | Provide registration information for the overview of reviews, including register name and registration number, or state that the overview of reviews was not registered. | 3 |  |
|  | 23b | Indicate where the overview of reviews protocol can be accessed, or state that a protocol was not prepared. | NA |  |
|  | 23c | Describe and explain any amendments to information provided at registration or in the protocol. Indicate the stage of the overview of reviews at which amendments were made. | 16 |  |
| Support | 24 | Describe sources of financial or non-financial support for the overview of reviews, and the role of the funders or sponsors in the overview of reviews. | 5 |  |
| Competing interests | 25 | Declare any competing interests of the overview of reviews' authors. | 5 |  |
| Author information | 26a | Provide contact information for the corresponding author. | 1 |  |
|  | 26b | Describe the contributions of individual authors and identify the guarantor of the overview of reviews. | 5 |  |
| Availability of data and other materials | 27 | Report which of the following are available, where they can be found, and under which conditions they may be accessed: template data collection forms; data collected from included systematic reviews and supplemental primary studies; analytic code; any other materials used in the overview of reviews. | 5, 9, 10, 15 |  |

Abbreviations: SF, supplementary file; NA, not applicable

# Supplementary file 2. Search strategy

Date of database search: 01.10.2021

**SPORTDiscus via EBSCOHost**

|  | Search | Query | Hits |
| --- | --- | --- | --- |
| Population | #1 | (DE "PAIN"): Explode all terms | 30315 |
|  | #2 | TI pain OR AB pain | 56212 |
|  | #3 | #1 OR #2 | 63872 |
| Intervention/  Comparator | #4 | (((DE "EXERCISE therapy") OR (DE "EXERCISE")) | 94596 |
|  | #5 | (TI ( (exercise* OR ‘kinesiotherapy’ OR strength* OR isometric* OR isokinetic* OR aerobic* OR endurance OR weigh* OR resistance OR high intens* OR low intens* OR low impact OR high impact OR dose* OR walk* OR treadmill* OR cycl* OR gait) ) OR AB ( (exercise* OR ‘kinesiotherapy’ OR strength* OR isometric* OR isokinetic* OR aerobic* OR endurance OR weigh* OR resistance OR high intens* OR low intens* OR low impact OR high impact OR dose* OR walk* OR treadmill* OR cycl* OR gait) )) AND (TI ( train* OR exercise ) OR AB ( train* OR exercise ) ) | 188157 |
|  | #6 | #4 OR #5 | 221673 |
|  | #7 | TI ( (“cochrane review” OR “systematic review” OR “meta analysis") ) OR AB ( (“cochrane review” OR “systematic review” OR “meta analysis") ) OR TI review | 40845 |
|  | #8 | #3 AND #6 AND #7 | 729 |
|  | #9 | (DE “Surgery” OR DE “Cancer”): Explode all terms | 28187 |
|  | #10 | S8 NOT S9 | 712 |
| Filters/Limits | #11 | Filter: English, Academic journals | 669 |

**PubMed**

|  | Search | Query | Hits |
| --- | --- | --- | --- |
| Population | #1 | ("Pain"[MeSH]) | 419987 |
|  | #2 | (pain[Title/Abstract]) | 684931 |
|  | #3 | #1 OR #2 | 843376 |
| Intervention/  Comparator | #4 | exercise [MeSH] OR ‘exercise therapy’[MeSH] | 251951 |
|  | #5 | (exercis*[Title/Abstract] OR ‘kinesiotherapy’[Title/Abstract])  OR ((strength*[Title/Abstract] OR isometric*[Title/Abstract] OR isokinetic*[Title/Abstract] OR aerobic*[Title/Abstract] OR endurance[Title/Abstract] OR weigh*[Title/Abstract] OR resistance[Title/Abstract] OR high intens*[Title/Abstract] OR low intens*[Title/Abstract] OR low impact[Title/Abstract] OR high impact[Title/Abstract] OR dose*[Title/Abstract] OR walk*[Title/Abstract] OR treadmill*[Title/Abstract] OR cycl*[Title/Abstract] OR gait[Title/Abstract])  AND (train*[Title/Abstract] OR exercise[Title/Abstract])) | 413740 |
|  | #6 | #4 OR #5 | 528338 |
|  | #7 | Cochrane review [Title/Abstract] OR Systematic review* [Title/Abstract] OR meta?analysis [Title/Abstract] OR review* [Title] | 712538 |
|  | #8 | #3 AND #6 AND #7 | 2863 |
|  | #9 | (animal[MeSH Terms]) NOT human[MeSH Terms] | 4892888 |
|  | #10 | #8 NOT #9 | 2855 |
|  | #11 | **(("Cancer Pain"[Mesh])) OR (("Pain, Postoperative"[Mesh]))** | 46649 |
|  | #12 | #10 NOT #11 | 2823 |
| Filters/Limits | #13 | Filters: Meta-Analysis, Review, Systematic Review, English | 2280 |

**EMBASE via OVID**

| Population | #1 | exp pain/ | 1435353 |
| --- | --- | --- | --- |
|  | #2 | pain.ti. or pain.ab. | 1005594 |
|  | #3 | 1 OR 2 | 1717142 |
| Intervention/  Comparator | #4 | exp exercise/ OR exp kinesiotherapy/ | 418673 |
|  | #5 | ((exercise* or kinesiotherapy or strength* or isometric* or isokinetic* or aerobic* or endurance or weigh* or resistance or high intens* or low intens* or low impact or high impact or dose* or walk* or treadmill* or cycl* or gait).ti. or (exercise* or kinesiotherapy or strength* or isometric* or isokinetic* or aerobic* or endurance or weigh* or resistance or high intens* or low intens* or low impact or high impact or dose* or walk* or treadmill* or cycl* or gait).ab.) and ((train* or exercise).ti. or (train* or exercise).ab.) | 505208 |
|  | #6 | 4 OR 5 | 656069 |
|  | #7 | 3 AND 6 | 67963 |
|  | #8 | (((Cochrane review or systematic review or meta analysis).ti OR (Cochrane review or systematic review or meta analysis).ab) OR (review.ti.)) | 814376 |
|  | #9 | 7 AND 8 | 4607 |
|  | #10 | exp surgery/ | 5166294 |
|  | #11 | cancer.mp. or exp malignant neoplasm/ | 4812813 |
|  | #12 | 10 OR 11 | 8715299 |
|  | #13 | 9 NOT 12 | 9828 |
|  | #14 | (animal not human).af | 4300560 |
|  | #15 | 13 NOT 14 | 9773 |
| Filters/Limits | #16 | Limiters: Human, English Language, Meta analysis, Systematic Review, Journal | 2379 |
|  | #17 | Limiters: Exclude MEDLINE | 229 |

**CINAHL with EBSCOHost**

|  | Search | Terminology | Hits |
| --- | --- | --- | --- |
| Population | #1 | (MH "Pain+") | 219208 |
|  | #2 | TI Pain OR AB Pain | 270325 |
|  | #3 | #1 OR #2 | 347201 |
| Intervention/  Comparator | #4 | MH ("Exercise+" OR "Therapeutic Exercise+") | 145347 |
|  | #5 | (TI ( (exercise* OR ‘kinesiotherapy’ OR strength* OR isometric* OR isokinetic* OR aerobic* OR endurance OR weigh* OR resistance OR high intens* OR low intens* OR low impact OR high impact OR dose* OR walk* OR treadmill* OR cycl* OR gait) ) AND AB ( (exercise* OR ‘kinesiotherapy’ OR strength* OR isometric* OR isokinetic* OR aerobic* OR endurance OR weigh* OR resistance OR high intens* OR low intens* OR low impact OR high impact OR dose* OR walk* OR treadmill* OR cycl* OR gait)) AND (TI ( train* OR exercise ) OR AB ( train* OR exercise ))) | 50177 |
|  | #6 | #4 OR #5 | 163421 |
|  | #7 | (TI ( "Systematic Review" OR “Meta Analysis” OR “meta-analys*” ) OR AB ( "Systematic Review" OR “Meta Analysis” OR “meta-analys*”) OR (TI (Review))) | 291016 |
|  | #8 | #3 AND #6 AND #7 | 1130 |
|  | #9 | (MH "Surgery, Operative+") | 704463 |
|  | #10 | #8 NOT #19 | 1006 |
|  | #11 | (MH "Animals+") NOT (MH "Human") | 89163 |
|  | #12 | #10 NOT #11 | 1003 |
| Filters/Limits | #13 | Filter: English, Academic Journals | 917 |

**COCHRANE**

|  | Search | Query | Hits |
| --- | --- | --- | --- |
| Population | #1 | MeSH descriptor: [Pain] explode all trees | 52513 |
|  | #2 | ("pain"):ti,ab,kw | 195141 |
|  | #3 | #1 OR #2 | 201499 |
| Intervention/  Comparator | #4 | MeSH descriptor: [Exercise] explode all trees | 26665 |
|  | #5 | MeSH descriptor: [Exercise Movement Techniques] explode all trees | 2330 |
|  | #6 | MeSH descriptor: [Exercise Therapy] explode all trees | 15157 |
|  | #7 | (exercise* OR ‘kinesiotherapy’ OR strength* OR isometric* OR isokinetic* OR aerobic* OR endurance OR weigh* OR resistance OR train* OR high intens* OR low intens* OR low impact OR high impact OR dose* OR walk* OR treadmill* OR cycl* OR gait):ti,ab,kw | 724264 |
|  | #8 | #4 OR #5 OR #6 OR #7 | 724817 |
|  | #9 | #3 AND #8 | 89062 |
| Filters/Limits | #10 | *Limits: Cochrane reviews* | 1385 |

**Date of database search: 14.02.2023**

**SPORTDiscus via EBSCOHost**

|  | Search | Query | Hits |
| --- | --- | --- | --- |
| Population | #1 | (DE "PAIN"): Explode all terms | 32274 |
|  | #2 | TI pain OR AB pain | 59896 |
|  | #3 | #1 OR #2 | 67887 |
| Intervention/  Comparator | #4 | (((DE "EXERCISE therapy") OR (DE "EXERCISE")) | 96881 |
|  | #5 | (TI ( (exercise* OR ‘kinesiotherapy’ OR strength* OR isometric* OR isokinetic* OR aerobic* OR endurance OR weigh* OR resistance OR high intens* OR low intens* OR low impact OR high impact OR dose* OR walk* OR treadmill* OR cycl* OR gait) ) OR AB ( (exercise* OR ‘kinesiotherapy’ OR strength* OR isometric* OR isokinetic* OR aerobic* OR endurance OR weigh* OR resistance OR high intens* OR low intens* OR low impact OR high impact OR dose* OR walk* OR treadmill* OR cycl* OR gait) )) AND (TI ( train* OR exercise ) OR AB ( train* OR exercise ) ) | 196714 |
|  | #6 | #4 OR #5 | 230604 |
|  | #7 | TI ( (“cochrane review” OR “systematic review” OR “meta analysis") ) OR AB ( (“cochrane review” OR “systematic review” OR “meta analysis") ) OR TI review | 45073 |
|  | #8 | #3 AND #6 AND #7 | 884 |
|  | #9 | (DE “Surgery” OR DE “Cancer”): Explode all terms | 29073 |
|  | #10 | S8 NOT S9 | 866 |
| Filters/Limits | #11 | *Filter: English, Academic journals, Published Date: October 2021 to February 2023* | 146 |

**PubMed**

|  | Search | Query | Hits |
| --- | --- | --- | --- |
| Population | #1 | ("Pain"[MeSH]) | 450451 |
|  | #2 | (pain[Title/Abstract]) | 751736 |
|  | #3 | #1 OR #2 | 914185 |
| Intervention/  Comparator | #4 | exercise [MeSH] OR ‘exercise therapy’[MeSH] | 277071 |
|  | #5 | (exercis*[Title/Abstract] OR ‘kinesiotherapy’[Title/Abstract])  OR ((strength*[Title/Abstract] OR isometric*[Title/Abstract] OR isokinetic*[Title/Abstract] OR aerobic*[Title/Abstract] OR endurance[Title/Abstract] OR weigh*[Title/Abstract] OR resistance[Title/Abstract] OR high intens*[Title/Abstract] OR low intens*[Title/Abstract] OR low impact[Title/Abstract] OR high impact[Title/Abstract] OR dose*[Title/Abstract] OR walk*[Title/Abstract] OR treadmill*[Title/Abstract] OR cycl*[Title/Abstract] OR gait[Title/Abstract])  AND (train*[Title/Abstract] OR exercise[Title/Abstract])) | 456804 |
|  | #6 | #4 OR #5 | 582410 |
|  | #7 | Cochrane review [Title/Abstract] OR Systematic review* [Title/Abstract] OR meta?analysis [Title/Abstract] OR review* [Title] | 832393 |
|  | #8 | #3 AND #6 AND #7 | 3494 |
|  | #9 | (animal[MeSH Terms]) NOT human[MeSH Terms] | 5090840 |
|  | #10 | #8 NOT #9 | 3486 |
|  | #11 | **(("Cancer Pain"[Mesh])) OR (("Pain, Postoperative"[Mesh]))** | 51108 |
|  | #12 | #10 NOT #11 | 3450 |
| Filters/Limits | #13 | Filters: Meta-Analysis, Review, Systematic Review, English, , from 2021/10/1 - 2023/2/14 | 616 |

**EMBASE via OVID**

|  | Search | Query | Hits |
| --- | --- | --- | --- |
| Population | #1 | exp pain/ | 1585942 |
|  | #2 | pain.ti. or pain.ab. | 1112145 |
|  | #3 | 1 OR 2 | 1892781 |
| Intervention/  Comparator | #4 | exp exercise/ OR exp kinesiotherapy/ | 464152 |
|  | #5 | ((exercise* or kinesiotherapy or strength* or isometric* or isokinetic* or aerobic* or endurance or weigh* or resistance or high intens* or low intens* or low impact or high impact or dose* or walk* or treadmill* or cycl* or gait).ti. or (exercise* or kinesiotherapy or strength* or isometric* or isokinetic* or aerobic* or endurance or weigh* or resistance or high intens* or low intens* or low impact or high impact or dose* or walk* or treadmill* or cycl* or gait).ab.) and ((train* or exercise).ti. or (train* or exercise).ab.) | 559742 |
|  | #6 | 4 OR 5 | 726969 |
|  | #7 | 3 AND 6 | 76400 |
|  | #8 | (((Cochrane review or systematic review or meta analysis).ti OR (Cochrane review or systematic review or meta analysis).ab) OR (review.ti.)) | 957077 |
|  | #9 | 7 AND 8 | 5682 |
|  | #10 | exp surgery/ | 5587292 |
|  | #11 | cancer.mp. or exp malignant neoplasm/ | 5282281 |
|  | #12 | 10 OR 11 | 9490273 |
|  | #13 | 9 NOT 12 | 3911 |
|  | #14 | (animal not human).af | 4515881 |
|  | #15 | 13 NOT 14 | 3900 |
| Filters/Limits | #16 | Limiters: Human, English Language, Meta analysis, Systematic Review, Journal | 2381 |
|  | #17 | Limiters: Exclude MEDLINE, Published Date: October 2021 to February 2023 | 260 |

**CINAHL with EBSCOHost**

|  | Search | Terminology | Hits |
| --- | --- | --- | --- |
| Population | #1 | (MH "Pain+") | 235213 |
|  | #2 | TI Pain OR AB Pain | 297684 |
|  | #3 | #1 OR #2 | 377580 |
| Intervention/  Comparator | #4 | MH ("Exercise+" OR "Therapeutic Exercise+") | 155695 |
|  | #5 | (TI ( (exercise* OR ‘kinesiotherapy’ OR strength* OR isometric* OR isokinetic* OR aerobic* OR endurance OR weigh* OR resistance OR high intens* OR low intens* OR low impact OR high impact OR dose* OR walk* OR treadmill* OR cycl* OR gait) ) AND AB ( (exercise* OR ‘kinesiotherapy’ OR strength* OR isometric* OR isokinetic* OR aerobic* OR endurance OR weigh* OR resistance OR high intens* OR low intens* OR low impact OR high impact OR dose* OR walk* OR treadmill* OR cycl* OR gait)) AND (TI ( train* OR exercise ) OR AB ( train* OR exercise ))) | 54735 |
|  | #6 | #4 OR #5 | 175179 |
|  | #7 | (TI ( "Systematic Review" OR “Meta Analysis” OR “meta-analys*” ) OR AB ( "Systematic Review" OR “Meta Analysis” OR “meta-analys*”) OR (TI (Review))) | 335829 |
|  | #8 | #3 AND #6 AND #7 | 1330 |
|  | #9 | (MH "Surgery, Operative+") | 754253 |
|  | #10 | #8 NOT #19 | 1191 |
|  | #11 | (MH "Animals+") NOT (MH "Human") | 95874 |
|  | #12 | #10 NOT #11 | 1188 |
| Filters/Limits | #13 | *Filter: English, Academic Journals, Published Date: October 2021 to February 2023* | 160 |

**COCHRANE**

|  | Search | Query | Hits |
| --- | --- | --- | --- |
| Population | #1 | MeSH descriptor: [Pain] explode all trees | 61673 |
|  | #2 | ("pain"):ti,ab,kw | 219075 |
|  | #3 | #1 OR #2 | 226181 |
| Intervention/  Comparator | #4 | MeSH descriptor: [Exercise] explode all trees | 32772 |
|  | #5 | MeSH descriptor: [Exercise Movement Techniques] explode all trees | 2908 |
|  | #6 | MeSH descriptor: [Exercise Therapy] explode all trees | 18436 |
|  | #7 | (exercise* OR ‘kinesiotherapy’ OR strength* OR isometric* OR isokinetic* OR aerobic* OR endurance OR weigh* OR resistance OR train* OR high intens* OR low intens* OR low impact OR high impact OR dose* OR walk* OR treadmill* OR cycl* OR gait):ti,ab,kw | 794800 |
|  | #8 | #4 OR #5 OR #6 OR #7 | 795531 |
|  | #9 | #3 AND #8 | 99792 |
| Filters/Limits | #10 | *Limits: Cochrane reviews, publication date from Oct 2021 to Feb 2023* | 95 |

# Supplementary file 3. Exclusion criteria as list

**POPULATION**

- NOT adult (≥18 years)
- Healthy/asymptomatic adults
- NOT a musculoskeletal pain condition
  - EXCLUDED = pregnancy, labour or post-partum related pain
  - EXCLUDED = post-operative pain if the study is clearly about "immediate post-operative pain", which is iatrogenic pain
  - EXCLUDED = non-musculoskeletal conditions (neurological dysfunction following cardiovascular accident, cardiovascular, oncology conditions)
  - EXCLUDED = post-surgical pain conditions including iatrogenic pain and phantom limb pain, visceral pain (Crohn’s disease and other gastrointestinal disorders, angina, vascular insufficiency, asthma or other breathing disorders), intermittent claudication

| Included conditions | Excluded conditions |
| --- | --- |
| Neck pain- cervical strain, discogenic pain, myofascial pain, radiculopathy, whiplash associated disorder, temporomandibular joint pain | Myelopathy, thoracic outlet syndrome, visceral pain, cancer pain, headache, orofacial pain, wry neck/torticollis |
| Shoulder pain- rotator cuff tendinitis/tendinopathy, adhesive capsulitis, shoulder joint osteoarthritis, acromioclavicular joint pain, bursitis, upper trapezius myalgia, biceps brachii tendinopathy, subacromial impingement syndrome | Rotator cuff rupture, acromioclavicular joint subluxation, shoulder dislocation, instability, labral tear (partial or complete) |
| Elbow pain- nerve pain, tendinopathy, ligament sprain (grade I and II sprain/strain) | Post-traumatic, ligament/muscle/tendon rupture (grade III strain/sprain > 50% disruption in cross sectional area) |
| Wrist pain- carpal tunnel syndrome, triangular fibrocartilage complex sprain, rheumatoid arthritis, tendinitis, osteoarthritis, de Quervain’s tenosynovitis, ganglion cyst | Post-traumatic, compartment syndrome, fracture, carpal instability, joint subluxation, ligament tears (grade III strain/sprain > 50% disruption in the cross sectional area) |
| Hip pain- bursitis or tendonitis/tendinopathy, , muscle strain, piriformis syndrome, rheumatoid arthritis, sacroiliac dysfunction, and referred pain as a result of an L2–3 radiculopathy, greater trochanteric pain syndrome (painful lateral hip) | Post-traumatic, tumor, infection, chondral damage or loose bodies, osteonecrosis of the femoral head, cauda equine syndrome, neoplasm, Paget’s disease, septic hip arthritis, psoriatic arthritis, femoral neck or pubic ramus fracture, hip replacement |
| Knee pain- Patellofemoral pain syndrome, chondromalacia Patellae, Osgood-Schlatter’s disease, Sinding Larsen Johansson syndrome, plica synovialis syndrome, Knee bursitis/Hoffa’s disease, Articular cartilage injury, patellar tendinitis/tendinopathy, patellofemoral osteoarthritis, Pes anserine bursitis, quadriceps tendinopathy, prepatellar bursitis, Iliotibial band syndrome, tendinopathy, ligament strain, muscle strains (grade I and II) | Malignancy, patellofemoral instability/subluxation, fracture, ligament rupture, muscle rupture, osteochondritis dissecans, knee replacement, grade III sprain/strain |
| Ankle pain- Peroneal tendinopathy, nerve impingement, ligament sprain, muscle strain, synovitis, Achilles tendinopathy, plantar fasciitis | Peroneal subluxation, fracture, osteoid osteoma, neuroma, lipoma, hemangioma, osteochondral lesion, giant cell tumor |
| Low back pain- Back strain, disc herniation/degeneration, non-specific back pain, osteoarthritis, sciatica, spinal stenosis, spondylolisthesis, failed back surgery syndrome, radiculopathies | Ankylosing spondylitis, kyphosis, scoliosis, infection, malignancy, visceral pain or pain associated with disease conditions like Crohn’s disease, chronic obstructive pulmonary disorder, myocardial infarction or angina, Gastro-intestinal pain, intermittent claudication. |
| Thoracic pain- Degenerative disc disease | Traumatic and spontaneous vertebral fractures, vascular malformations, infections, spinal or meningeal tumor and metastases, congenital connective tissue or skeletal disorders, compression fracture, scoliosis, kyphosis |
| Fibromyalgia, complex regional pain syndrome | Surgical procedure/accident/burns |
| Rheumatoid arthritis | pain that is caused by the cancer treatment (Radiotherapy, chemotherapy, surgical) |
| Tendinopathies (in general) | Healthy and asymptomatic adults |
| Neuropathic pain | Psoriatic arthritis, reactive arthritis, juvenile arthritis |

**INTERVENTION**

- IS NOT exercise only

| Type of exercise | Inclusion criteria | Exclusion criteria |
| --- | --- | --- |
| Aerobic | Swimming, cycling, elliptical trainer, walking, rowing, upper/lower body ergometer, jogging, running |  |
| Aquatic | Endurance, flexibility, strength, resistance, or aerobic exercise conducted in a pool | Bath or turbulent spa therapy and balneotherapy |
| Flexibility | Ballistic str., dynamic str., active static str., PNF stretching |  |
| Resistance | Isometric, concentric, eccentric, isokinetic (maximal strength training, speed-power training or strength endurance training using elastic resistance bands, free weight, machine or bodyweight) |  |
| Balance | Static/dynamic postural stabilization exercises using unstable surfaces (wobble board, titling platform, mini-trampoline, instrumented inflated platform, balance mat, balance discs, foam pads and rollers, balance boards, stability balls, and computerized balance training systems). |  |
| Combination | ‘Combined’ or ‘concurrent’ training programmes—involving multiple types of exercises performed within the same, or separate exercise sessions of a training programme, respectively | Pharmacological interventions, surgical interventions, cancer rehabilitation |
| Motor control exercise | Stabilization exercises, core stability exercises, and sling exercises |  |
| Mind-body therapies | Pilates, yoga, Tai chi, Baduanjin, Qigong |  |

**COMPARATOR**

- IS NOT exercise OR other conservative, non-pharmacological, non-surgical interventions. The other comparator may have one or more of the following treatments:

1. Exercise
2. No treatment
3. Usual care
4. Waitlist control
5. Education
6. Placebo or sham
7. Other conservative, non-surgical, non-pharmacological interventions

**OUTCOME**

- NOT including at least one of the following outcomes:
  - Subjective physical function (disability)
  - Self-reported pain intensity
  - Psychological function- mental health/depression/anxiety
  - Adherence or attrition rates
  - Adverse events.

**STUDY DESIGN**

- NOT systematic review and meta-analysis (e.g. narrative reviews, opinion pieces, expert opinion, umbrella review)
- Must include a meta-analysis
- EXCLUDE if- Meta-analysis and systematic reviews of non-randomized studies only.

**LANGUAGE**

- NOT English language reviews

**ARTICLE**

- NOT full peer-reviewed journal publication (EXCLUDED: conference papers, theses, grey literature)

# Supplementary file 4. List of studies excluded at full-text review with reasons

| Reason | Study | Title |
| --- | --- | --- |
| Population | Amiri 2017 | The effect of different exercise programs on size and function of deep cervical flexor muscles in patients with chronic nonspecific neck pain: a systematic review of randomized controlled trials. |
| Population | Malliaras 2020 | The efficacy of higher versus lower dose exercise in rotator cuff tendinopathy: a systematic review of randomized controlled trials. |
| Population | VanCant 2020 | Quadriceps strengthening with blood flow restriction for the rehabilitation of patients with knee conditions: A systematic review with meta-analysis. |
| Population | Lowry 2017 | Efficacy of workplace interventions for shoulder pain: A systematic review and meta-analysis. |
| Population | Slade 2006 | Trunk-strengthening exercises for chronic low back pain: a systematic review. |
| Population | Buechter 2011 | Climbing for preventing and treating health problems: a systematic review of randomized controlled trials. |
| Population | vandenDolder 2014 | Effectiveness of soft tissue massage and exercise for the treatment of non-specific shoulder pain: a systematic review with meta-analysis. |
| Population | Hauser 2010 | Efficacy of different types of aerobic exercise in fibromyalgia syndrome: a systematic review and meta-analysis of randomised controlled trials. |
| Population | Nitzsche 2021 | The effectiveness of blood-flow restricted resistance training in the musculoskeletal rehabilitation of patients with lower limb disorders: A systematic review and meta-analysis. |
| Population | Gill 2013 | Does exercise reduce pain and improve physical function before hip or knee replacement surgery? A systematic review and meta-analysis of randomized controlled trials. |
| Population | Verbrugghe 2018 | Motion detection supported exercise therapy in musculoskeletal disorders: a systematic review. |
| Population | Bernard 2018 | Cognitive behavior therapy combined with exercise for adults with chronic diseases: Systematic review and meta-analysis. |
| Population | Garcia-Correa 2021 | Aerobic physical exercise for pain intensity, aerobic capacity, and quality of life in patients with chronic pain: a systematic review and meta-analysis. |
| Population | Hart 2019 | The effect of resistance training on health-related quality of life in older adults: Systematic review and meta-analysis. |
| Population | Mazuquin 2021 | Effectiveness of early versus delayed rehabilitation following rotator cuff repair: Systematic review and meta-analyses. |
| Population | Cerqueira 2021 | Blood flow restriction training: to adjust or not adjust the cuff pressure over an intervention period? |
| Population | Meikis 2021 | Effects of pilates training on physiological and psychological health parameters in healthy older adults and in older adults with clinical conditions over 55 years: a meta-analytical review |
| Population | Paraskevopoulos 2022 | Effectiveness of combined program of manual therapy and exercise vs exercise only in patients with rotator cuff-related shoulder pain: a systematic review and meta-analysis. |
| Population | Heng 2022 | Physical exercise improved muscle strength and pain on neck and shoulder in military pilots. |
| Population | Khodadad 2023 | A systematic review and meta-analysis of resistance training on quality of life, depression, muscle strength, and functional exercise capacity in older adults aged 60 years or more. |
| Population | Mapinduzi 2022 | Effectiveness of motor control exercises versus other musculoskeletal therapies in patients with pelvic girdle pain of sacroiliac joint origin: A systematic review with meta-analysis of randomized controlled trials. |
| Population | Fail 2022 | Benefits of aquatic exercise in adults with and without chronic disease-A systematic review with meta-analysis. |
| Population | daSilva 2022 | Dosage of resistance exercises in fibromyalgia: evidence synthesis for a systematic literature review up-date and meta-analysis. |
| Population | Leemans 2022 | It hurts to move! Intervention effects and assessment methods for movement-evoked pain in patients with musculoskeletal pain: a systematic review with meta-analysis. |
| Intervention/Comparator | Page 2014 | Manual therapy and exercise for adhesive capsulitis (frozen shoulder). |
| Intervention/Comparator | Saltychev 2018 | Effectiveness of conservative treatment for patellofemoral pain syndrome: A systematic review and meta-analysis. |
| Intervention/Comparator | Miller 2010 | Manual therapy and exercise for neck pain: a systematic review. |
| Intervention/Comparator | Adamse 2018 | The effectiveness of exercise-based telemedicine on pain, physical activity and quality of life in the treatment of chronic pain: A systematic review. |
| Intervention/Comparator | Luz Junior 2019 | Effectiveness of kinesio taping in patients with chronic nonspecific low back pain: a systematic review with meta-analysis. |
| Intervention/Comparator | LaTouche 2020 | Effect of manual therapy and therapeutic exercise applied to the cervical region on pain and pressure pain sensitivity in patients with temporomandibular disorders: a systematic review and meta-analysis. |
| Intervention/Comparator | Ceballos-Laita 2019 | Effects of non-pharmacological conservative treatment on pain, range of motion and physical function in patients with mild to moderate hip osteoarthritis. A systematic review. |
| Intervention/Comparator | Mendonca 2020 | How strong is the evidence that conservative treatment reduces pain and improves function in individuals with patellar tendinopathy? A systematic review of randomised controlled trials including GRADE recommendations. |
| Intervention/Comparator | Bernard 2021 | Is adding pelvic floor muscle training to an exercise intervention more effective at improving pain in patients with non-specific low back pain? A systematic review of randomized controlled trials. |
| Intervention/Comparator | Frutiger 2021 | Systematic review and meta-analysis suggest strength training and workplace modifications may reduce neck pain in office workers. |
| Intervention/Comparator | Standaert 2011 | Comparative effectiveness of exercise, acupuncture, and spinal manipulation for low back pain. |
| Intervention/Comparator | Rackwitz 2006 | Segmental stabilizing exercises and low back pain. What is the evidence? A systematic review of randomized controlled trials. |
| Intervention/Comparator | Assendelft 2003 | Spinal manipulative therapy for low back pain. A meta-analysis of effectiveness relative to other therapies. |
| Intervention/Comparator | Casey 2020 | Multidisciplinary-based Rehabilitation (MBR) compared with active physical interventions for pain and disability in adults with chronic pain: a systematic review and meta-analysis. |
| Intervention/Comparator | Bernet 2019 | The effects of hip-targeted physical therapy interventions on low back pain: A systematic review and meta-analysis. |
| Intervention/Comparator | Sutton 2016 | Is multimodal care effective for the management of patients with whiplash-associated disorders or neck pain and associated disorders? A systematic review by the Ontario Protocol for Traffic Injury Management (OPTIMa) Collaboration. |
| Intervention/Comparator | Pengel 2002 | Systematic review of conservative interventions for subacute low back pain. |
| Intervention/Comparator | Bleakley 2019 | Rehabilitation exercises reduce reinjury post ankle sprain, but the content and parameters of an optimal exercise program have yet to be established: a systematic review and meta-analysis. |
| Intervention/Comparator | Kent 2010 | Does targeting manual therapy and/or exercise improve patient outcomes in nonspecific low back pain? A systematic review. |
| Intervention/Comparator | Williams 2007 | Effectiveness of workplace rehabilitation interventions in the treatment of work-related low back pain: a systematic review. |
| Intervention/Comparator | Liang 2019 | The effect of exercise on cervical radiculopathy: A systematic review and meta-analysis. |
| Intervention/Comparator | Challoumas 2020 | Comparison of treatments for frozen shoulder: a systematic review and meta-analysis. |
| Intervention/Comparator | Dickerson 2017 | The effectiveness of exercise therapy for temporomandibular dysfunction: a systematic review and meta-analysis. |
| Intervention/Comparator | Peres 2017 | The practice of physical activity and cryotherapy in rheumatoid arthritis: systematic review. |
| Intervention/Comparator | Conlin 2005 | Treatment of whiplash-associated disorders--part I: Non-invasive interventions. |
| Intervention/Comparator | Conn 2008 | Physical activity interventions among adults with arthritis: meta-analysis of outcomes. |
| Intervention/Comparator | Coulter 2019 | Manipulation and mobilization for treating chronic nonspecific neck pain: a systematic review and meta-analysis for an appropriateness panel. |
| Intervention/Comparator | O'Keeffe 2017 | Are group-based and individual physiotherapy exercise programmes equally effective for musculoskeletal conditions? A systematic review and meta-analysis. |
| Intervention/Comparator | Awotidebe 2019 | Does low-level laser therapy provide additional benefits to exercise in patients with shoulder musculoskeletal disorders? a meta-analysis of randomised controlled trials. |
| Intervention/Comparator | Coulter 2018 | Manipulation and mobilization for treating chronic low back pain: a systematic review and meta-analysis. |
| Intervention/Comparator | Ye 2011 | Effects of rehabilitative interventions on pain, function and physical impairments in people with hand osteoarthritis: a systematic review. |
| Intervention/Comparator | Logan 2017 | Systematic review of the effect of taping techniques on patellofemoral pain syndrome. |
| Intervention/Comparator | Klotz 2019 | Physiotherapy management of patients with chronic pelvic pain (CPP): A systematic review. |
| Intervention/Comparator | Gross 2007 | Conservative management of mechanical neck disorders: a systematic review. |
| Intervention/Comparator | Menke 2014 | Do manual therapies help low back pain? A comparative effectiveness meta-analysis. |
| Intervention/Comparator | Zafar 2015 | Therapeutic effects of whole-body vibration training in knee osteoarthritis: a systematic review and meta-analysis. |
| Intervention/Comparator | O'Keeffe 2016 | Comparative effectiveness of conservative interventions for nonspecific chronic spinal pain: physical, behavioral/psychologically informed, or combined? a systematic review andâ meta-analysis. |
| Intervention/Comparator | Vincent 2013 | Systematic review of manual therapies for nonspecific neck pain. |
| Intervention/Comparator | Pitsillides 2021 | The effects of cognitive behavioural therapy delivered by physical therapists in knee osteoarthritis pain: A systematic review and meta-analysis of randomized controlled trials. |
| Intervention/Comparator | Gross 1996 | Conservative management of mechanical neck disorders. A systematic overview and meta-analysis. |
| Intervention/Comparator | Nascimento 2019 | Effectiveness of interventions for non-specific low back pain in older adults. A systematic review and meta-analysis. |
| Intervention/Comparator | Nakandala 2021 | The efficacy of physiotherapy interventions in the treatment of adhesive capsulitis: A systematic review. |
| Intervention/Comparator | D'Sylva 2010 | Manual therapy with or without physical medicine modalities for neck pain: a systematic review. |
| Intervention/Comparator | Rehman 2020 | Osteopathic manual treatment for pain severity, functional improvement, and return to work in patients with chronic pain. |
| Intervention/Comparator | Gross 2000 | Patient education for mechanical neck disorders. |
| Intervention/Comparator | Rossy 1999 | A meta-analysis of fibromyalgia treatment interventions. |
| Intervention/Comparator | Johnson 2017 | Transcutaneous electrical nerve stimulation (TENS) for fibromyalgia in adults. |
| Intervention/Comparator | Kolber 2021 | PEER systematic review of randomized controlled trials: Management of chronic low back pain in primary care. |
| Intervention/Comparator | Parreira 2014 | Current evidence does not support the use of Kinesio Taping in clinical practice: a systematic review. |
| Intervention/Comparator | Dagenais 2010 | NASS Contemporary Concepts in Spine Care: spinal manipulation therapy for acute low back pain. |
| Intervention/Comparator | Macedo 2013 | Physical therapy interventions for degenerative lumbar spinal stenosis: a systematic review. |
| Intervention/Comparator | Ferreira 2019 | Non-pharmacological and non-surgical interventions for knee osteoarthritis: a systematic review and meta-analysis. |
| Intervention/Comparator | Vassao 2021 | Association of photobiomodulation therapy (PBMT) and exercises programs in pain and functional capacity of patients with knee osteoarthritis (KOA): a systematic review of randomized trials. |
| Intervention/Comparator | Huang 2021 | Can acupuncture improve chronic spinal pain? a systematic review and meta-analysis. |
| Intervention/Comparator | Olaussen 2013 | Treating lateral epicondylitis with corticosteroid injections or non-electrotherapeutical physiotherapy: a systematic review. |
| Intervention/Comparator | Yeh 2019 | Low-level laser therapy for fibromyalgia: a systematic review and meta-analysis. |
| Intervention/Comparator | Gazendam 2021 | Comparative efficacy of nonoperative treatments for greater trochanteric pain syndrome: a systematic review and network meta-analysis of randomized controlled trials. |
| Intervention/Comparator | Hauser 2009 | Efficacy of multicomponent treatment in fibromyalgia syndrome: a meta-analysis of randomized controlled clinical trials. |
| Intervention/Comparator | Herrera-Valencia 2020 | Effcacy of manual therapy in temporomandibularjoint disorders and its medium-and long-termeffects on pain and maximum mouth opening:a systematic review and meta-analysis. |
| Intervention/Comparator | Bunting 2021 | Digital interventions for promoting exercise adherence in chronic musculoskeletal pain: a systematic review and meta-analysis. |
| Intervention/Comparator | Mani-Babu 2015 | The effectiveness of extracorporeal shock wave therapy in lower limb tendinopathy: a systematic review. |
| Intervention/Comparator | Dorji 2020 | The effect of ultrasound or phonophoresis as an adjuvant treatment for non-specific neck pain: systematic review of randomised controlled trials. |
| Intervention/Comparator | Martimbianco 2020 | Photobiomodulation with low-level laser therapy for treating Achilles tendinopathy: a systematic review and meta-analysis. |
| Intervention/Comparator | Minkalis 2018 | A systematic review of thrust manipulation combined with one conservative intervention for rotator cuff and related non-surgical shoulder conditions. |
| Intervention/Comparator | Drescher 2008 | Efficacy of postural and neck-stabilization exercises for persons with acute whiplash-associated disorders: a systematic review. |
| Intervention/Comparator | Southerst 2015 | The effectiveness of manual therapy for the management of musculoskeletal disorders of the upper and lower extremities: a systematic review by the Ontario Protocol for Traffic Injury Management (OPTIMa) Collaboration. |
| Intervention/Comparator | Yohannes 2010 | Management of depression in older people with osteoarthritis: A systematic review. |
| Intervention/Comparator | Saragiotto 2020 | The effectiveness of strategies to promote walking in people with musculoskeletal disorders: a systematic review with meta-analysis. |
| Intervention/Comparator | Halliday 2019 | Treatment Effect Sizes of Mechanical Diagnosis and Therapy for pain and disability in patients with low back pain: a systematic review. |
| Intervention/Comparator | Olivier 2018 | Effectiveness of the McKenzie method of mechanical diagnosis and therapy for treating low back pain: literature review with meta-analysis. |
| Intervention/Comparator | Hoit 2020 | Physiotherapy as an initial treatment option for femoroacetabular impingement: a systematic review of the literature and meta-analysis of 5 randomized controlled trials. |
| Intervention/Comparator | Pelland 2004 | Efficacy of strengthening exercises for osteoarthritis (Part I): A meta-analysis. |
| Intervention/Comparator | Collins 2012 | Efficacy of nonsurgical interventions for anterior knee pain. |
| Intervention/Comparator | Trampas 2006 | Exercise and manual therapy for the treatment of impingement syndrome of the shoulder: a systematic review. |
| Intervention/Comparator | Herrera-Valencia 2020 | Efficacy of manual therapy in temporomandibular joint disorders and its medium-and long-term effects on pain and maximum mouth opening: A systematic review and meta-analysis |
| Intervention/Comparator | Kim 2020 | Meta-analysis of the effects of physical modality therapy and exercise therapy on neck and shoulder myofascial pain syndrome |
| Intervention/Comparator | Huang 2020 | Can acupuncture improve chronic spinal pain? a systematic review and meta-analysis |
| Intervention/Comparator | Murillo-Garcia 2018 | Effects of dance on pain in patients with fibromyalgia: A systematic review and meta-analysis |
| Intervention/Comparator | Kemmler 2017 | Effects of Whole-Body Electromyostimulation on low back pain in people with chronic unspecific dorsal pain: a meta-analysis of individual patient data from randomized controlled wb-ems trials |
| Intervention/Comparator | Chen 2021 | Effectiveness of eccentric strengthening in the treatment of lateral elbow tendinopathy: A systematic review with meta-analysis. |
| Intervention/Comparator | Ramazzina 2019 | Groin pain in athletes and non-interventional rehabilitative treatment: a systematic review. |
| Intervention/Comparator | Petersen 2013 | Proprioception interventions to improve cervical position sense in cervical pathology. |
| Intervention/Comparator | Tator 2017 | Musculoskeletal pain relief in sonographers: a systematic review of the effects of therapeutic techniques. |
| Intervention/Comparator | Pengel 2002 | Systematic review of conservative interventions for subacute low back pain. |
| Intervention/Comparator | Gross 2012 | Patient education for neck pain |
| Intervention/Comparator | Yousefi‐Nooraie 2008 | Low level laser therapy for nonspecific lower back pain |
| Intervention/Comparator | Bidonde 2017 | Whole body vibration exercise training for fibromyalgia |
| Intervention/Comparator | Martimbianco 2017 | Neuromuscular electrical stimulation (NMES) for patellofemoral pain syndrome |
| Intervention/Comparator | Page 2013 | Therapeutic ultrasound for carpal tunnel syndrome |
| Intervention/Comparator | Teirlinck 2023 | Effect of exercise therapy in patients with hip osteoarthritis: A systematic review and cumulative meta-analysis |
| Intervention/Comparator | Zhou 2023 | Is exercise rehabilitation an effective adjuvant to clinical treatment for myofascial trigger points? a systematic review and meta-analysis |
| Intervention/Comparator | deRuvo 2022 | The effect of manual therapy plus exercise in patients with lateral ankle sprains: a critically appraised topic with a meta-analysis |
| Intervention/Comparator | Dean 2021 | Exercise therapy with or without other physical therapy interventions versus placebo interventions for osteoarthritis -Systematic review |
| Intervention/Comparator | Wu 2022 | Is high intensity laser therapy more effective than other physical therapy modalities for treating knee osteoarthritis? A systematic review and network meta-analysis |
| Intervention/Comparator | Sabharwal 2022 | Effectiveness of Neuromuscular exercises (NEMEX) in knee osteoarthritis: A Systematic Review with meta-analysis |
| Intervention/Comparator | Lapner 2022 | Nonoperative treatment of lateral epicondylitis: a systematic review and meta-analysis |
| Intervention/Comparator | Gonzalez-Medina 2021 | Effectiveness of global postural re-education in chronic non-specific low back pain: Systematic review and meta-analysis |
| Intervention/Comparator | Cortes-Perez 2021 | Virtual reality-based therapy reduces the disabling impact of fibromyalgia syndrome in women: Systematic review with meta-analysis of randomized controlled trials |
| Intervention/Comparator | Cramer 2021 | Yoga for low back pain: a systematic review and meta-analysis |
| Intervention/Comparator | Ko 2023 | Comparative short-term effectiveness of non-surgical treatments for insertional Achilles tendinopathy: a systematic review and network meta-analysis. |
| Intervention/Comparator | Liu 2022 | Baduanjin improves neck pain and functional movement in middle-aged and elderly people: A systematic review and meta-analysis of randomized controlled trials. |
| Intervention/Comparator | Shafiee 2023 | The effectiveness of rehabilitation interventions on pain and disability for complex regional pain syndrome: a systematic review and meta-analysis. |
| Intervention/Comparator | Hochheim 2022 | The effectiveness of low-dosed outpatient biopsychosocial interventions compared to active physical interventions on pain and disability in adults with nonspecific chronic low back pain: AÂ systematic review with meta-analysis. |
| Intervention/Comparator | Sasaki 2022 | Effect of exercise and/or educational interventions on physical activity and pain in patients with hip/knee osteoarthritis: A systematic review with meta-analysis. |
| Intervention/Comparator | Paraskevopoulos 2022 | The Effectiveness of neuromobilization in patients with cervical radiculopathy: A systematic review with meta-analysis. |
| Intervention/Comparator | Idanez-Robles 2022 | Exercise therapy improves pain and mouth opening in temporomandibular disorders: A systematic review with meta-analysis. |
| Intervention/Comparator | Kazeminia 2023 | The effect of pelvic floor muscle-strengthening exercises on low back pain: a systematic review and meta-analysis on randomized clinical trials. |
| Intervention/Comparator | Naterstad 2022 | Efficacy of low-level laser therapy in patients with lower extremity tendinopathy or plantar fasciitis: systematic review and meta-analysis of randomised controlled trials. |
| Intervention/Comparator | Santos 2022 | Effectiveness of muscle energy technique in patients with nonspecific low back pain: a systematic review with meta-analysis. |
| Intervention/Comparator | Para-Garcia 2022 | Dry needling alone or in combination with exercise therapy versus other interventions for reducing pain and disability in subacromial pain syndrome: a systematic review and meta-analysis. |
| Intervention/Comparator | Hirohama 2022 | Effects of non-face-to-face and noncontact interventions on knee pain and physical activity in older adults with knee osteoarthritis: a systematic review and meta-analysis. |
| Intervention/Comparator | Tatsios 2022 | The effectiveness of spinal, diaphragmatic, and specific stabilization exercise manual therapy and respiratory-related interventions in patients with chronic nonspecific neck pain: systematic review and meta-analysis. |
| Intervention/Comparator | Yang 2022 | Effectiveness of telehealth-based exercise interventions on pain, physical function and quality of life in patients with knee osteoarthritis: A meta-analysis. |
| Intervention/Comparator | Chen 2022 | The effect of foot orthoses for patients with patellofemoral pain syndrome: A systematic review and meta-analysis. |
| Intervention/Comparator | Oliveira 2022 | Mechanical-based therapies may reduce pain and disability in some patients with knee osteoarthritis: A systematic review with meta-analysis. |
| Intervention/Comparator | Paraskevopoulos 2022 | The effectiveness of neuromobilization exercises in carpal tunnel syndrome: Systematic review and meta-analysis. |
| Intervention/Comparator | Grassini 2022 | Virtual reality assisted non-pharmacological treatments in chronic pain management: a systematic review and quantitative meta-analysis. |
| Intervention/Comparator | Rathnayake 2021 | What is the effect of low back pain self-management interventions with exercise components added? A systematic review with meta-analysis. |
| Intervention/Comparator | Bonatesta 2022 | Pain science education plus exercise therapy in chronic nonspecific spinal pain: a systematic review and meta-analyses of randomized clinical trials. |
| Intervention/Comparator | Ahmad 2022 | Effects of low-level and high-intensity laser therapy as adjunctive to rehabilitation exercise on pain, stiffness and function in knee osteoarthritis: a systematic review and meta-analysis. |
| Intervention/Comparator | Clijsen 2022 | Local heat applications as a treatment of physical and functional parameters in acute and chronic musculoskeletal disorders or pain. |
| Intervention/Comparator | Gazendam 2022 | Comparative efficacy of nonoperative treatments for greater trochanteric pain syndrome: a systematic review and network meta-analysis of randomized controlled trials. |
| Intervention/Comparator | Chen 2022 | Effects of laser therapy on chronic low back pain: A systematic review and meta-analysis of randomized controlled trials. |
| Intervention/Comparator | BatistadeAguiar 2022 | Transcutaneous Electrical Stimulation (TENS) parameters in individuals with fibromyalgia: a systematic review with meta-analysis. |
| Intervention/Comparator | Pourahmadi 2019 | Effectiveness of slump stretching on low back pain: A systematic review and meta-analysis. |
| Intervention/Comparator | Li 2021 | Therapeutic Effects of Traditional Chinese Exercises on musculoskeletal pain: a systematic review and meta-analysis. |
| Intervention/Comparator | Babatunde 2019 | Comparative effectiveness of treatment options for plantar heel pain: a systematic review with network meta-analysis. |
| Intervention/Comparator | Hahne 2010 | Conservative management of lumbar disc herniation with associated radiculopathy: a systematic review. |
| Intervention/Comparator | Nüesch 2013 | Comparative efficacy of pharmacological and non-pharmacological interventions in fibromyalgia syndrome: network meta-analysis. |
| Intervention/Comparator | Assendelft 2004 | Spinal manipulative therapy for low back pain. |
| Intervention/Comparator | Satpute 2021 | Efficacy of mobilization with movement (MWM) for shoulder conditions: a systematic review and meta-analysis. |
| Intervention/Comparator | Casimiro 2002 | Therapeutic ultrasound for the treatment of rheumatoid arthritis |
| Intervention/Comparator | Martini 2022 | Pilates for neck pain: A systematic review and meta-analysis of randomised controlled trials. |
| Intervention/Comparator | Bury 2016 | Effectiveness of scapula-focused approaches in patients with rotator cuff related shoulder pain: A systematic review and meta-analysis. |
| Intervention/Comparator | Alayat 2019 | The effectiveness of high intensity laser therapy in the management of spinal disorders: A systematic review and meta-analysis. |
| Intervention/Comparator | Hadizadeh 2021 | The efficacy of intramuscular electrical stimulation in the management of patients with myofascial pain syndrome: a systematic review. |
| Outcomes | Nicolson 2017 | Interventions to increase adherence to therapeutic exercise in older adults with low back pain and/or hip/knee osteoarthritis: a systematic review and meta-analysis. |
| Outcomes | Blomgren 2018 | Effects of deep cervical flexor training on impaired physiological functions associated with chronic neck pain: a systematic review. |
| Outcomes | Belavy 2021 | Pain sensitivity is reduced by exercise training: Evidence from a systematic review and meta-analysis. |
| Outcomes | Oesch 2010 | Effectiveness of exercise on work disability in patients with non-acute non-specific low back pain: Systematic review and meta-analysis of randomised controlled trials. |
| Outcomes | Schaafsma 2010 | Physical conditioning programs for improving work outcomes in workers with back pain. |
| Outcomes | Armijo-Olivo 2016 | Effectiveness of Manual Therapy and Therapeutic Exercise for Temporomandibular Disorders: Systematic Review and Meta-Analysis. |
| Outcomes | Wewege 2021 | Exercise-Induced Hypoalgesia in Healthy Individuals and People With Chronic Musculoskeletal Pain: A Systematic Review and Meta-Analysis. |
| Outcomes | Hall 2020 | Effect of exercise on pain processing and motor output in people with knee osteoarthritis: a systematic review and meta-analysis. |
| Outcomes | Wayne 2014 | What do we really know about the safety of tai chi?: A systematic review of adverse event reports in randomized trials. |
| Outcomes | Martinez-Calderon 2020 | Which Interventions enhance pain self-efficacy in people with chronic musculoskeletal pain? a systematic review with meta-analysis of randomized controlled trials, including over 12 000 participants. |
| Outcomes | Balachandar 2019 | Iliotibial Band Friction Syndrome: A Systematic Review and Meta-analysis to evaluate lower-limb biomechanics and conservative treatment. |
| Outcomes | Casana 2022 | High-Intensity Interval Training (HIIT) on biological and body composition variables in patients with musculoskeletal disorders: a systematic review and meta-analysis |
| Outcomes | Chen 2022 | On the superiority of a combination of aerobic and resistance exercise for fibromyalgia syndrome: A network meta-analysis. |
| Outcomes | Jankaew 2022 | Therapeutic exercises and modalities in athletes with acute hamstring injuries: a systematic review and meta-analysis. |
| Outcomes | Bell 2022 | Does land-based exercise-therapy improve physical activity in people with knee osteoarthritis? A systematic review with meta-analyses. |
| Outcomes | Vasileios 2022 | Managing fibromyalgia with complementary and alternative medical exercise: a systematic review and meta-analysis of clinical trials. |
| Outcomes | AraújoBispo 2021 | The effects of neuromuscular electrical stimulation on strength, pain, and function in individuals with knee osteoarthritis: a systematic review with meta-analysis. |
| Duplicate | Lam 2018 | Effectiveness of the Mckenzie method of mechanical diagnosis and therapy for treating low back pain: literature review with meta-analysis. |
| Duplicate | Leaver 2010 | Conservative interventions provide short-term relief for non-specific neck pain: a systematic review. |
| Duplicate | Rogan 2019 | Effects of hip abductor muscles exercises on pain and function in patients with patellofemoral pain: a systematic review and meta-analysis. |
| Duplicate | Griffin 2017 | General exercise does not improve long-term pain and disability in individuals with whiplash-associated disorders: a systematic review. |
| Duplicate | Kooiker 2014 | Effects of physical therapist-guided quadriceps-strengthening exercises for the treatment of patellofemoral pain syndrome: a systematic review. |
| Duplicate | Miller 2022 | Correction to: Attempting to Separate Placebo Effects from Exercise in Chronic Pain: A Systematic Review and Meta-analysis. |
| Duplicate | Denham-Jones 2022 | A systematic review of the effectiveness of Pilates on pain, disability, physical function, and quality of life in older adults with chronic musculoskeletal conditions. |
| Study Design | Huffer 2017 | Strength training for plantar fasciitis and the intrinsic foot musculature: A systematic review. |
| Study Design | Lim 2018 | Effects of isometric, eccentric, or heavy slow resistance exercises on pain and function in individuals with patellar tendinopathy: A systematic review. |
| Study Design | Littlewood 2015 | Therapeutic exercise for rotator cuff tendinopathy: a systematic review of contextual factors and prescription parameters. |
| Study Design | vanMiddelkoop 2010 | Exercise therapy for chronic nonspecific low-back pain. |
| Study Design | Marik 2017 | Effectiveness of occupational therapy interventions for musculoskeletal shoulder conditions: a systematic review. |
| Study Design | Page 2016 | Manual therapy and exercise for rotator cuff disease. |
| Study Design | McCaskey 2014 | Effects of proprioceptive exercises on pain and function in chronic neck- and low back pain rehabilitation: a systematic literature review. |
| Study Design | NodehiMoghadam 2020 | Exercise therapy may affect scapular position and motion in individuals with scapular dyskinesis: a systematic review of clinical trials. |
| Study Design | Stuber 2014 | Core stability exercises for low back pain in athletes: a systematic review of the literature. |
| Study Design | Turner 2020 | The Role of Resistance Training Dosing on Pain and Physical Function in Individuals With Knee Osteoarthritis: A Systematic Review. |
| Study Design | Bisset 2005 | A systematic review and meta-analysis of clinical trials on physical interventions for lateral epicondylalgia. |
| Study Design | Wells 2014 | The effectiveness of Pilates exercise in people with chronic low back pain: a systematic review. |
| Study Design | Raman 2012 | Effectiveness of different methods of resistance exercises in lateral epicondylosis--a systematic review. |
| Study Design | RaghavaNeelapala 2020 | Hip muscle strengthening for knee osteoarthritis: a systematic review of literature. |
| Study Design | Lemieux 2020 | Comparing the effectiveness of group-based exercise to other non-pharmacological interventions for chronic low back pain: A systematic review. |
| Study Design | Nelson 2015 | Muscle strengthening activities and fibromyalgia: a review of pain and strength outcomes. |
| Study Design | Henchoz 2008 | Exercise and nonspecific low back pain: a literature review. |
| Study Design | Girard 2019 | The effects of qigong on neck pain: A systematic review. |
| Study Design | Posadzki 2011 | Yoga for low back pain: a systematic review of randomized clinical trials. |
| Study Design | Matheve 2017 | The effectiveness of technology-supported exercise therapy for low back pain: a systematic review. |
| Study Design | Sihawong 2011 | Exercise therapy for office workers with nonspecific neck pain: a systematic review. |
| Study Design | Focht 2006 | Effectiveness of exercise interventions in reducing pain symptoms among older adults with knee osteoarthritis: a review. |
| Study Design | Steele 2015 | A review of the clinical value of isolated lumbar extension resistance training for chronic low back pain. |
| Study Design | Lawford 2016 | Does walking improve disability status, function, or quality of life in adults with chronic low back pain? A systematic review. |
| Study Design | Rabello 2020 | Association between clinical and imaging outcomes after therapeutic loading exercise in patients diagnosed with achilles or patellar tendinopathy at short- and long-term follow-up: a systematic review. |
| Study Design | Reijneveld 2017 | Clinical outcomes of a scapular-focused treatment in patients with subacromial pain syndrome: a systematic review. |
| Study Design | vanderGiessen 2012 | The effectiveness of graded activity in patients with non-specific low-back pain: a systematic review. |
| Study Design | Wasielewski 2007 | Does eccentric exercise reduce pain and improve strength in physically active adults with symptomatic lower extremity tendinosis? A systematic review. |
| Study Design | vanTulder 2000 | Exercise therapy for low back pain. |
| Study Design | Cazzola 2010 | Which kind of exercise is best in fibromyalgia therapeutic programmes? A practical review. |
| Study Design | Song 2021 | Exercise-induced hypoalgesia and pain reduction following blood flow restriction: A brief review. |
| Study Design | Runhaar 2015 | Identifying potential working mechanisms behind the positive effects of exercise therapy on pain and function in osteoarthritis; a systematic review. |
| Study Design | vanTulder 2000 | Exercise therapy for low back pain: a systematic review within the framework of the cochrane collaboration back review group. |
| Study Design | Button 2015 | The clinical effectiveness of self-care interventions with an exercise component to manage knee conditions: A systematic review. |
| Study Design | Hendrick 2010 | The effectiveness of walking as an intervention for low back pain: a systematic review. |
| Study Design | Hadala 2014 | The effectiveness of lumbar extensor training: local stabilization or dynamic strengthening exercises. A review of literature. |
| Study Design | Gross 2002 | Manual therapy for mechanical neck disorders: a systematic review. |
| Study Design | Bonello 2021 | Does isometric exercise result in exercise induced hypoalgesia in people with local musculoskeletal pain? A systematic review. |
| Study Design | Sarig-Bahat 2003 | Evidence for exercise therapy in mechanical neck disorders. |
| Study Design | Southerst 2016 | Is exercise effective for the management of neck pain and associated disorders or whiplash-associated disorders? A systematic review by the Ontario Protocol for Traffic Injury Management (OPTIMa) Collaboration. |
| Study Design | vanRijn 2010 | Effectiveness of additional supervised exercises compared with conventional treatment alone in patients with acute lateral ankle sprains: systematic review. |
| Study Design | Neuhaus 2021 | A systematic review on conservative treatment options for Osgood-Schlatter disease. |
| Study Design | Tollison 1988 | Pain clinic #9. Physical exercise in the treatment of low back pain. Part I: A review. |
| Study Design | Pitsillides 2021 | Blood flow restriction training in patients with knee osteoarthritis: Systematic review of randomized controlled trials. |
| Study Design | Kingma 2007 | Eccentric overload training in patients with chronic Achilles tendinopathy: a systematic review. |
| Study Design | McNeely 2003 | A systematic review of physiotherapy for spondylolysis and spondylolisthesis. |
| Study Design | Kus 2019 | Strengthening the quadriceps femoris muscle versus other knee training programs for the treatment of knee osteoarthritis. |
| Study Design | Cullinane 2014 | Is eccentric exercise an effective treatment for lateral epicondylitis? A systematic review. |
| Study Design | Young 2018 | The influence of dosing on effect size of exercise therapy for musculoskeletal foot and ankle disorders: a systematic review. |
| Study Design | Young 2018 | The influence of exercise dosing on outcomes in patients with knee disorders: a systematic review. |
| Study Design | Dischiavi 2021 | Do exercises for patellofemoral pain reflect common injury mechanisms? A systematic review. |
| Study Design | Hammond 2016 | The effectiveness of home hand exercise programmes in rheumatoid arthritis: a systematic review. |
| Study Design | Kooiker 2014 | Effects of physical therapist-guided quadriceps-strengthening exercises for the treatment of patellofemoral pain syndrome: a systematic review. |
| Study Design | Latif-Zade 2021 | Systematic review shows tele-rehabilitation might achieve comparable results to office-based rehabilitation for decreasing pain in patients with knee osteoarthritis. |
| Study Design | Haik 2016 | Effectiveness of physical therapy treatment of clearly defined subacromial pain: a systematic review of randomised controlled trials. |
| Study Design | Thomson 2016 | The outcome of hip exercise in patellofemoral pain: A systematic review. |
| Study Design | Ortega-Castillo 2016 | Effectiveness of the eccentric exercise therapy in physically active adults with symptomatic shoulder impingement or lateral epicondylar tendinopathy: A systematic review. |
| Study Design | Feger 2015 | Supervised rehabilitation versus home exercise in the treatment of acute ankle sprains: a systematic review. |
| Study Design | Andrade 2018 | A systematic review of the effects of strength training in patients with fibromyalgia: clinical outcomes and design considerations. |
| Study Design | McNeely 2006 | A systematic review of the effectiveness of physical therapy interventions for temporomandibular disorders. |
| Study Design | Lange 2008 | Strength training for treatment of osteoarthritis of the knee: a systematic review. |
| Study Design | Bergstra 2014 | A systematic review into the effectiveness of hand exercise therapy in the treatment of rheumatoid arthritis. |
| Study Design | Romeo 2013 | Manual therapy and therapeutic exercise in the treatment of osteoarthritis of the hip: a systematic review. |
| Study Design | Maquet 2007 | Benefits of physical training in fibromyalgia and related syndromes. |
| Study Design | Juhn 1999 | Patellofemoral pain syndrome: a review and guidelines for treatment. |
| Study Design | Ramazzina 2019 | Groin pain in athletes and non-interventional rehabilitative treatment: a systematic review. |
| Study Design | vanBaar 1999 | Effectiveness of exercise therapy in patients with osteoarthritis of the hip or knee: a systematic review of randomized clinical trials. |
| Study Design | Nelson 2016 | Kinesio taping for chronic low back pain: A systematic review. |
| Study Design | Lee 2008 | Tai chi for osteoarthritis: a systematic review. |
| Study Design | Maly 2014 | Osteoarthritis year in review 2014: rehabilitation and outcomes. |
| Study Design | Clare 2004 | A systematic review of efficacy of McKenzie therapy for spinal pain. |
| Study Design | McNair 2009 | Exercise therapy for the management of osteoarthritis of the hip joint: a systematic review. |
| Study Design | VanderDoelen 2020 | Non-surgical treatment of patellar tendinopathy: A systematic review of randomized controlled trials. |
| Study Design | Scharrer 2012 | A systematic review on the effectiveness of medical training therapy for subacute and chronic low back pain. |
| Study Design | Lee 2009 | Internal qigong for pain conditions: a systematic review. |
| Study Design | Ahern 2020 | The effectiveness of virtual reality in patients with spinal pain: a systematic review and meta-analysis. |
| Study Design | Tsokanos 2021 | The efficacy of manual therapy in patients with knee osteoarthritis: a systematic review. |
| Study Design | Bordado 2019 | Psychosocial effects of workplace exercise - A systematic review. |
| Study Design | Ballestero-Perez 2017 | Effectiveness of Nerve Gliding Exercises on Carpal Tunnel Syndrome: A Systematic Review. |
| Study Design | Santos 2015 | Effectiveness of hip muscle strengthening in patellofemoral pain syndrome patients: a systematic review. |
| Study Design | Kromer 2009 | Effects of physiotherapy in patients with shoulder impingement syndrome: a systematic review of the literature. |
| Study Design | Pisters 2007 | Long-term effectiveness of exercise therapy in patients with osteoarthritis of the hip or knee: a systematic review. |
| Study Design | Valdes 2010 | A systematic review of conservative interventions for osteoarthritis of the hand. |
| Study Design | Siegel 2017 | Effectiveness of occupational therapy interventions for adults with rheumatoid arthritis: a systematic review. |
| Study Design | Ellenbecker 2010 | Rehabilitation of shoulder impingement syndrome and rotator cuff injuries: an evidence-based review. |
| Study Design | Patti 2015 | Effects of Pilates exercise programs in people with chronic low back pain: a systematic review. |
| Study Design | VanHoof 2018 | The efficacy of interventions for low back pain in nurses: A systematic review. |
| Study Design | Wiegerinck 2013 | Treatment for insertional Achilles tendinopathy: a systematic review. |
| Study Design | Sim 2002 | Systematic review of randomized controlled trials of non-pharmacological interventions for fibromyalgia. |
| Study Design | Collins 2012 | Efficacy of nonsurgical interventions for anterior knee pain: systematic review and meta-analysis of randomized trials. |
| Study Design | Green 2003 | Physiotherapy interventions for shoulder pain. |
| Study Design | Axon 2019 | Use of multidomain management strategies by community dwelling adults with chronic pain: evidence from a systematic review. |
| Study Design | Huisman 2013 | The effect of thoracic spine manipulation on pain and disability in patients with non-specific neck pain: a systematic review. |
| Study Design | vonderHeyde 2011 | Occupational therapy interventions for shoulder conditions: a systematic review. |
| Study Design | Zronek 2016 | The influence of home exercise programs for patients with non-specific or specific neck pain: a systematic review of the literature. |
| Study Design | Byrnes 2018 | Is Pilates an effective rehabilitation tool? A systematic review. |
| Study Design | Damgaard 2013 | Evidence of physiotherapy interventions for patients with chronic neck pain: a systematic review of randomised controlled trials. |
| Study Design | Cheng 2015 | Long-term effects of therapeutic exercise on nonspecific chronic neck pain: a literature review. |
| Study Design | Rodrigues 2014 | Effects of exercise on pain of musculoskeletal disorders: a systematic review. |
| Study Design | Raposo 2021 | Effects of exercise on knee osteoarthritis: A systematic review. |
| Study Design | Harvie 2011 | A systematic review of randomized controlled trials on exercise parameters in the treatment of patellofemoral pain: what works? |
| Study Design | Gordon 2016 | A systematic review of the effects of exercise and physical activity on non-specific chronic low back pain. |
| Study Design | Alba-Martin 2015 | Effectiveness of therapeutic physical exercise in the treatment of patellofemoral pain syndrome: a systematic review. |
| Study Design | Vilarino 2021 | Effects of resistance training on the mental health of patients with fibromyalgia: a systematic review. |
| Study Design | Cheung 2016 | Effects of yoga on symptoms, physical function, and psychosocial outcomes in adults with osteoarthritis: a focused review. |
| Study Design | Frye 2012 | The effects of exercise on decreasing pain and increasing function in patients with patellofemoral pain syndrome: a systematic review. |
| Study Design | Machotka 2009 | A systematic review of the literature on the effectiveness of exercise therapy for groin pain in athletes. |
| Study Design | Gobbo 2019 | Physical Exercise Is Confirmed to Reduce Low Back Pain Symptoms in Office Workers: A Systematic Review of the Evidence to Improve Best Practices in the Workplace. |
| Study Design | Ishak 2016 | Effectiveness of Strengthening Exercises for the Elderly with Low Back Pain to Improve Symptoms and Functions: A Systematic Review. |
| Study Design | Quintrec 2014 | Physical exercise and weight loss for hip and knee osteoarthritis in very old patients: a systematic review of the literature. |
| Study Design | Barton 2014 | Patellar taping for patellofemoral pain: a systematic review and meta-analysis to evaluate clinical outcomes and biomechanical mechanisms. |
| Study Design | Ladurner 2021 | Treatment of gluteal tendinopathy: a systematic review and stage-adjusted treatment recommendation. |
| Study Design | Peters 2013 | Proximal exercises are effective in treating patellofemoral pain syndrome: a systematic review. |
| Study Design | Schroeder 2013 | The outcomes of manipulation or mobilization therapy compared with physical therapy or exercise for neck pain: a systematic review. |
| Study Design | Alhakami 2019 | Effects of McKenzie and stabilization exercises in reducing pain intensity and functional disability in individuals with nonspecific chronic low back pain: a systematic review. |
| Study Design | Brumitt 2013 | Core stabilization exercise prescription, part 2: a systematic review of motor control and general (global) exercise rehabilitation approaches for patients with low back pain. |
| Study Design | Posadzki 2011 | Is yoga effective for pain? A systematic review of randomized clinical trials. |
| Study Design | Lin 2016 | Effects of pilates on patients with chronic non-specific low back pain: a systematic review. |
| Study Design | Lee 2014 | Effectiveness of sling exercise for chronic low back pain: a systematic review. |
| Study Design | Seo 2020 | Is scapular stabilization exercise effective for managing nonspecific chronic neck pain?: a systematic review. |
| Study Design | Chang 2016 | Yoga as a treatment for chronic low back pain: A systematic review of the literature. |
| Study Design | Furlan 2009 | Massage for low back pain: an updated systematic review within the framework of the Cochrane Back Review Group. |
| Study Design | Cleland 2002 | The role of therapeutic exercise in treating instability-related lumbar spine pain: A systematic review. |
| Study Design | Hernando-Garijo 2021 | Effectiveness of non-pharmacological conservative therapies in adults with fibromyalgia: A systematic review of high-quality clinical trials. |
| Study Design | Uzawa 2018 | Outcome measures for adherence to home exercises among patients with chronic low back pain: a systematic review. |
| Study Design | Slater 2016 | The influence of exercise on perceived pain and disability in patients with lumbar spinal stenosis: a systematic review of randomized controlled trials. |
| Study Design | Ravichandran 2020 | Effect of scapular stabilization exercise program in patients with subacromial impingement syndrome: a systematic review. |
| Study Design | Head 2019 | The efficacy of loading programmes for improving patient-reported outcomes in chronic midportion Achilles tendinopathy: A systematic review. |
| Study Design | Magnussen 2009 | Nonoperative treatment of midportion Achilles tendinopathy: a systematic review. |
| Study Design | Michener 2004 | Effectiveness of rehabilitation for patients with subacromial impingement syndrome: a systematic review. |
| Study Design | Faber 2006 | Treatment of impingement syndrome: a systematic review of the effects on functional limitations and return to work. |
| Study Design | Beasley 2019 | Conservative therapeutic interventions for osteoarthritic finger joints: A systematic review. |
| Study Design | Collado-Mateo 2015 | Effects of Whole-Body Vibration Therapy in Patients with Fibromyalgia: A Systematic Literature Review. |
| Study Design | Hall 2021 | Rotator cuff related shoulder pain. Describing home exercise adherence and the use of behavior change interventions to promote home exercise adherence: a systematic review of randomized controlled trials. |
| Study Design | deZoete 2020 | The effectiveness of general physical exercise for individuals with chronic neck pain: a systematic review of randomised controlled trials. |
| Study Design | Young 2018 | The influence of exercise dosing on outcomes in patients with knee disorders: a systematic review. |
| Study Design | Mirando 2019 | Is low load blood flow restriction training an effective intervention in improving clinical outcomes in adults with lower extremity pathology: a systematic review. |
| Study Design | Habets 2015 | Eccentric exercise training in chronic mid-portion Achilles tendinopathy: A systematic review on different protocols. |
| Study Design | Merepeza 2014 | Effects of spinal manipulation versus therapeutic exercise on adults with chronic low back pain: a literature review. |
| Study Design | Smith 2014 | A systematic review to determine the optimal type and dosage of land-based exercises for treating knee osteoarthritis. |
| Study Design | Matheve 2017 | The effectiveness of technology-supported exercise therapy for low back pain. |
| Study Design | Abdulla 2015 | Is exercise effective for the management of subacromial impingement syndrome and other soft tissue injuries of the shoulder? A systematic review by the Ontario Protocol for Traffic Injury Management (OPTIMa) Collaboration. |
| Study Design | Woitzik 2015 | The effectiveness of exercise on recovery and clinical outcomes of soft tissue injuries of the leg, ankle, and foot: A systematic review by the Ontario Protocol for Traffic Injury Management (OPTIMa) Collaboration. |
| Study Design | Otoo 2015 | The comparative effectiveness of advice/education compared to active physiotherapy (manual therapy and exercise) in the management of chronic non-specific low back pain. |
| Study Design | Hidalgo 2014 | The efficacy of manual therapy and exercise for different stages of non-specific low back pain: an update of systematic reviews. |
| Study Design | Fersum 2010 | Integration of sub-classification strategies in randomised controlled clinical trials evaluating manual therapy treatment and exercise therapy for non-specific chronic low back pain: a systematic review. |
| Study Design | Hettinga 2007 | A systematic review and synthesis of higher quality evidence of the effectiveness of exercise interventions for non-specific low back pain of at least 6 weeks' duration. |
| Study Design | Lewis 2008 | Are physiotherapy exercises effective in reducing chronic low back pain? [corrected] [published erratum appears in PHYS THER REV 2009 Dec;14(6):422-8]. |
| Study Design | Lewis 2008 | Are physiotherapy exercises effective in reducing chronic low back pain? |
| Study Design | Hauggaard 2007 | Specific spinal stabilisation exercises in patients with low back pain -- a systematic review. |
| Study Design | Hardwick 2012 | Outcomes of strengthening approaches in the treatment of low-grade spondylolisthesis. |
| Study Design | Rajadurai 2009 | Spinal manipulative therapy for low back pain: a systematic review. |
| Study Design | Nyberg 2010 | Limited scientific evidence supports the use of conservative treatment interventions for pain and function in patients with subacromial impingement syndrome: randomized control trials. |
| Study Design | Crowley 2009 | The effectiveness of home exercise programmes for patients with rheumatoid arthritis: a review of the literature. |
| Study Design | Beydagi 2021 | Is therapeutic exercise clinically effective in reducing pain intensity in patients with knee osteoarthritis? a systematic review |
| Study Design | Corkery 2021 | Power training in older adults with knee osteoarthritis |
| Study Design | Matsi 2020 | Effectiveness of craniocervical flexion exercise on pain, disability, and cervical range of motion in patients with neck pain |
| Study Design | vanKoppen 2020 | Adherence to home-based exercises and/or activity advice in low back pain patients: a systematic review |
| Study Design | Jackson 2019 | Physical functioning and mindfulness skills training in chronic pain: A systematic review |
| Study Design | Visvanathan 2018 | Efficacy of endurance exercise on pain and disability in chronic neck pain-a systematic review |
| Study Design | Wang 2014 | Effect of isokinetic training on shoulder impingement |
| Study Design | Ryan 2012 | Physical therapy management of older adults with chronic low back pain: A systematic review |
| Study Design | Dawson 2005 | Physical activity in the treatment and management of fibromyalgia |
| Study Design | Beydag 2021 | Is therapeutic exercise clinically effective in reducing pain intensity in patients with knee osteoarthritis? |
| Study Design | Zacharakis 2020 | What is the evidence for the effectiveness of scapula-thoracic strengthening exercises in individuals with neck pain: a systematic review. |
| Study Design | Gardiner 2020 | Are stabilisation exercises different to other treatments in improving physical activity or reducing disability for people with persistent low back pain? A systematic literature review. |
| Study Design | Jasmin 2019 | Effectiveness of Yoga Intervention for Chronic Neck Pain: A Systematic Literature Review. |
| Study Design | Elliott 2018 | Systematic review of the addition of hip strengthening exercises for adults with patellofemoral pain syndrome. |
| Study Design | Shamsi 2020 | Effectiveness of physiotherapy management in knee osteoarthritis: A systematic review. |
| Study Design | Cruz 2019 | Exercise-based interventions for physically active individuals with functional ankle instability: a systematic review of the literature. |
| Study Design | Menta 2015 | The Effectiveness of Exercise for the Management of Musculoskeletal Disorders and Injuries of the Elbow, Forearm, Wrist, and Hand: A Systematic Review by the Ontario Protocol for Traffic Injury Management (OPTIMa) Collaboration. |
| Study Design | FernandaCemin 2017 | Effects of the Pilates method on neck pain: a systematic review. |
| Study Design | Hilde 1998 | Effect of exercise in the treatment of chronic low back pain: a systematic review, emphasising type and dose of exercise. |
| Study Design | Iversen 2012 | Rehabilitation Interventions for pain and disability in osteoarthritis: A review of interventions including exercise, manual techniques, and assistive devices...Reprinted with permission from American Journal of Nursing, 112(3), S32-S37, 2012 |
| Study Design | Oldfield 2008 | Exercise therapy and orthotic devices in rheumatoid arthritis: evidence-based review. |
| Study Design | Posadzki 2011 | Pilates for low back pain: A systematic review. |
| Study Design | Park 2012 | Nonpharmacological approaches to the management of chronic pain in community-dwelling older adults: a review of empirical evidence. |
| Study Design | Morone 2007 | Mind-body interventions for chronic pain in older adults: a structured review. |
| Study Design | Goldenberg 2004 | Management of fibromyalgia syndrome. |
| Study Design | Koes 1991 | Physiotherapy exercises and back pain: a blinded review. |
| Study Design | McCarthy 1999 | The effectiveness of exercise in the treatment of osteoarthritic knees: a critical review. |
| Study Design | Marks 1993 | Quadriceps strength training for osteoarthritis of the knee: a literature review and analysis. |
| Study Design | French 2008 | Gluteal muscle dysfunction and the role of specific strengthening in hip osteoarthritis: a review. |
| Study Design | Protas 1996 | Aerobic exercise in the rehabilitation of individuals with chronic low back pain: a review. |
| Study Design | Surace 2020 | Shock wave therapy for rotator cuff disease with or without calcification |
| Study Design | Page 2012 | Exercise and mobilisation interventions for carpal tunnel syndrome |
| Study Design | O'Connor 2003 | Non-surgical treatment (other than steroid injection) for carpal tunnel syndrome |
| Study Design | Nwodo 2022 | Review of core stability exercise versus conventional exercise in the management of chronic low back pain |
| Study Design | McHugh 2022 | Effectiveness of remote exercise programs in reducing pain for patients with knee osteoarthritis: A systematic review of randomized trials |
| Study Design | Romao 2022 | The effect of pilates exercises on muscle electrical activation in adults with chronic low back pain: a systematic review |
| Study Design | daSilvaJunior 2022 | Effect of physical training in obese patients with low back pain: a systematic review |
| Study Design | Wang 2022 | The effect and mechanism of traditional Chinese exercise for chronic low back pain in middle-aged and elderly patients: A systematic review |
| Study Design | Balchin 2022 | Acute effects of exercise on pain symptoms, clinical inflammatory markers and inflammatory cytokines in people with rheumatoid arthritis: a systematic literature review |
| Study Design | Duenas 2021 | Specific versus non-specific exercises for chronic neck or shoulder pain: A systematic review |
| Study Design | Paraskevopoulos 2023 | A Systematic Review of the Aerobic Exercise Program Variables for Patients with Non-Specific Neck Pain: Effectiveness and Clinical Applications. |
| Study Design | Bounds 2023 | Efficacy of Conservative Interventions for Musculoskeletal Conditions on Pain and Disability in Active Serving Military Personnel-A Systematic Review. |
| Study Design | Singh 2022 | Subacromial Impingement Syndrome: A Systematic Review of Existing Treatment Modalities to Newer Proprioceptive-Based Strategies. |
| Study Design | Smrcina 2022 | A Systematic Review of the Effectiveness of Core Stability Exercises in Patients with Non-Specific Low Back Pain. |
| Study Design | Klaps 2022 | The Influence of Exercise Intensity on Psychosocial Outcomes in Musculoskeletal Disorders: A Systematic Review. |
| Study Design | Gava 2022 | Effectiveness of physical therapy given by telerehabilitation on pain and disability of individuals with shoulder pain: A systematic review. |
| Study Design | Tan 2022 | Does aerobic exercise effect pain sensitisation in individuals with musculoskeletal pain? A systematic review. |
| Study Design | Ammendolia 2022 | Non-operative treatment for lumbar spinal stenosis with neurogenic claudication: an updated systematic review. |
| Study Design | Pieri 2022 | Well-described exercises for chronic low back pain in Life Science Literature: A systematic review. |
| Study Design | Nunez-Martinez 2022 | Management of Patellar Tendinopathy Through Monitoring, Load Control, and Therapeutic Exercise: A Systematic Review. |
| Study Design | daSilvaJunior 2022 | Association of photobiomodulation therapy and therapeutic exercises in relation to pain intensity and neck disability in individuals with chronic neck pain: a systematic review of randomized trials. |
| Study Design | Tan 2021 | The Relationship Between Changes in Movement and Activity Limitation or Pain in People With Knee Osteoarthritis: A Systematic Review. |
| Study Design | Taul-Madsen 2022 | Exercise booster sessions as a mean to maintain the effect of an exercise-intervention - A systematic review. |
| Study Design | Sutanto 2021 | Effectiveness Of Different Trunk Muscle Training Methods For Non-specific Low Back Pain: A Meta-analysis. |
| Study Design | YajingZhao 2021 | Efficacy Of Exercise Combined With Conventional Therapy On CLBP Patients: A Network Meta-analysis. |
| Study Design | Mendonca 2019 | The evidence for conservative treatment in reducing pain and improving function in patellar tendinopathy is of low quality: a systematic review of randomised controlled trials including GRADE recommendations. |
| Study Design |  | The effectiveness of hip strengthening exercises in the management of patellofemoral pain syndrome (PFPS) in females: a systematic review. |
| Study Design |  | Effectiveness and optimal dosage of resistance training for chronic neck pain: a systematic review with a qualitative synthesis and meta-analysis. |
| Study Design |  | Comparative effectiveness of exercise or self-management of plantar heel pain: a systematic review and meta-analysis. |
| Study Design |  | The adjunctive benefit of manual therapy in addition to therapeutic exercise for subacromial pain syndrome: a systematic review. |
| Study Design |  | Exercise Effects On Depressive and Anxiety Symptoms, Fatigue And Pain in Rheumatoid Arthritis: A Meta-Analysis: 1953 Board #214 May 31 3:30 PM - 5:00 PM. |
| Study Design |  | Comparative effectiveness of treatment options for subacromial shoulder pain: a network meta-analysis. |
| Study Design |  | Should exercises be painful? A systematic review and meta-analysis. |
| Study Design | Thompson 2015 | Are core stability exercises an effective treatment for nonspecific chronic low back pain? A systematic review with meta-analysis. |
| Study Design | Jess 2016 | The effectiveness of matched subgrouping for manual therapy, exercise therapy and psychosocial interventions for non-specific low back pain. A systematic review. |
| Study Design | Mayer 2011 | Lumbar Extensor Strengthening Exercise for Chronic Low Back Pain: A Systematic Review. |
| Study Design |  | Systematic review of exercise and chronic low back pain |
| Study Design | vanLinschoten 2011 | Exercise Therapy For Patellofemoral Pain Syndrome, A Systematic Review. |
| Study Design | Heiss 2003 | Exercise prescription and the back. (Review) |
| Study Design | Alderink 2002 | Back pain solutions, how to help yourself with posture-movement therapy and education. (Review) |
| Study Design | Bannuru 2020 | Comparative effectiveness of mind-body interventions for knee osteoarthritis: An individual-participant data network metaanalysis |
| Study Design | Leininger 2020 | Cost-effectiveness of spinal manipulation, supervised exercise, or home exercise for spinal pain in the united states using an individual participant data meta-analysis approach |
| Study Design | Anheyer 2019 | Yoga for treating low back pain - a systematic review and meta-analysis |
| Study Design | Romy 2012 | Tai chi for osteoarthritis of the knee-a systematic review and meta-analysis |
| Study Design | Nagel 2010 | Effects of exercise therapy on patients with rheumatoid arthritis-A systematic review |
| Study Design |  | Effectiveness and optimal dosage of resistance training for chronic neck pain: a systematic review with a qualitative synthesis and meta-analysis...Chartered Society of Physiotherapy Conference & Trade Exhibition, Physiotherapy UK 2019, Nov 1, 2019-Nov 2, |
| Study Design | Kedroff 2019 | Do exercise programmes change physical factors in people with patellofemoral pain? A systematic review and meta-analysis...British Society for Rheumatology Annual Conference 2019, April 30-May 02 2019, Birmingham, United Kingdom |
| Study Design |  | Comparative effectiveness of treatment options for subacromial shoulder pain: a network meta-analysis...The Chartered Society of Physiotherapy UK Conference 2018, Birmingham, England, 19-20 October 2018. |
| Study Design | Gibbons 2010 | Specific motor control exercise for lumbo-pelvic pain of articular origin: a systematic review. |
| Study Design |  | Motor control exercise for persistent nonspecific low back pain: a systematic review. |
| Study Design | Charles 2022 | The impact of high intensity resistance training on low back pain disability: a systematic review and meta-analysis |
| Study Design | Neal 2022 | Numerous treatments are efficacious for patellofemoral pain: a systematic review and meta-analysis |
| Study Design | Swinton 2022 | Empirically derived guidelines for interpreting the effectiveness of exercise therapy for tendinopathies: a meta-analysis |
| Study Design | Koch 2022 | Comparative Efficacy of Different Exercise Interventions in Chronic Non-Specific Low Back Pain: A Systematic Review and Network Meta-Analysis |
| Study Design | Fan 2022 | The effectiveness of exercise based digital health interventions (requiring internet) in management of hip and knee osteoarthritis: a systematic review and meta-analysis |
| Study Design | Pedersen 2022 | Comparator groups matter! - the impact of comparator interventions on effect estimates: a systematic review and meta-analysis of randomized controlled trials of exercise for knee osteoarthritis |
| Study Design | Holden 2022 | Moderators of the effect of therapeutic exercise for people with knee and/or hip osteoarthritis: an individual participant data meta-analysis |
| Study Design | Bannuru 2022 | Factors associated with response to exercise interventions in adults with symptomatic knee osteoarthritis - an individual patient data network meta-analysis |
| Study Design | Juhl 2022 | Comparison of exercise therapy, non-steroidal anti-inflammatory drugs, and opioids for knee osteoarthritis pain: a systematic review and network meta-analysis |
| Study Design | Bannuru 2022 | An individual-participant data network meta-analysis assessing the comparative effectiveness of mind-body interventions for knee osteoarthritis |
| Study Design | Bremer 2022 | Management of gluteal tendinopathy: A systematic review and meta-analysis of all interventions |
| Study Design | Tang 2022 | Hydrotherapy reduces pain in short-term but not in long-term in patients with hip/knee osteoarthritis- a systematic review with meta-analyses |
| Study Design | Taylor 2021 | Breathing exercises in the treatment of chronic low back pain to reduce pain perception and stress |
| Study Design | Thornton 2021 | Treating low back pain in athletes: A systematic review with meta-analysis |
| Study Design | Goff 2021 | Patient education for knee osteoarthritis systematic review and meta-analysis |
| Study Design | Broszko 2021 | Is exercise therapy effective treatment for low back pain? |
| Study Design | Golightly 2012 | A comprehensive review of the effectiveness of different exercise programs for patients with osteoarthritis. |
| Study Design | Steiger 2012 | Is a positive clinical outcome after exercise therapy for chronic non-specific low back pain contingent upon a corresponding improvement in the targeted aspect(s) of performance? A systematic review. |
| Study Design | Iwamoto 2010 | Effectiveness of exercise in the treatment of lumbar spinal stenosis, knee osteoarthritis, and osteoporosis. |
| Study Design | Alvarez-Gallardo 2019 | Therapeutic validity of exercise interventions in the management of fibromyalgia. |
| Study Design | Field 2016 | Knee osteoarthritis pain in the elderly can be reduced by massage therapy, yoga and tai chi: A review. |
| Study Design | Jadidi 2020 | A Review of Non-Surgical Pain Management in Osteoarthritis. |
| Study Design | Iversen 2012 | Do Dynamic Strengthening and Aerobic Capacity Exercises Reduce Pain and Improve Functional Outcomes and Strength in People With Established Rheumatoid Arthritis? |
| Study Design | Mujalli 2018 | The effect of a therapeutic program on the degree of cervical herniated disc. |
| Study Design | Murphy 2018 | Rate of Improvement of Pain and Function in Mid-Portion Achilles Tendinopathy with Loading Protocols: A Systematic Review and Longitudinal Meta-Analysis. |
| Study Design | Franco 2017 | Aquatic Exercise for the Treatment of Hip and Knee Osteoarthritis. |
| Study Design | Brosseau 2016 | Ottawa Panel evidence-based clinical practice guidelines for therapeutic exercise in the management of hip osteoarthritis*. |
| Study Design |  | Core stability exercises for low back pain in athletes: a systematic review of the literature. |
| Study Design | Busanich 2006 | Does McKenzie Therapy Improve Outcomes for Back Pain? |
| Study Design | Henschke 2014 | Exercise reduces pain and improves physical function for people awaiting hip replacement surgery. |
| Study Design | Mist 2013 | Complementary and alternative exercise for fibromyalgia: A meta-analysis |
| Study Design | Weissenfels 2019 | Comparison of Whole-Body Electromyostimulation versus Recognized Back-Strengthening Exercise Training on Chronic Nonspecific Low Back Pain: A Randomized Controlled Study. |
| Study Design | Kujala 2009 | Evidence on the effects of exercise therapy in the treatment of chronic disease. |
| Study Design | Speers 2018 | Lateral elbow tendinosis: a review of diagnosis and management in general practice. |
| Study Design | Bennell 2005 | Exercise as a treatment for osteoarthritis. |
| Study Design | Bennell 2014 | Review: exercise interventions improve pain and function in people with knee osteoarthritis compared with no exercise. |
| Study Design | Smith 2008 | Twenty years of specific, isolated lumbar extension research: a review. |
| Study Design | Silva 2010 | Balance training (proprioceptive training) for patients with rheumatoid arthritis |
| Study Design | Heikkinen 2022 | Impact or No Impact for Women With Mild Knee Osteoarthritis: A Bayesian Meta-Analysis of Two Randomized Controlled Trials With Contrasting Interventions. |
| Study Design | Niederer 2022 | What Modifies the Effect of an Exercise Treatment for Chronic Low Back Pain? A Meta-epidemiologic Regression Analysis of Risk of Bias and Comparative Effectiveness. |
| Study Design | Geigle 2022 | Exercise in the Aquatic Environment for People With Primary Hip Osteoarthritis: A Systematic Review and Meta-analyses. |
| Study Design |  | Can Exercise Prevent Low Back Pain? A Systematic Review says â€œYes.â€ But Questions Linger... |
| Study Design | Littlewood 2020 | Can "strong" recommendations be made for exercise and manual therapy in treating subacromial shoulder pain?...Pieters L, Lewis J, Kuppens K, et al. An update of systematic reviews examining the effectiveness of conservative physical therapy interventions |
| Study Design | Abbott 2005 | Exercise therapy is effective for chronic low back pain, but should be individually prescribed and supervised. |
| Study Design | Pieters 2020 | Can "strong" recommendations be made for exercise and manual therapy in treating subacromial shoulder pain?...Littlewood C, May S, Walters S. A review of systematic reviews of the effectiveness of conservative interventions for rotator cuff tendinopathy. |
| Study Design | Tomoyuki 2018 | Exercise Therapy for Low Back Pain A Systematic Review. |
| Study Design | Tomoyuki 2018 | Exercise Therapy for Low Back Pain A Systematic Review...Matheve T, Brumagne S, Timmermans AAA: The effectiveness of technology-supported exercise therapy for low back pain: a systematic review. American Journal of Physical Medicine & Rehabilitation (AM J |
| Study Design | Murphy 2018 | Is heavy eccentric calf training superior to wait-and-see, sham rehabilitation, traditional physiotherapy and other exercise interventions for pain and function in mid-portion Achilles tendinopathy? |
| Study Design | Rosen 2002 | Spinal stabilization: the new science of back pain. (Review) |
| Shortened republication of reviews | Coulombe 2017 | Core Stability Exercise Versus General Exercise for Chronic Low Back Pain. |
| Shortened republication of reviews | Fransen 2015 | Exercise for osteoarthritis of the knee: a Cochrane systematic review. |
| Shortened republication of reviews | Saragiotto 2016 | Motor Control Exercise for Nonspecific Low Back Pain: A Cochrane Review. |
| Shortened republication of reviews | Gross 2016 | Exercises for mechanical neck disorders: A Cochrane review update. |
| Shortened republication of reviews | Yamato 2016 | Pilates for Low Back Pain: Complete Republication of a Cochrane Review. |
| Shortened republication of reviews | Hayden 2005 | Meta-analysis: exercise therapy for nonspecific low back pain. |
| Shortened republication of reviews | Hayden 2005 | Systematic review: strategies for using exercise therapy to improve outcomes in chronic low back pain. |
| Shortened republication of reviews | Busch 2008 | Exercise for fibromyalgia: a systematic review. |
| Shortened republication of reviews | Uthman 2013 | Exercise for lower limb osteoarthritis: systematic review incorporating trial sequential analysis and network meta-analysis. |
| Shortened republication of reviews | Peek 2016 | Different forms of exercise for chronic low back pain (PEDro synthesis). |
| Shortened republication of reviews | Yamato 2015 | Therapeutic exercise for chronic non-specific neck pain: PEDro systematic review update. |
| Shortened republication of reviews | Hart 2006 | Exercise therapy for nonspecific low-back pain: a meta-analysis. |
| Shortened republication of reviews | Goh 2019 | Efficacy and potential determinants of exercise therapy in knee and hip osteoarthritis: A systematic review and meta-analysis. |
| Shortened republication of reviews | Osteras 2017 | Exercise for Hand Osteoarthritis: A Cochrane Systematic Review. |
| Shortened republication of reviews | VanDerHeijden 2016 | Exercise for treating patellofemoral pain syndrome: an abridged version of Cochrane systematic review. |
| Shortened republication of reviews | Saragiotto 2015 | Yoga for low back pain: PEDro systematic review update. |
| Shortened republication of reviews | Uthman 2014 | Exercise for lower limb osteoarthritis: systematic review incorporating trial sequential analysis and network meta-analysis. |
| Shortened republication of reviews | Sauers 2005 | Effectiveness of Rehabilitation for Patients with Subacromial Impingement Syndrome. |
| Shortened republication of reviews | Scarvell 2011 | Aerobic exercise is beneficial for people with rheumatoid arthritis. |
| Shortened republication of reviews | Macintyre 2005 | 5-Year Follow-Up of Open or Closed Kinetic Chain Exercises for Patellofemoral Pain. (Review) |
| Shortened republication of reviews |  | What Factors in Exercise Therapy Improve Outcomes in Chronic Low Back Pain? |
| Shortened republication of reviews | Hagen 2001 | Review: Bed Rest Is Not Effective for Acute Low-Back Pain or Sciatica. |
| Shortened republication of reviews | Schattner 2013 | Review: Strength training, with or without flexibility and aerobic training, reduces pain in lower limb osteoarthritis. |
| Shortened republication of reviews | Ciliska 2003 | Review: aerobic exercise improves cardiovascular fitness and tender points in fibromyalgia. |
| Shortened republication of reviews | Carey 2006 | Review: exercise therapy reduces pain and improves function in chronic but not acute low-back pain. |
| Shortened republication of reviews | Yelland 2009 | Review: Exercise helps relieve pain in patients with hip osteoarthritis. |
| Shortened republication of reviews | Schattner 2005 | Review: both aerobic and home-based quadriceps strengthening exercises reduce pain and disability in knee osteoarthritis. |
| Shortened republication of reviews | Hayden 2021 | Some types of exercise are more effective than others in people with chronic low back pain: a network meta-analysis. |
| Updated at a later date | Ebadi 2014 | Therapeutic ultrasound for chronic low-back pain. |
| Updated at a later date | Macedo 2009 | Motor control exercise for persistent, nonspecific low back pain: a systematic review. |
| Updated at a later date | Fransen 2008 | Exercise for osteoarthritis of the knee. |
| Updated at a later date | Busch 2002 | Exercise for treating fibromyalgia syndrome. |
| Updated at a later date | Kay 2005 | Exercises for mechanical neck disorders. |
| Updated at a later date | Skelly 2018 |  |
| Updated at a later date | Hidalgo 2016 | The efficacy of manual therapy and exercise for treating non-specific neck pain: An update of systematic reviews. |
| Updated at a later date | Kay 2015 | Exercises for mechanical neck disorders. |
| Updated at a later date | Wieland 2017 | Yoga treatment for chronic non-specific low back pain |
| Language | Lorena 2015 | Effects of muscle stretching exercises in the treatment of fibromyalgia: a systematic review. |
| Language | Messerli 2022 | Effectiveness of pelvic floor exercises in the treatment of chronic low back pain - Systematic review |
| Language | LopezMesa 2023 | Pilates . Effects on physical function and its limitations. Systematic review and metaanalysis. |
| Language | IuriFreitas 2022 | Effects of resistant training on scapular muscles in epicondylalgia: systematic review and meta-analysis. |
| Language | BarrosdePaula 2022 | EFICÃCIA DO TREINAMENTO RESISTIDO NO TRATAMENTO DA CONDROMALÃCIA PATELAR: REVISÃƒO SISTEMÃTICA DA LITERATURA. |
| Retracted at a later date | Ferlito 2020 | The blood flow restriction training effect in knee osteoarthritis people: a systematic review and meta-analysis. |
| No full-text available | Hadhazy 2000 | Mind-body therapies for the treatment of fibromyalgia. A systematic review. |
| No full-text available | Horng 2006 | Yoga improves function in patients with chronic low back pain |
| No full-text available | Smith 2008 | Article: What occupational therapy interventions have an effect on hand function in clients with rheumatoid arthritis? A systematic review. |
| No full-text available |  | Effect of Adjuvant Exercise Treatment on Chronic Low Back Pain: A Bayesian Network Meta - analysis. |

# Supplementary file 5. Characteristics of included reviews

| Review (author, year, pain condition) | Musculoskeletal disorders | N | n | Intervention | Comparator | Outcomes | Tool ROB |
| --- | --- | --- | --- | --- | --- | --- | --- |
| Ankle and foot pain | |  |  |  |  |  |  |
| Murphy 2019[1] | Mid-portion Achilles tendinopathy | 6 | 241 | Heavy eccentric calf-training | Exercise (Stanish protocol, slow resistance calf training), Physiotherapy | P, PF | Cochrane Risk of Bias tool |
| Siriphorn 2020[2] | Plantar fasciitis | 8 | 681 | Calf stretching | Plantar fascia specific stretching, shockwave therapy | P | Cochrane Risk of Bias tool |
| Sussmilch-Leitch 2012[3] | Achilles tendinopathy | 2 | NR | Eccentric strengthening exercise | Shockwave therapy | P | PEDro |
| Wilson 2018[4] | Achilles tendinopathy | 5 | 1137 | High intensity eccentric exercise | Lower intensity eccentric exercise | P, PF | Cochrane Risk of Bias tool |
| Arora 2022[5] | Achilles tendinopathy | 8 | 467 | Eccentric exercises | Physical modality and eccentric exercises | P, PF | Cochrane Risk of Bias tool |
| Prudencio 2023[6] | Mid‑portion Achilles tendinopathy | 8 | 371 | Eccentric exercise | Concentric exercise, light training, heavy slow resistance (HSR), another protocol of eccentric exercise, electrotherapy, waitlist control, vibration training | P | Cochrane Risk of Bias tool |
| Chronic widespread pain | |  |  |  |  |  |  |
| Bidonde 2019[7] | Fibromyalgia | 29 | 2088 | Mixed exercise (Aerobic, resistance, flexibility) | Other exercise and other intervention | P, PF, AR | Cochrane Risk of Bias tool |
| Busch 2013[8] | Fibromyalgia | 5 | 241 | Resistance training | Control, aerobic exercise, flexibility exercise | P, PF, MH, AR | Cochrane Risk of Bias tool |
| Cheng 2019[9] | Fibromyalgia | 6 | 657 | Tai chi | Standard care and conventional therapeutic exercise | P, MH | Cochrane Risk of Bias tool |
| Guzman-Pavon 2020[10] | Myofascial pain syndrome | 24 | 1221 | Physical exercise (strength, aerobic, coordination, proprioception, and postural correction exercises) | Control (education, no treatment) | P, PF | Cochrane Risk of Bias tool |
| Busch 2007[11] | Fibromyalgia | 34 | 2276 | Exercise (aerobic, flexibility, strength, mixed) | Multidisciplinary program, mixed exercise | P, PF, MH | Jadad |
| Langhorst 2013[12] | Fibromyalgia | 7 | 362 | Meditative movement therapies (Qigong, Tai Chi and Yoga) | Usual care, exercise, waitlist control | P, MH | NR |
| Mata Diz 2017[13] | Myofascial pain | 8 | 255 | Exercise (stretching, strengthening) | Dry needling, no treatment, shock wave therapy | P | PEDro |
| Sosa-Reina 2017[14] | Fibromyalgia | 14 | 715 | Aerobic, strengthening, stretching, and combined exercise | No treatment, usual care, exercises | P, PF, MH | Cochrane Risk of Bias tool |
| Bidonde 2017[15] | Fibromyalgia | 13 | 839 | Aerobic exercise | Exercise, advice, no treatment | P, PF | Cochrane Risk of Bias tool |
| Galvao-Moreira 2021[16] | Fibromyalgia | 14 | NR | Aquatic exercise | Land-based exercises or no exercise | P | Cochrane Risk of Bias tool |
| Bidonde 2014[17] | Fibromyalgia | 24 | 881 | Land and aquatic mixed interventions (aerobics, flexibility, co-ordination and/or strength) | Control, land-based interventions (physiotherapy, kinesiotherapy), aquatic exercise | P, PF, MH, AR | Cochrane Risk of Bias tool |
| McDowell 2017[18] | Fibromyalgia | 10 | 595 | Exercise (Aerobics, endurance, flexibility, coordination, walking, jogging) | No treatment, waitlist control, education | MH | Chalmers system |
| Kim 2019[19] | Fibromyalgia | 12 | 743 | Flexibility exercise | Aerobic, resistance, Pilates, Tai chi | P, PF, MH | Cochrane Risk of Bias tool |
| Bravo 2019[20] | Fibromyalgia | 16 | 1294 | Body awareness therapy (Yoga, Qigong, Tai Chi, strengthening, Mensendieck system) | Control (usual care, stretching, Qigong, aerobic, education) | PF | Cochrane Risk of Bias tool |
| Zhang 2022 (a) [21] | Fibromyalgia | 57 | 3319 | Stretching, MBE, strengthening, sensorimotor training, land based training | Non-intervention control, active control | P, MH | Cochrane Risk of Bias tool |
| Couto 2022[22] | Fibromyalgia | 18 | 1184 | Exercises (aerobic, resistance, stretching) | Placebo, exercise, manual therapy | P, PF, MH | Cochrane Risk of Bias tool |
| Vilarino 2022[23] | Fibromyalgia | 13 | 839 | Resistance exercises | Flexibility training, relaxation therapy | P, PF | Cochrane Risk of Bias tool |
| Murillo-Garcia 2022[24] | Fibromyalgia | 15 | NR | Aerobic, pool based, video tape based, belly, and thera dance | Stretching, combined, land based, aerobic exercise and no exercise | P, PF | Cochrane Risk of Bias tool |
| Kundakci 2022[25] | Fibromyalgia | 35 | 2013 | Mixed exercise, mind–body, aerobic, strengthening, flexibility | Usual care, waitlist, plcebo or sham treatment | P, PF, MH | Cochrane Risk of Bias tool |
| Albuquerque 2022[26] | Fibromyalgia | 16 | NR | Strength, endurance, aerobic, stretching, aquatic exercises | No intervention | P | Cochrane Risk of Bias tool |
| Wu 2022 (a)[27] | Fibromyalgia | 9 | 466 | Exergame training | Aerobic, conventional therapy, stretching | P, PF, MH | Cochrane Risk of Bias tool |
| Ma 2022[28] | Fibromyalgia | 14 | 762 | Aquatic exercises | No exercise, education, home-based exercise | P, PF, MH | Cochrane Risk of Bias tool |
| Combination |  |  |  |  |  |  |  |
| Barker 2014[29] | Rheumatoid arthritis, OA, fibromyalgia, low back pain | 26 | NR | Aquatic exercise | No exercise or land-based exercise | P, PF | PEDro |
| Brosseau 2004[30] | OA (Hand, knee) | 12 | 1363 | Aerobic exercise (aerobic, walking, functional strengthening) | Control, exercise and education | P, PF | Jadad |
| Collado-Mateo 2018[31] | Musculoskeletal pain | 3 | 76 | Exergames (yoga, mobility, core training, strength training) | Core strengthening, coordination, stabilization exercise | P | PEDro |
| Dong 2019[32] | Chronic musculoskeletal pain | 16 | 680 | Control exercise without WBV (squatting exercises, strengthening, home based, flexibility, lumbar stability training) | Whole body exercises (squatting exercises, strengthening, home based, flexibility, lumbar stability training) | P | PEDro |
| Hall 2008[33] | Musculoskeletal disorders | 5 | 717 | Aquatic exercise | Land exercise, wait list control, no treatment | P | SIGN |
| Hall 2009[34] | Chronic musculoskeletal pain conditions (OA hip, knee, lumbar spine and ankle), RA | 7 | 321 | Tai chi exercises | Control group | P, PF | PEDro |
| Lafrance 2021[35] | Upper or lower extremity musculoskeletal disorders | 19 | 1244 | Motor control exercises | Strengthening exercise | P, PF | Cochrane Risk of Bias tool |
| Miller 2021[36] | Chronic musculoskeletal conditions | 79 | 4719 | Exercise training combination | Placebo, usual care, true control | P | Cochrane Risk of Bias tool |
| Ouellet 2021[37] | Musculoskeletal disorders (neck pain, low back pain, knee OA) | 16 | 1719 | Region specific exercise (strengthening, stretching, proprioceptive, motor control exercises) | General exercise (yoga, walking, cycling, tai chi, Pilates, Qigong) | P, PF | Cochrane Risk of Bias tool |
| Polaski 2019[38] | Chronic pain conditions (OA, low back pain, neck pain, RA, PFPS) | 75 | NR | Exercise (aquatic, aerobic, strengthening) | No treatment | P | Cochrane Risk of Bias tool |
| Schafer 2018[39] | Knee OA, hip OA or both | 7 | 742 | Electronically-delivered exercise | Exercise, no treatment, waitlist control | P, PF | Cochrane Risk of Bias tool |
| Sieczkowska 2020[40] | OA, RA, fibromyalgia | 29 | 1899 | Resistance training | Exercise (aerobic, flexibility, walking), control, WBV | PF, MH | Cochrane Risk of Bias tool |
| Waller 2014[41] | Knee and hip OA | 11 | 1092 | Aquatic exercise | Usual care or control | P, PF | PEDro |
| Yan 2013[42] | Hip and Knee OA | 7 | 348 | Tai Chi | Waitlist control, education | P, PF | Jadad |
| Batterham 2011[43] | Hip and knee OA | 10 | 556 | Land based exercises | Water based exercises | PF | PEDro |
| Kong 2016[44] | Chronic musculoskeletal pain (OA, low back pain, fibromyalgia) | 18 | 1260 | Tai Chi | No treatment, physical therapy | P | PEDro |
| Denham-Jones 2021 (a)[45] | Musculoskeletal conditions | 7 | 397 | Pilates | No treatment | P, PF | Cochrane Risk of Bias tool |
| Zhang 2019 (a)[46] | OA | 32 | 3228 | Walking, strengthening exercise, Yoga, aquatic exercise | Exercise, education, usual care | P | Cochrane Risk of Bias tool |
| Hurley 2018[47] | Hip & knee OA | 21 | 2372 | Strength, resistance Training, Tai Chi, Yoga, water based exercise | Normal care, education, attention control | P, PF, MH | Cochrane Risk of Bias tool |
| Lauche 2019[48] | Lower extremity OA | 5 | 640 | Yoga | Aerobics, strengthening exercise, education control, usual care, TENS, ultrasound, meditation control | P, PF, MH | Cochrane Risk of Bias tool |
| Zampogna 2020[49] | Hip and knee OA | 19 | 1504 | Exercises (Tai chi, aquatic) | no intervention, medication, education | P, PF, MH | Cochrane Risk of Bias tool |
| Regnaux 2015[50] | Hip or knee OA | 6 | 656 | High-intensity exercise | Low-intensity exercise | P, PF, AE | Cochrane Risk of Bias tool |
| Siddall 2021[51] | Chronic musculoskeletal pain | 5 | 460 | Exercise (aquatic, strength training, multimodal exercise) | Pain neuroscience education and exercise | P, PF | PEDro |
| Denham‐Jones 2021 (b)[52] | Chronic musculoskeletal pain | 11 | 2221 | Yoga | Waitlist control, education, usual care | P, PF | Cochrane Risk of Bias tool |
| Smith 2017[53] | Chronic musculoskeletal pain (shoulder pain, ankle pain, plantar fascitis, low back pain, achilles pain | 9 | 447 | Painful exercises (eccentric resistance training) | Pain free exercises (resistance exercises) | P | Cochrane Risk of Bias tool |
| Franco 2021[54] | Fibromyalgia, chronic whiplash-associated disorders (CWAD) and chronic idiopathic neck pain (CINP) | 24 | 3562 | Aerobic exercise, Land-based aerobic, Muscle strengthening, aerobic exercise, motor control, Mind-body therapies | Muscle strengthening, Pool-based aerobic exercise, Muscle stretching, aerobic exercise, strengthening, combined exercise | P | Cochrane Risk of Bias tool |
| O'Connor 2015[55] | Fibromyalgia, chronic hip, lower back or knee pain, OA | 17 | 2384 | Walking | Control (stabilization exercise, education) | P, PF | United States Preventative Services Task Force (USPSTF) |
| Fransen 2002[56] | Hip and knee OA | 14 | 1633 | Unilateral quadriceps muscle strengthening and aerobic exercise, muscle strengthening, and balance coordination in addition to lower limb muscle strengthening | Education, no intervention, waitlist control, ROM exercises | P, PF | Instrument to Measure the Likelihood of Bias |
| Bartels 2016[57] | Knee and hip OA | 13 | 1190 | Aerobic exercise | Control | P, PF, AE | Cochrane Risk of Bias tool |
| Goh 2019[58] | Knee and hip OA | 103 | 9134 | Muscle strengthening, aerobic, or flexibility/neuro-motor skills training | Usual care or exercise | P, PF | Cochrane Risk of Bias tool |
| Verhagen 2013[59] | Non-specific neck, shoulder pain and rotator cuff tendinitis | 29 | 6580 | Proprioceptive neuromuscular facilitation, Feldenkrais therapy or Mensendieck training | No treatment, exercises, massage, manual therapy | P, PF | Cochrane Risk of Bias tool |
| Wang 2018[60] | Knee OA and rheumatoid arthritis | 13 | 1557 | Yoga | Exercise, waitlist, no treatment, education, physiotherapy | P, PF | Jadad |
| Escalante 2010[61] | Lower limb OA | 33 | 2679 | Strength training, tai chi, aerobic exercise | Education, no treatment | P | Delphi list |
| Fernandopulle 2017[62] | Hip and knee OA | 14 | 3273 | Walking intervention, recreational activity, and conditioning exercise protocol | Control (no intervention, education) | P, PF | Cochrane Back and Neck Review Criteria |
| Ferreira 2006[63] | Cervical, thoracic, low back, or pelvic pain | 12 | NR | Specific stabilisation exercise | Education, physiotherapy, swimming, walking | P, PF | PEDro |
| Skelly 2020[64] | Chronic low back pain, chronic neck pain, OA (knee, hip, hand), fibromyalgia | 233 | NR | Exercise, mind body therapies (Yoga, tai chi) | Usual Care, Waitlist, No treatment, attention control, or sham | P, PF, MH | Cochrane Risk of Bias tool |
| Weng 2023[65] | Knee or hip OA | 106 | 9497 | Mixed exercise, mind body exercise, strength, flexibility, aerobic, neuromotor exercises | Usual care, no treatment | P, PF | Cochrane Risk of Bias tool |
| Wen 2022[66] | Chronic Pain | 17 | 1481 | Mind-body exercises | Education, physical therapy, waitlist, exercise, quadriceps strengthening exercise | P, PF, MH | Cochrane Risk of Bias tool |
| Amiri 2022[67] | Musculoskeletal pain | 19 | 1396 | Exercise training | Exercise, waitlist, passive modalities, education, manual therapy, cognitive behavioural therapy | MH | Cochrane Risk of Bias tool |
| Thompson 2023[68] | Musculoskeletal pain (knee OA, low back pain, ankle sprains, frozen shoulder, neck pain, and wrist, hand and finger injuries) | 11 | 845 | App-based strengthening, balance and stretching exercises | Exercise, education without app | P, PF, AR | Cochrane Risk of Bias tool |
| Thorlund 2022[69] | Knee and hip OA | 13 | 1398 | Exercise therapy | Placebo control | P | Cochrane Risk of Bias tool |
| French 2022[70] | Knee and hip OA | 60 | 6508 | Exercise therapy | Exercise therapy and adjuvant electrophysical agents | P, PF, AE | Cochrane Risk of Bias tool |
| Duan 2022[71] | Knee and hip OA | 19 | 1592 | Aqautic exercises | Unsupervised home exercises or usual care | P, PF | Cochrane Risk of Bias tool |
| Song 2022[72] | OA | 12 | 1609 | Aquatic exercises | No treatment, aquatic and stretching exercises | P, PF, MH | Cochrane Risk of Bias tool |
| Ortega-Castillo 2022[73] | Upper limb tendinopathy | 4 | 970 | Progressive exercise training (e.g. isometric, concentric, eccentric, plyometric, other type of exercise) | Home exercise, resistance exercise and supervised exercise | P, PF | PEDro |
| Cuenca-Martinez 2022[74] | LBP, subacromial pain syndrome, fibromyalgia | 13 | NR | High-intensity interval training | Minimal intervention, no intervention, and usual care with or without placebo interventions | P, PF, MH | Cochrane Risk of Bias tool |
| Silva 2022[75] | Musculoskeletal Conditions (LBP, PFPS) | 9 | 503 | Hip Strengthening, lumbar stabilization and knee strengthening exercises | Placebo, waiting list, no treatment or other active interventions | P, PF | PEDro |
| Runge 2022[76] | Knee or Hip OA | 17 | 1394 | Aerobic, strengthening, stretching and stabilizing exercises | Manual therapy with exercises therapy | P, PF | Cochrane Risk of Bias tool |
| Elbow pain |  |  |  |  |  |  |  |
| Karanasios 2021[77] | Lateral elbow tendinopathy | 21 | 2123 | Eccentric exercise | Concentric exercise with or without physiotherapy or home exercise program | P, PF | Cochrane Risk of Bias tool |
| Yoon 2021[78] | Lateral elbow tendinopathy | 6 | 429 | Eccentric exercise | Resitance training (concentric or isotonic exercise) and physiotherapy | P, PF | Cochrane Risk of Bias tool |
| Wood 2022[79] | Lateral epicondylitis | 23 | 1363 | Exercise | Electrotherapy, placebo | P | Cochrane Risk of Bias tool |
| Hand pain |  |  |  |  |  |  |  |
| Magni 2017[80] | Hand OA | 5 | 350 | Resistance training | Usual care, sham, no treatment | P, PF | Cochrane Risk of Bias tool |
| Osteras 2017[81] | Hand OA | 5 | 534 | Stretching and strengthening exercise | Exercise, no exercise | P, PF | Cochrane Risk of Bias tool |
| Bertozzi 2015[82] | Carpometacarpal OA | 13 | 1145 | Therapeutic exercises (resistance exercises) | Control | P, PF | PEDro |
| Hip pain |  |  |  |  |  |  |  |
| Fransen 2010[83] | Hip OA | 5 | 267 | Land based exercises Combination (strengthening, ROM, Tai chi) | Control treatment | P, PF | Jadad |
| Fransen 2014[84] | Hip OA | 10 | 521 | Land based exercises | Waitlist control group | P, PF, AR | Cochrane Risk of Bias tool |
| Hansen 2020[85] | Hip OA | 3 | 189 | Supervised resistance training | Exercise and education | P, PF | Cochrane Risk of Bias tool |
| Moseng 2017[86] | Hip OA | 12 | 1202 | Land-based supervised exercise (strengthening, flexibility, cardiorespiratory exercises) | No treatment | P, PF | Cochrane Risk of Bias tool |
| Sampath 2015[87] | Hip OA | 7 | 886 | Strengthening exercise and flexibility exercise | Manual therapy and strengthening exercise | P, PF | Cochrane Risk of Bias tool |
| Hernandez-Molina 2008[88] | Hip OA | 9 | 1234 | Aquatic, land based, Tai chi | Control | P | Rochon scale |
| Beumer 2016[89] | Hip OA | 19 | NR | Water based and land based (hip stretches, strengthening exercises and balance tasks) | Manual therapy, no treatment, exercise therapy, and minimal control | P | PEDro |
| Knee pain |  |  |  |  |  |  |  |
| Corbett 2013[90] | Knee OA | 114 | 13700 | Muscle strengthening exercises | Placebo, no exercise, physical therapy | P | CRD Checklist |
| Tanaka 2014[91] | Knee OA | 17 | 1816 | Land-based therapeutic exercise (strengthening, aerobic) | No intervention or psychoeducational intervention | P | PEDro |
| Lauche 2013[92] | Knee OA | 5 | 252 | Tai chi | Waitlist control | P, PF, MH | Cochrane Back and Neck Review Criteria |
| Raj 2018[93] | Knee OA | 9 | 826 | Isokinetic exercise | Education, no treatment, exercise, conservative treatment modalities | P | PEDro |
| Nascimento 2018[94] | Patellofemoral pain | 14 | 673 | Hip and knee strengthening | Placebo, no treatment, education | P, PF | PEDro |
| Tanaka 2013 (a)[95] | Knee OA | 33 | 3192 | Aerobic, functional strengthening, and flexibility exercises, Baduanjin, Tai Chi | No intervention and psychoeducational intervention | P | PEDro |
| Wang 2012 (a)[96] | Knee OA | 84 | 4297 | Strengthening, Tai chi, aerobic, proprioception exercise | Sham, usual care, no active treatment | P, PF, AR | Cochrane Risk of Bias tool |
| Clijsen 2014[97] | Patellofemoral Pain Syndrome | 15 | 748 | Strength training | No exercise or exercise | P, PF | PEDro |
| Li 2021[98] | Knee OA | 9 | 369 | Resistance training | Blood-flow restriction training | P | Cochrane Risk of Bias tool |
| Imoto 2019[99] | Knee OA | 55 | 4368 | Land-based exercises | No exercise and waitlist control | P | Cochrane Risk of Bias tool |
| Jeong 2019[100] | Knee OA | 6 | 558 | Proprioceptive training | Strength training, physical therapy, | P, PF | PEDro |
| Li 2016[101] | Knee OA | 17 | 1705 | Resistance training | Non-intervention or psycho-educational intervention | P, PF | Cochrane Risk of Bias tool |
| Grantham 2021[102] | Knee OA | 4 | 199 | Resistance training | Blood flow restriction training | P, PF | PEDro |
| Coudeyre 2016[103] | Knee OA | 9 | 696 | Isokinetic muscle strengthening exercises | Isokinetic muscle strengthening and ultrasound, "control group", educational program | P, PF | CheckList to Evaluate A Report (CLEAR) scale |
| Hu 2021[104] | Knee OA | 16 | 986 | Tai Chi exercises | Education class | P, PF, MH | Cochrane Risk of Bias tool |
| Jansen 2011[105] | Knee OA | 12 | 1262 | Strength training | Placebo/ nothing | P, PF | Evidence Based Richtlijn Ontwikkeling (EBRO) |
| Smith 2012[106] | Knee OA | 7 | 560 | Proprioceptive exercise regime | Non-proprioceptive exercise programme or non-treatment control | P, PF, AR | PEDro |
| Tanaka 2013 (b)[107] | Knee OA | 8 | 466 | Strengthening or aerobic exercise | Non-intervention or psycho-educational intervention | P | PEDro |
| Dong 2018[108] | Knee OA | 8 | 579 | Aquatic exercise | Land based exercise | P, PF, MH | Cochrane Risk of Bias tool |
| Anwer 2016[109] | Knee OA | 16 | 4270 | Home exercise program | Inpatient/outpatient physical therapy, and no intervention | P, PF | Cochrane Risk of Bias tool |
| Cuyul-Vásquez 2020[110] | Knee OA, patellofemoral pain, anterior knee pain | 5 | 340 | Resistance exercises (strengthening, stretching, cycling) | Blood flow restriction plus resistance training | P, PF | Cochrane Risk of Bias tool |
| Fransen 2015[111] | Knee OA | 54 | 5362 | Land-based non-perioperative therapeutic exercise regimens (aerobic, strengthening, ROM, resistance, balance, motor control, Tai chi) | Usual care, exercise, physiotherapy | P, PF, AR | Cochrane Risk of Bias tool |
| Manojlovic 2021[112] | Patellofemoral pain | 13 | 1199 | Strength training | Strengthening exercises or no treatment control | P, PF | PEDro |
| Lu 2015[113] | Knee OA | 6 | 398 | Aquatic exercise | Land-based exercise, non-exercise | P, PF | Cochrane Risk of Bias tool |
| Luan 2021[114] | Knee OA | 8 | 724 | Cycling | Tai chi, Baduanjin, land and water treadmill, no exercise | P, PF | PEDro |
| Bartholdy 2017[115] | Knee OA | 45 | 4699 | Strengthening exercises, Baduanjin exercise, ROM exercise, isometric exercise, isokinetic exercise | Other exercise types | P, PF | Cochrane Risk of Bias tool |
| Rogan 2019[116] | Patellofemoral pain | 7 | 604 | Strength training | No hip strengthening or knee muscle strengthening | P, PF | Cochrane Risk of Bias tool |
| Heijden 2015[117] | Patellofemoral pain syndrome | 19 | 1690 | Exercise therapy (different variables/modes of training) | Control, unimodal and multimodal conservative interventions | P, PF, AE | Cochrane Risk of Bias tool |
| Newberry 2017[118] | Knee OA | 15 | NR | Aerobic, strengthening, Tai chi, yoga, general exercise, agility exercises | Exercise, education | P, PF | Cochrane Risk of Bias tool |
| Li 2015[119] | Knee OA | 5 | 168 | Squat exercise and home based exercise | WBV plus exercise | P, PF | Cochrane Risk of Bias tool |
| Roddy 2005[120] | Knee OA | 13 | 2304 | Strengthening, ROM, walking, isometric, aerobic exercise | Education, resistance training, physical therapy | P, PF | Jadad |
| Winters 2020[121] | Patellofemoral pain | 16 | 1472 | Resistance training | Education, orthotics | P, PF | Cochrane Risk of Bias tool |
| Hislop 2020[122] | Knee OA | 8 | 341 | Hip and quadriceps exercises (resistance, stair climbing) | Quadriceps exercises only (resistance training) | P, PF | PEDro |
| Devos-Comby 2006[123] | Knee OA | 16 | 2154 | Walking, aerobic, resistance, balance and coordination exercises | Self-management education | P, PF, MH | NR |
| Li 2020[124] | Knee OA | 14 | 815 | Tai Chi and Baduanjin exercises | Attention control, physical therapy | P, PF | Cochrane Risk of Bias tool |
| Zeng 2020[125] | Knee OA | 7 | 424 | Baduanjin exercises | Waitlist control, education | P, PF | Cochrane Risk of Bias tool |
| Anwer 2018[126] | Knee OA | 11 | 494 | Exercise therapy | Manual therapy | P, PF | PEDro |
| Cardoso 2017[127] | Patellofemoral pain syndrome | 5 | 377 | Resistance and stretching exercises | No treatment | P | NR |
| Chen 2021[128] | Knee OA or chronic knee pain | 12 | NR | Technology-supported exercise programs | Conventional care, such as face-to-face physical therapy, or education, minimal or no care | P, PF | Cochrane Risk of Bias tool |
| Wang 2021 (a)[129] | Knee OA | 5 | 182 | Resistance therapy alone | Blood flow restriction therapy plus low-load resistance therapy | P, PF, AE | Cochrane Risk of Bias tool |
| Juhl 2014[130] | Knee OA | 48 | 2732 | Aerobic exercise, resistance exercises | Education | P, PF | Cochrane Risk of Bias tool |
| Na 2021[131] | Patellofemoral pain syndrome | 5 | 364 | Isolated hip strengthening exercises | Traditional knee-based strengthening | P, PF | PEDro |
| Scali 2018[132] | Patellofemoral pain syndrome | 5 | 242 | Multi-Joint strengthening exercises | Single Joint strengthening exercises | P, PF | PEDro |
| Silva 2020[133] | Patellofemoral pain syndrome | 8 | 731 | Standardized home exercise program and personalized program | Education | P, PF | Cochrane Risk of Bias tool |
| Goff 2021[134] | Knee OA | 29 | 4107 | Exercise therapy | Education | P, PF | Cochrane Risk of Bias tool |
| Lack 2015[135] | Patellofemoral pain syndrome | 12 | NR | Proximal rehabilitation | Quadriceps rehabilitation | P, PF | PEDro |
| Rocha 2020[136] | Knee OA | 5 | 934 | Quadriceps strengthening exercises | No intervention, knee exercises, TENS | P | PEDro |
| Zhang 2017[137] | Knee OA | 8 | 375 | Traditional Chinese Exercise (Tai Chi) | No treatment, waitlist control, attention control (education) | P, PF, MH | Cochrane Risk of Bias tool |
| Chen 2019[138] | Knee OA | 6 | 432 | Aquatic exercises | Aquatic exercises, normal physical activity, education | P, PF | Cochrane Risk of Bias tool |
| Alammari 2023[139] | Patellofemoral pain | 9 | 383 | Hip and quadriceps strengthening exercises | Quadriceps strengthening exercises | P, PF, AE | Cochrane Risk of Bias tool |
| Guo 2022 (a)[140] | Knee OA | 15 | 1436 | Resistance, aerobic, stretching exercises | No exercise, exercise, sham, electrotherapy modality | P, PF | Cochrane Risk of Bias tool |
| Guo 2022 (b)[141] | Knee OA | 7 | 668 | Wu Qin Xi exercises | Isokinetic training, no exercise, standing exercise, physical therapy | P, PF | Cochrane Risk of Bias tool |
| Wu 2022 (b)[142] | Knee OA | 16 | 642 | Exercise therapy | Kinesiotaping plus exercise therapy | P, PF | Cochrane Risk of Bias tool |
| Wang 2021 (b)[143] | Knee OA | 24 | 1275 | Proprioceptive Training, resistance training | No intervention, resistance exercise, conventional physiotherapy | P, PF | PEDro |
| AL-Mhanna 2022[144] | Knee OA | 7 | 346 | Circuit training | Usual care | P, PF | Cochrane Risk of Bias tool |
| Hua 2022[145] | Knee OA | 10 | 892 | High-intensity strength training | Low-intensity strength training, education and no treatment | P, PF, AE | Cochrane Risk of Bias tool |
| Challoumas 2021[146] | Patellar tendinopathy | 37 | 1332 | Eccentric and isometric exercises and placebo | Shockwave therapy | P, PF | Cochrane Risk of Bias tool |
| Pedersen 2022[147] | Knee OA | 35 | 2412 | Exercise therapy | Passive modalities, no intervention, education, exercise therapy | P, PF | Cochrane Risk of Bias tool |
| Jurado-Castro 2022[148] | Knee OA | 6 | 410 | Exercise therapy | No intervention, usual care | P, PF | Cochrane Risk of Bias tool |
| Neal 2022[149] | Patellofemoral pain | 65 | 3796 | Knee targeted exercise therapy | Hip and knee targeted exercise, Wait and see control | P, PF | PEDro |
| Xu 2023[150] | Knee OA | 22 | 1394 | Aquatic exercises | Walking, resistance exercise, land based, no exercise, usual care | P, PF | PEDro |
| Thomas 2022[151] | Knee OA | 3 | 388 | Hip abductor strengthening exercises | Strengthening exercises, no intervention, electrotherapy | P, PF | PEDro |
| Qiu 2022[152] | Knee OA | 10 | 559 | Squat training and strengthening exercises | WBV plus exercises | P, PF | Cochrane Risk of Bias tool |
| Yang 2022 (a)[153] | Patellofemoral pain | 5 | 174 | Home exercise and conventional exercises | WBV plus same exercise program | P, PF, MH | Cochrane Risk of Bias tool |
| Luan 2022[154] | Knee OA | 18 | 1250 | Stretching, strengthening, balance, coordination, flexibility | Physiotherapy modalities, no exercise, other exercises | P | Cochrane Risk of Bias tool |
| Low back pain |  |  |  |  |  |  |  |
| Amaral 2020[155] | Non-specific low back pain | 11 | 758 | Mixed exercise (aerobic, flexibility, strengthening, yoga) | Biofeedback exercise, Qigong, physical exercise and cognitive behaviour therapy | P, PF | PEDro |
| Hayden 2020[156] | Persistent Low back pain | 27 | 3514 | Exercise | Education, manual therapy, usual care | P, PF | Cochrane Back and Neck Review Criteria |
| Hayden 2021[157] | Chronic low back pain | 249 | 24486 | Core strengthening, Pilates, general strengthening exercises, stretching, and aerobic exercise | No treatment, land other conservative treatments, education, physical therapy (multi-modal non-exercise), manual therapy, electrotherapy, or psychological therapy | P, PF | Cochrane Risk of Bias tool |
| Holtzman 2013[158] | Chronic low back pain | 8 | 743 | Yoga | No treatment, usual care, waitlist, exercise, education | P, PF | CheckList to Evaluate A Report (CLEAR) scale |
| Jacobi 2021[159] | Degenerative lumbar spinal stenosis | 5 | 1432 | Walking, trunk muscle exercise, strengthening, aquatic exercise (Self-directed or group exercises) | Exercise and manual therapy | P, PF | Cochrane Risk of Bias tool |
| Keller 2007[160] | Non-specific low back pain | 4 | 763 | Supervised exercise | No treatment, sham treatment | P, PF | Cochrane Risk of Bias tool |
| Lim 2011[161] | Persistent non-specific low back pain | 7 | 194 | Pilates | Passive interventions, exercise, usual care, physical modalities | P, PF | Delphi list |
| Luomajoki 2018[162] | Non-specific low back pain | 11 | 781 | Motor control exercise | Education, no treatment, stabilization exercise | P, PF | PEDro |
| Macedo 2016[163] | Acute and subacute non-specific low back pain | 3 | 197 | Motor control exercise | Specific exercises and spinal manipulative therapy | P, PF | Cochrane Risk of Bias tool |
| Maciel 2018[164] | Low back pain | 5 | 483 | Yoga, stretching, strengthening, stabilization, aerobic exercises | Segmental stabilization exercise, usual care | P | PEDro |
| Meng 2015[165] | Chronic low back pain | 8 | 310 | Aerobic Exercise | Physiotherapy, exercise | P, PF | Newcastle-Ottawa scale criteria |
| Miyamoto 2013[166] | Chronic non-specific low back pain | 7 | 363 | Pilates | No treatment, minimal intervention, Pilates | P, PF | PEDro |
| Mueller 2020[167] | Chronic non-specific low back pain | 50 | 2786 | Motor control exercises, Stabilization exercise | Exercise, physiotherapy, manual therapy, no treatment | P, PF | PEDro |
| Nduwimana 2020[168] | Chronic low back pain | 31 | 3193 | Walking | Mind-body therapies (yoga, Tai chi, qigong, mindfullness meditation) | P, PF | PEDro |
| Owen 2020[169] | Chronic non-specific low back pain | 82 | 5578 | Exercise | No control, hands-on and hands-off control | P, PF, MH | Cochrane Risk of Bias tool |
| Parreira 2017[170] | Chronic non-specific low back pain | 22 | 4105 | Exercise | Back school | P, PF | Cochrane Risk of Bias tool |
| Pereira 2012[171] | Chronic low back pain | 5 | 139 | Pilates | No exercise or lumbar stabilization exercise | P, PF | Cochrane Risk of Bias tool |
| Pourahmadi 2020[172] | Low back pain | 18 | 965 | Proprioceptive neuromuscular facilitation training | Physical therapy | P, PF | PEDro |
| Quentin 2021[173] | Non-specific low back pain | 33 | 9588 | Strengthening exercises | Exercise, Manual therapy, massage, cognitive behaviour therapy | P, PF | SIGN |
| Searle 2015[174] | Non-specific chronic low back pain | 39 | 4462 | Strengthening, cardiorespiratory, coordination exercise | Wait list or usual activities, general practitioner care, electrotherapies (ultrasound, laser) and manipulative therapies (physiotherapy, massage, osteopathy) | P | Downs and Black tool |
| Shi 2018[175] | Low back pain | 8 | 331 | Aquatic Exercises | Land-based therapy, standard general practice, or no exercise | P, PF, MH | Cochrane Risk of Bias tool |
| Sitthipornvorakul 2018[176] | Chronic low back pain | 9 | 863 | Walking | Exercise, education | P, PF | Cochrane Risk of Bias tool |
| Slade 2007[177] | Non-specific chronic low back pain | 6 | 830 | Yoga | Aerobic and trunk strengthening, no exercise | P, PF | PEDro |
| Smith 2014[178] | Non-specific low back pain | 29 | 1559 | Stabilization exercise | Physiotherapy, placebo, no treatment, control | P, PF | PEDro |
| Sun 2021[179] | Non-specific chronic low back pain | 31 | 7116 | Yoga, physical exercise, low back exercise | Exercise, education, multidisciplinary intervention | P | Cochrane Risk of Bias tool |
| Tataryn 2021[180] | Chronic low back pain | 8 | 408 | Posterior chain resistance training | General exercise | P, PF, AE | Downs and Black tool |
| Thornton 2021[181] | Low back pain | 4 | 541 | Resistance training and core stability exercises | Exercise, massage | P, PF | Cochrane Risk of Bias tool |
| van Middelkoop 2011[182] | Chronic non-specific low back pain | 83 | 8816 | Motor control exercises, yoga, aerobic exercise | Manipulation, exercises, usual care, psychotherapy, electrotherapy | P, PF | Cochrane Back and Neck Review Criteria |
| Wood 2021[183] | Persistent non-specific low back pain | 27 | 5870 | Strengthening exercise, Tai chi, yoga, aerobic, motor control exercises | No treatment, usual care, physiotherapy | P, PF | Cochrane Risk of Bias tool |
| Yamato 2015[184] | Low back pain | 9 | 510 | Pilates | General exercise, cycling, usual care | P, PF | Cochrane Risk of Bias tool |
| Yue 2014[185] | Chronic low back pain | 9 | 706 | Sling exercise | Massage, exercise | P, PF | Cochrane Risk of Bias tool |
| Zhu 2020[186] | Chronic low back pain | 18 | 1814 | Yoga | Usual care, no treatment | P, PF | Cochrane Risk of Bias tool |
| Zou 2019[187] | Chronic low back pain | 17 | 2022 | Mindfullness therapis (Tai chi, yoga, Qigong) | Education, waitlist, exercise | P, PF | PEDro |
| Li 2019 (a)[188] | Low back pain | 9 | 519 | Baduanjin exercises | General exercises | P, PF | Cochrane Risk of Bias tool |
| Tomazoni 2020[189] | Low back pain | 2 | NR | Strengthening, stretching, mobilising, co-ordination and stabilization exercises | Photo-biomodulation therapy and same exercises as intervention group | P, PF | PEDro |
| Zhang 2019 (b)[190] | Low back pain | 11 | 886 | Traditional Chinese exercises | Exercise, physiotherapy, no treatment, waitlist control | P, PF | PEDro |
| Saragiotto 2016[191] | Non-specific low back pain | 32 | 2628 | Motor control exercise | Other exercises, minimal intervention | P, PF | Cochrane Back and Neck Review Criteria |
| Hayden 2005[192] | Low back pain | 61 | 6390 | Home exercise, endurance, exercise therapy, strengthening, trunk stabilizing exercises | No treatment, usual care, conservative interventions | P, PF | Cochrane Risk of Bias tool |
| Gianola 2021[193] | Mechanical non-specific low back pain | 35 | 8765 | Exercises | Conservative interventions (Education, heat wrap, acupuncture, manual therapy), inert treatment | P, PF | Cochrane Risk of Bias tool |
| Wewege 2018[194] | Chronic non-specific low back pain | 6 | 333 | Land-based PAT, PRT or combined PRT and PAT | Usual care, advice, waitlist group | P, PF, MH | PEDro |
| Freitas 2020[195] | Chronic non-specific low back pain | 5 | 507 | Mat or equipment Pilates | Education, Pilates, advice, no treatment | MH | Cochrane Risk of Bias tool |
| Niederer 2020[196] | Chronic non-specific low back pain | 10 | 1081 | Motor control and stabilization exercises | Active, passive approaches or exercises | P, PF | Cochrane Risk of Bias tool |
| Vanti 2019[197] | Chronic low back pain | 5 | 329 | Walking plus exercise | Exercise | P, PF | PEDro |
| Jesus 2020[198] | Non-specific low back pain | 5 | 309 | Hip strengthening exercise, physiotherapy exercises, lumbopelvic stabilization exercises | Lumbopelvic stabilization exercises, physiotherapy exercises, hip stretching | P, PF | PEDro |
| Fernandez 2015[199] | Sciatica | 5 | 604 | Exercise | Advice | P, PF | PEDro |
| Wang 2012 (b)[200] | Chronic low back pain | 5 | 414 | Core stability exercise | General exercise | P, PF | Cochrane Risk of Bias tool |
| Aladro-Gonzalvo 2013[201] | Persistent non-specific low back pain | 9 | 223 | Pilates | Placebo treatment, minimal intervention or another physiotherapeutic treatment | P, PF | PEDro |
| Anheyer 2021[202] | Low back pain | 30 | 2702 | Yoga | Passive control intervention (written advice, usual care, waitlist control), active control intervention (stabilization, strengthening, aerobic, physical therapy, back school) | P, PF, AE | Cochrane Risk of Bias tool |
| Byström 2013[203] | Chronic and recurrent low back pain | 16 | 1768 | Motor control exercise | General exercise, spinal manual therapy, minimal intervention, multimodal physical therapy | P, PF | PEDro |
| Drummond 2021[204] | Chronic low back pain | 12 | 631 | Sling exercise therapy | General exercise, motor control training/lumbar stabilization, modalities, and no treatment | P, PF | Cochrane Risk of Bias tool |
| Ebadi 2020[205] | Chronic low back pain | 8 | 1025 | Exercise therapy | Ultrasound therapy | P, PF | Cochrane Back and Neck Review Criteria |
| Ford 2020[206] | Low back pain | 28 | 2323 | Specific muscle activation exercises | Manual therapy, strengthening, walking, stretching, physiotherapy, minimal intervention | P, PF | PEDro |
| Gomes-Neto 2017[207] | Low back pain | 11 | 1014 | Stabilization exercises | General exercise and manual therapy | P, PF | Cochrane Risk of Bias tool |
| Ram 2023[208] | Low back pain | 4 | 214 | High-intensity exercises (Ergometer exercises, lumbar extension machine, aerobic training) | Low-intensity exercises (Ergometer exercises, lumbar extension machine, aerobic training) | P, PF | Cochrane Risk of Bias tool |
| Ling-Xin 2022[209] | Low back pain | 4 | 184 | PNF training | Sham PNF, routine care, education | P, PF | PEDro |
| Gao 2022[210] | Low back pain | 12 | 410 | PNF training | General trunk exercise, ball exercises, education, physical therapy, no intervention | P, PF | Cochrane Risk of Bias tool |
| Shanbehzadeh 2022[211] | Low back pain | 13 | NR | Motor control training | Mckenzie, no treatment, general exercises | P, PF | Modified Downs and Black score |
| Zhang 2022 (b)[212] | Lumbar spondylosis and low back pain | 7 | 296 | Tai Chi | No treatment, education, massage, physiotherapy | P, PF | PEDro |
| Temporiti 2022[213] | Lumbar spinal stenosis | 2 | NR | Exercises (aerobic, stretching, strengthening) | Physical therapy, placebo, ultrasound therapy | P, PF | PEDro |
| Arcanjo 2022[214] | Non-specific low back pain | 11 | 677 | PNF exercises | Core stabilization exercises, TENS, physiotherapy, Swiss ball training | P, PF | PEDro |
| Pocovi 2022[215] | Non-specific low back pain | 19 | 2362 | Walking, Cycling, and Swimming | Stabilization exercise, physical exercise, advice, usual care, education | P, PF | Cochrane Risk of Bias tool |
| Prat-Luri 2023[216] | Low back pain | 40 | NR | Trunk-focused targeted exercises | No intervention, minimal intervention, or hands-on/off treatment, exercises | P, PF | Cochrane Risk of Bias tool |
| Pourahmadi 2022[217] | Lumbar disc herniation | 16 | 861 | Motor control training (MCT) | Exercise interventions, placebo, sham, minimal/no intervention | P, PF | Cochrane Risk of Bias tool |
| Ranjan 2023[218] | Low back pain | 11 | NR | PNF exercises | General exercise, conventional physical therapy, general stretching or ball exercise | P, PF | Cochrane Risk of Bias tool |
| Sutanto 2022[219] | Chronic low back pain | 47 | 2299 | Isometric trunk muscle training, isotonic training, motor control training | Advice, education, placebo, waitlist treatment, different trunk training, passive intervention | P, PF | Cochrane Risk of Bias tool |
| Wieland 2022[220] | Non-specific low back pain | 21 | 2223 | Yoga | Education, exercise, waitlist control, usual care | P, PF, MH, AE | Cochrane Risk of Bias tool |
| Shi 2022[221] | Non-specific low back pain | 36 | 3050 | Yoga, qigong, Tai chi, Pilates | Conventional therapeutic exercises, usual care, no treatment | P, PF | Cochrane Risk of Bias tool |
| Zhang 2021[222] | Low back pain | 18 | 1333 | Motor control exercises | Placebo, manual therapy, exercises | P, PF | Cochrane Risk of Bias tool |
| Fernández-Rodríguez 2022[223] | Low back pain | 118 | 9710 | Exercises (Pilates, mind-body exercises, core strengthening, combined, aerobic) | No exercise, usual practice, physical exercise | P, PF | Cochrane Risk of Bias tool |
| Gilliam 2022[224] | Low back pain | 8 | 558 | Mind-body exercises (yoga and Pilates) | Stabilization exercises, control treatment, physiotherapy, education | P, PF | Cochrane Risk of Bias tool |
| Fleckenstein 2022[225] | Low back pain | 53 | 10084 | Exercise therapy | Sensorimotor training, aerobic exercises, psychological therapy | P, PF | Cochrane Risk of Bias tool |
| Zhang 2022 (c) [226] | Low back pain | 18 | 910 | Swiss ball exercises, muscle training, resistance training, yoga, Tai chi, core stabilization, Qigong, stretching, aquatic exercises | Passive treatment, trunk stabilization, no intervention | P, PF | Cochrane Risk of Bias tool |
| Neck pain |  |  |  |  |  |  |  |
| de Zoete 2020[227] | Chronic non-specific neck pain | 40 | 3503 | Combination of exercises (proprioceptive, strengthening, stretching, motor control, yoga, Pilates, Tai chi, Qigong | No treatment, exercise combination | P, PF | PEDro |
| Cox 2019[228] | Non specific neck pain | 5 | 713 | Neck-specific strengthening exercise | Neck-specific strengthening exercise, cognitive behavioural therapy or combination of both | P, PF | Cochrane Risk of Bias tool |
| Gross 2015[229] | Mechanical neck disorders | 2 | 5490 | Cervical strengthening, stabilization, cervical stretching, ROM exercise | No intervention or waitlist | P, PF | Cochrane Risk of Bias tool |
| Leaver 2010[230] | Non-specific neck pain | 33 | 650 | Proprioceptive exercise, neck stabilization, gymnastics, muscle strengthening, general strength and conditioning exercises | Minimal intervention | P, PF | PEDro |
| Li 2019 (b)[231] | Chronic non-specific neck pain | 10 | 686 | Yoga | Pilates, Tai chi, isometric exercises | P, PF, MH | Cochrane Risk of Bias tool |
| Lin 2021[232] | Neck pain | 11 | 595 | Sling exercise (stabilization and muscle coordination) | Acupuncture, education, stretching exercises | P, PF | PEDro |
| Louw 2017[233] | Non-specific neck pain | 8 | 2075 | Strengthening, stretching and endurance exercises | Strengthening and endurance exercises | P | PEDro |
| Martin-Gomez 2019[234] | Non-specific chronic neck pain | 10 | 423 | Motor control exercises (Cranio-cervical flexion exercises) | Exercise, mobilization, proprioceptive exercise | P, PF | Cochrane Risk of Bias tool |
| Nunes 2015[235] | Chronic trapezius myalgia | 17 | 944 | Strength, coordination, endurance exercises | Stress management, exercise, health promotion (education) | P | Cochrane Risk of Bias tool |
| Salt 2011[236] | Cervical radiculopathy | 2 | 1036 | Strengthening and ROM exercises | Manual therapy and exercises | P | PEDro |
| Tsiringakis 2020[237] | Neck pain | 10 | 797 | Motor control training | Endurance strength training | P, PF | PEDro |
| Wu 2020[238] | Neck pain | 6 | 395 | Stabilization exercises | General exercise | P, PF, MH | Jadad |
| Chrcanovic 2021[239] | Whiplash associated neck pain | 5 | 2127 | Aerobic and resistance training | Education, physical therapy, no treatment | P, PF | Cochrane Risk of Bias tool |
| Wilhelm 2020[240] | Neck pain | 14 | 1708 | Exercise (strength, endurance, coordination, Pilates, stretching, proprioception, Qigong) | Neuro-education | P, PF | Cochrane Risk of Bias tool |
| Griffin 2017[241] | Whiplash-associated neck pain | 3 | 386 | Motor control, aerobic exercise, supervised and home exercise program | Advice, education | P, PF | PEDro |
| Fredin 2017[242] | Neck pain | 7 | 936 | Strengthening, ROM, stretching, warmup exercises, progressive resistive exercises, cervical stability training | Exercise plus manual therapy | P, PF, MH | PEDro |
| Bertozzi 2013[243] | Chronic nonspecific neck pain | 7 | 879 | Strengthening, stretching, endurance, resistance exercises | Exercise, no treatment, education | P, PF | PEDro |
| Garzonio 2022[244] | Non-specific neck pain | 9 | 717 | Craniocervical flexion training | Exercise, usual care, mobilization, waitlist control | P | Cochrane Risk of Bias tool |
| Villanueva-Ruiz 2022[245] | Neck pain | 12 | 468 | Specific tailored and non-tailored exercises | Non-specific tailored and non-tailored exercises | P, PF | Cochrane Risk of Bias tool |
| Castellini 2022[246] | Non-specific neck pain | 93 | 12496 | Mind body exercises and other exercises | Manual therapy, physical agents, exercises, kinesio taping, traction, usual care | P, PF | Cochrane Risk of Bias tool |
| Xie 2021[247] | Neck pain | 6 | 716 | Traditional Chinese mind and body exercises | Exercise, manual therapy and no treatment | P, PF, MH | PEDro |
| Kong 2022[248] | Neck Pain | NR | 1770 | Traditional Chinese exercises | Exercise, massage, Tuina massage, waitlist, traction, physical therapy, education | P, PF, MH | PEDro |
| Yang 2022 (b)[249] | Neck pain | 18 | 1891 | Isometric training | Traditional Chinese medicine, non-isometric exercise, no intervention | P, PF | Cochrane Risk of Bias tool |
| Rheumatoid arthritis | |  |  |  |  |  |  |
| Baillet 2010[250] | Rheumatoid Arthritis | 14 | 1040 | Cardiorespiratory aerobic conditioning | Usual care, education, ROM exercise, non-aerobic exercise | P, PF, AE, AR | Jadad |
| Baillet 2012[251] | Rheumatoid arthritis | 10 | 547 | Resistance exercises | Usual care, education, ROM exercise, non-aerobic exercise | P, PF, AE, AR | Jadad |
| Hurkmans 2009[252] | Rheumatoid arthritis | 8 | 575 | Dynamic exercise (Aerobic and muscle strength training) | ROM exercise, no exercise, education | P, PF | Cochrane Risk of Bias tool |
| Mudano 2019[253] | Rheumatoid arthritis | 7 | 345 | Tai chi | No exercise, alternative exercise therapy, education | P, PF, AR | Cochrane Risk of Bias tool |
| Williams 2018[254] | Rheumatoid arthritis | 7 | 841 | Strengthening and ROM home exercise, mobility exercises, co-ordination exercises | Waitlist, no-treatment, advice | P, PF, AR | Cochrane Risk of Bias tool |
| Sobue 2022[255] | Rheumatoid arthritis | 9 | 1343 | Dynamic exercise, Progressive resistance training, water-based aerobic exercise | Conventional joint rehabilitation and control | P, PF, MH | Cochrane Risk of Bias tool |
| Wu 2023[256] | Rheumatoid arthritis | 9 | 351 | Tai chi | Education, usual physical activities without exercises | P, PF | Cochrane Risk of Bias tool |
| Ye 2022[257] | Rheumatoid arthritis | 13 | 967 | Walking, cycling and jogging | Education, usual care, exercise therapy | P, PF | Cochrane Risk of Bias tool |
| Shoulder pain |  |  |  |  |  |  |  |
| Gutierrez-Espinoza 2020[258] | Subacromial impingement syndrome | 4 | 371 | Home exercise program | Supervised physiotherapy | P, PF | Cochrane Risk of Bias tool |
| Desmeules 2015[259] | Rotator cuff tendinopathy | 2 | 60 | Exercises | Ultrasound therapy | PF | Cochrane Risk of Bias tool |
| Dong 2015[260] | Shoulder impingement syndrome | 33 | 2300 | Exercise | Acupuncture therapy, routine exercise treatment, kinesio taping therapy, low-level laser therapy, manual therapy, microwave diathermy therapy, no treatment/placebo, pulsed electromagnetic field therapy, radial extracorporeal shockwave therapy, specific exercise therapy, ultrasound therapy | P, PF | Cochrane Risk of Bias tool |
| Naunton 2020[261] | Rotator cuff shoulder pain | 7 | 468 | Progressive and resisted exercise, Non-progressive or non-resisted exercise | Placebo, no treatment, ultrasound | P, PF | Cochrane Risk of Bias tool |
| Hanratty 2012[262] | Subacromial impingement syndrome | 6 | 1162 | Flexibility, strengthening exercise | Manual therapy and exercise, conservative interventions | P, PF, MH | Cochrane Risk of Bias tool |
| Mertens 2021[263] | Frozen shoulder | 19 | 524 | Isometric or strengthening exercises, pendulum exercises, and stretching exercises | Multimodal programme | P, PF | Cochrane Risk of Bias tool |
| Larsson 2019[264] | Subacromial impingement syndrome | 7 | 303 | Resistance exercise | Resistance exercise (concentric or concentric/eccentric), pain-free eccentric exercise, mobility exercises | P, PF | PEDro |
| Liaghat 2021[265] | Subacromial pain syndrome and supraspinatus tendinosis | 10 | 597 | Supervised training | No training or self-training | P, PF | Cochrane Risk of Bias tool |
| Brudvig 2011[266] | Shoulder dysfunction | 7 | 290 | ROM, stretching, strengthening, and neuromuscular control exercises and dynamic stability training | Mobilization techniques | P, PF | Joy Macdermid's scale |
| Marinko 2011[267] | Shoulder pain | 11 | NR | Therapeutic exercises | Alternative or no intervention | P, PF | PEDro |
| Shire 2017[268] | Subacromial impingement syndrome | 5 | 231 | Specific proprioceptive exercises and centering of the humeral head (positioning), scapular specific exercises, specific scapular stabilization and neuromuscular control exercises, rotator cuff exercises | General resistance exercises | P, PF | Cochrane Risk of Bias tool |
| Sharma 2021[269] | Shoulder impingement syndrome | 7 | 597 | Exercise | Manual therapy plus exercise | P, PF | PEDro |
| Picón 2021[270] | Shoulder pain | 7 | 3292 | Motor control training, resistance training, dynamic strength training | Education or no treatment | P | PEDro |
| Steuri 2017[271] | Shoulder impingement | 177 | 10529 | Exercise therapy | Non-specific exercise or no treatment | P, PF | Cochrane Risk of Bias tool |
| Babatunde 2021[272] | Subacromial shoulder conditions | 99 | 6764 | Therapeutic exercises | Manual therapy, control, electrotherapy | P, PF | Cochrane Risk of Bias tool |
| Celik 2020[273] | Shoulder disorders | 14 | 680 | Exercise | Kinesio taping | P, PF | PEDro |
| Liu 2022[274] | Non-specific shoulder pain | 10 | 935 | Range of motion, isometric, strengthening and scapular stabilizing exercises | Thrust manipulation, cryotherapy, mobilization, electrophysical agents, no treatment | P, PF | Cochrane Risk of Bias tool |

Abbreviations: P, pain; PF, physical function; MH, mental health; AE, adverse events; AR, adherence rate; PEDro, physiotherapy evidence database; SIGN, Scottish Intercollegiate Guidelines Network guidelines; NR, not reported; OA, osteoarthritis; RA, rheumatoid arthritis; LBP, low back pain; WBV, whole body vibration; PAT, progressive aerobic training; PRT, progressive resistance training; ROM, range of motion; TENS, transcutaneous electrical nerve stimulator; PNF, proprioceptive neuromuscular facilitation, PFPS, patellofemoral pain syndrome; ROB, risk of bias**.**

# Supplementary file 6. References list of included studies

1. Murphy MC, Travers MJ, Chivers P, Debenham JR, Docking SI, Rio EK, et al. Efficacy of heavy eccentric calf training for treating mid-portion Achilles tendinopathy: a systematic review and meta-analysis. Br J Sports Med. 2019;53:1070–7.

2. Siriphorn A, Eksakulkla S. Calf stretching and plantar fascia-specific stretching for plantar fasciitis: A systematic review and meta-analysis. J Bodyw Mov Ther. 2020;24:222–32.

3. Sussmilch-Leitch SP, Collins NJ, Bialocerkowski AE, Warden SJ, Crossley KM. Physical therapies for Achilles tendinopathy: systematic review and meta-analysis. J Foot Ankle Res. 2012;5:15.

4. Wilson F, Walshe M, O’Dwyer T, Bennett K, Mockler D, Bleakley C. Exercise, orthoses and splinting for treating Achilles tendinopathy: a systematic review with meta-analysis. Br J Sports Med. 2018;52:1564–74.

5. Arora NK, Sharma S, Arora IK. Physical modalities with eccentric exercise are no better than eccentric exercise alone in the treatment of chronic achilles tendinopathy: A systematic review and meta-analysis. Foot (Edinb). 2022;53:101927.

6. Prudencio D.A., Maffulli N., Migliorini F., Serafim T.T., Nunes L.F., Sanada L.S., et al. Eccentric exercise is more effective than other exercises in the treatment of mid-portion Achilles tendinopathy: systematic review and meta-analysis. BMC Sports Science, Medicine and Rehabilitation. 2023;15:9.

7. Bidonde J, Busch A, Schachter C, Webber S, Musselman K, Overend T, et al. Mixed exercise training for adults with fibromyalgia. Cochrane Database of Systematic Reviews [Internet]. 2019; Available from: http://dx.doi.org/10.1002/14651858.CD013340

8. Busch AJ, Webber SC, Richards RS, Bidonde J, Schachter CL, Schafer LA, et al. Resistance exercise training for fibromyalgia. Cochrane Database Syst Rev. 2013;2013:CD010884.

9. Cheng CA, Chiu YW, Wu D, Kuan YC, Chen SN, Tam KW. Effectiveness of Tai Chi on fibromyalgia patients: A meta-analysis of randomized controlled trials. Complement Ther Med. 2019;46:1–8.

10. Guzmán-Pavón MJ, Cavero-Redondo I, Martínez-Vizcaíno V, Fernández-Rodríguez R, Reina-Gutierrez S, Álvarez-Bueno C. Effect of Physical Exercise Programs on Myofascial Trigger Points-Related Dysfunctions: A Systematic Review and Meta-analysis. Pain Med. 2020;21:2986–96.

11. Busch AJ, Barber KA, Overend TJ, Peloso PM, Schachter CL. Exercise for treating fibromyalgia syndrome. Cochrane Database Syst Rev. 2007;CD003786.

12. Langhorst J, Klose P, Dobos GJ, Bernardy K, Häuser W. Efficacy and safety of meditative movement therapies in fibromyalgia syndrome: a systematic review and meta-analysis of randomized controlled trials. Rheumatol Int. 2013;33:193–207.

13. Mata Diz JB, de Souza JR, Leopoldino AA, Oliveira VC. Exercise, especially combined stretching and strengthening exercise, reduces myofascial pain: a systematic review. J Physiother. 2017;63:17–22.

14. Sosa-Reina MD, Nunez-Nagy S, Gallego-Izquierdo T, Pecos-Martín D, Monserrat J, Álvarez-Mon M. Effectiveness of Therapeutic Exercise in Fibromyalgia Syndrome: A Systematic Review and Meta-Analysis of Randomized Clinical Trials. Biomed Res Int. 2017;2017:2356346.

15. Bidonde J, Busch AJ, Schachter CL, Overend TJ, Kim SY, Góes SM, et al. Aerobic exercise training for adults with fibromyalgia. Cochrane Database Syst Rev. 2017;6:CD012700.

16. Galvão-Moreira LV, de Castro LO, Moura ECR, de Oliveira CMB, Nogueira Neto J, Gomes LMRS, et al. Pool-based exercise for amelioration of pain in adults with fibromyalgia syndrome: A systematic review and meta-analysis. Mod Rheumatol. 2021;31:904–11.

17. Bidonde J, Busch AJ, Webber SC, Schachter CL, Danyliw A, Overend TJ, et al. Aquatic exercise training for fibromyalgia. Cochrane Database Syst Rev. 2014;CD011336.

18. McDowell CP, Cook DB, Herring MP. The Effects of Exercise Training on Anxiety in Fibromyalgia Patients: A Meta-analysis. Med Sci Sports Exerc. 2017;49:1868–76.

19. Kim S, Busch A, Overend T, Schachter C, van der Spuy I, Boden C, et al. Flexibility exercise training for adults with fibromyalgia. Cochrane Database of Systematic Reviews [Internet]. 2019; Available from: http://dx.doi.org/10.1002/14651858.CD013419

20. Bravo C, Skjaerven LH, Guitard Sein-Echaluce L, Catalan-Matamoros D. Effectiveness of movement and body awareness therapies in patients with fibromyalgia: a systematic review and meta-analysis. Eur J Phys Rehabil Med. 2019;55:646–57.

21. Zhang K.-D., Wang L.-Y., Zhang Z.-H., Zhang D.-X., Lin X.-W., Meng T., et al. Effect of Exercise Interventions on Health-Related Quality of Life in Patients with Fibromyalgia Syndrome: A Systematic Review and Network Meta-Analysis. Journal of Pain Research. 2022;15:3639–56.

22. Couto N, Monteiro D, Cid L, Bento T. Effect of different types of exercise in adult subjects with fibromyalgia: a systematic review and meta-analysis of randomised clinical trials. Sci Rep. 2022;12:10391.

23. Vilarino GT, Branco JHL, de Souza LC, Andrade A. Effects of resistance training on the physical symptoms and functional capacity of patients with fibromyalgia: a systematic review and meta-analysis of randomized clinical trials. Ir J Med Sci. 2022;

24. Murillo-Garcia A, Adsuar JC, Villafaina S, Collado-Mateo D, Gusi N. Creative versus repetitive dance therapies to reduce the impact of fibromyalgia and pain: A systematic review and meta-analysis. Complement Ther Clin Pract. 2022;47:101577.

25. Kundakci B, Kaur J, Goh SL, Hall M, Doherty M, Zhang W, et al. Efficacy of nonpharmacological interventions for individual features of fibromyalgia: a systematic review and meta-analysis of randomised controlled trials. Pain. 2022;163:1432–45.

26. Albuquerque M.L.L., Monteiro D., Marinho D.A., Vilarino G.T., Andrade A., Neiva H.P. Effects of different protocols of physical exercise on fibromyalgia syndrome treatment: systematic review and meta-analysis of randomized controlled trials. Rheumatology International. 2022;42:1893–908.

27. Wu J, Chen Z, Zheng K, Huang W, Liu F, Lin J, et al. Benefits of Exergame Training for Female Patients With Fibromyalgia: A Systematic Review and Meta-Analysis of Randomized Controlled Trials. Arch Phys Med Rehabil. 2022;103:1192-1200.e2.

28. Ma J, Zhang T, Li X, Chen X, Zhao Q. Effects of aquatic physical therapy on clinical symptoms, physical function, and quality of life in patients with fibromyalgia: A systematic review and meta-analysis. Physiother Theory Pract. 2022;1–19.

29. Barker AL, Talevski J, Morello RT, Brand CA, Rahmann AE, Urquhart DM. Effectiveness of aquatic exercise for musculoskeletal conditions: a meta-analysis. Arch Phys Med Rehabil. 2014;95:1776–86.

30. Brosseau L, Pelland L, Wells G, Macleay L, Lamothe C, Michaud G, et al. Efficacy of Aerobic Exercises For Osteoarthritis (part II): A Meta-analysis. Physical Therapy Reviews. 2004;9:125–45.

31. Collado-Mateo D, Merellano-Navarro E, Olivares PR, García-Rubio J, Gusi N. Effect of exergames on musculoskeletal pain: A systematic review and meta-analysis. Scand J Med Sci Sports. 2018;28:760–71.

32. Dong Y, Wang W, Zheng J, Chen S, Qiao J, Wang X. Whole Body Vibration Exercise for Chronic Musculoskeletal Pain: A Systematic Review and Meta-analysis of Randomized Controlled Trials. Arch Phys Med Rehabil. 2019;100:2167–78.

33. Hall J, Swinkels A, Briddon J, McCabe CS. Does aquatic exercise relieve pain in adults with neurologic or musculoskeletal disease? A systematic review and meta-analysis of randomized controlled trials. Arch Phys Med Rehabil. 2008;89:873–83.

34. Hall A, Maher C, Latimer J, Ferreira M. The effectiveness of Tai Chi for chronic musculoskeletal pain conditions: a systematic review and meta-analysis. Arthritis Rheum. 2009;61:717–24.

35. Lafrance S, Ouellet P, Alaoui R, Roy JS, Lewis J, Christiansen DH, et al. Motor Control Exercises Compared to Strengthening Exercises for Upper- and Lower-Extremity Musculoskeletal Disorders: A Systematic Review With Meta-Analyses of Randomized Controlled Trials. Phys Ther. 2021;101.

36. Miller CT, Owen PJ, Than CA, Ball J, Sadler K, Piedimonte A, et al. Attempting to Separate Placebo Effects from Exercise in Chronic Pain: A Systematic Review and Meta-analysis. Sports Med. 2021;

37. Ouellet P, Lafrance S, Pizzi A, Roy J-S, Lewis J, Christiansen DH, et al. Region-specific Exercises vs General Exercises in the Management of Spinal and Peripheral Musculoskeletal Disorders: A Systematic Review With Meta-analyses of Randomized Controlled Trials. Arch Phys Med Rehabil. 2021;102:2201–18.

38. Polaski AM, Phelps AL, Kostek MC, Szucs KA, Kolber BJ. Exercise-induced hypoalgesia: A meta-analysis of exercise dosing for the treatment of chronic pain. PLoS One. 2019;14:e0210418.

39. Schäfer AGM, Zalpour C, von Piekartz H, Hall TM, Paelke V. The Efficacy of Electronic Health-Supported Home Exercise Interventions for Patients With Osteoarthritis of the Knee: Systematic Review. J Med Internet Res. 2018;20:e152.

40. Sieczkowska SM, Coimbra DR, Vilarino GT, Andrade A. Effects of resistance training on the health-related quality of life of patients with rheumatic diseases: Systematic review with meta-analysis and meta-regression. Semin Arthritis Rheum. 2020;50:342–53.

41. Waller B, Ogonowska-Slodownik A, Vitor M, Lambeck J, Daly D, Kujala UM, et al. Effect of therapeutic aquatic exercise on symptoms and function associated with lower limb osteoarthritis: systematic review with meta-analysis. Phys Ther. 2014;94:1383–95.

42. Yan JH, Gu WJ, Sun J, Zhang WX, Li BW, Pan L. Efficacy of Tai Chi on pain, stiffness and function in patients with osteoarthritis: a meta-analysis. PLoS One. 2013;8:e61672.

43. Batterham SI, Heywood S, Keating JL. Systematic review and meta-analysis comparing land and aquatic exercise for people with hip or knee arthritis on function, mobility and other health outcomes. BMC Musculoskelet Disord. 2011;12:123.

44. Kong LJ, Lauche R, Klose P, Bu JH, Yang XC, Guo CQ, et al. Tai Chi for Chronic Pain Conditions: A Systematic Review and Meta-analysis of Randomized Controlled Trials. Sci Rep. 2016;6:25325.

45. Denham-Jones L, Gaskell L, Spence N, Pigott T. A systematic review of the effectiveness of Pilates on pain, disability, physical function, and quality of life in older adults with chronic musculoskeletal conditions. Musculoskeletal Care. 2021;

46. Zhang Q, Young L, Li F. Network Meta-Analysis of Various Nonpharmacological Interventions on Pain Relief in Older Adults With Osteoarthritis. Am J Phys Med Rehabil. 2019;98:469–78.

47. Hurley M, Dickson K, Hallett R, Grant R, Hauari H, Walsh N, et al. Exercise interventions and patient beliefs for people with hip, knee or hip and knee osteoarthritis: a mixed methods review. Cochrane Database Syst Rev. 2018;4:CD010842.

48. Lauche R, Hunter DJ, Adams J, Cramer H. Yoga for Osteoarthritis: a Systematic Review and Meta-analysis. Curr Rheumatol Rep. 2019;21:47.

49. Zampogna B, Papalia R, Papalia GF, Campi S, Vasta S, Vorini F, et al. The Role of Physical Activity as Conservative Treatment for Hip and Knee Osteoarthritis in Older People: A Systematic Review and Meta-Analysis. J Clin Med. 2020;9.

50. Regnaux J, Lefevre‐Colau M, Trinquart L, Nguyen C, Boutron I, Brosseau L, et al. High‐intensity versus low‐intensity physical activity or exercise in people with hip or knee osteoarthritis. Cochrane Database of Systematic Reviews [Internet]. 2015; Available from: http://dx.doi.org/10.1002/14651858.CD010203.pub2

51. Siddall B, Ram A, Jones MD, Booth J, Perriman D, Summers SJ. Short-term impact of combining pain neuroscience education with exercise for chronic musculoskeletal pain: a systematic review and meta-analysis. Pain. 2021;

52. Denham-Jones L, Gaskell L, Spence N, Tim Pigott. A systematic review of the effectiveness of yoga on pain, physical function, and quality of life in older adults with chronic musculoskeletal conditions. Musculoskeletal Care. 2021;

53. Smith BE, Hendrick P, Smith TO, Bateman M, Moffatt F, Rathleff MS, et al. Should exercises be painful in the management of chronic musculoskeletal pain? A systematic review and meta-analysis. Br J Sports Med. 2017;51:1679–87.

54. Ferro Moura Franco K, Lenoir D, Dos Santos Franco YR, Jandre Reis FJ, Nunes Cabral CM, Meeus M. Prescription of exercises for the treatment of chronic pain along the continuum of nociplastic pain: A systematic review with meta-analysis. Eur J Pain. 2021;25:51–70.

55. O’Connor SR, Tully MA, Ryan B, Bleakley CM, Baxter GD, Bradley JM, et al. Walking exercise for chronic musculoskeletal pain: systematic review and meta-analysis. Arch Phys Med Rehabil. 2015;96:724-734.e3.

56. Fransen M, McConnell S, Bell M. Therapeutic exercise for people with osteoarthritis of the hip or knee. A systematic review. J Rheumatol. 2002;29:1737–45.

57. Bartels EM, Juhl CB, Christensen R, Hagen KB, Danneskiold-Samsøe B, Dagfinrud H, et al. Aquatic exercise for the treatment of knee and hip osteoarthritis. Cochrane Database Syst Rev. 2016;3:CD005523.

58. Goh SL, Persson MSM, Stocks J, Hou Y, Lin J, Hall MC, et al. Efficacy and potential determinants of exercise therapy in knee and hip osteoarthritis: A systematic review and meta-analysis. Ann Phys Rehabil Med. 2019;62:356–65.

59. Verhagen AP, Bierma-Zeinstra SMA, Burdorf A, Stynes SM, de Vet HCW, Koes BW. Conservative interventions for treating work-related complaints of the arm, neck or shoulder in adults. Cochrane Database Syst Rev. 2013;CD008742.

60. Wang Y, Lu S, Wang R, Jiang P, Rao F, Wang B, et al. Integrative effect of yoga practice in patients with knee arthritis: A PRISMA-compliant meta-analysis. Medicine. 2018;97:e11742.

61. Escalante Y, Saavedra JM, García-Hermoso A, Silva AJ, Barbosa TM. Physical exercise and reduction of pain in adults with lower limb osteoarthritis: a systematic review. J Back Musculoskelet Rehabil. 2010;23:175–86.

62. Fernandopulle S, Perry M, Manlapaz D, Jayakaran P. Effect of Land-Based Generic Physical Activity Interventions on Pain, Physical Function, and Physical Performance in Hip and Knee Osteoarthritis: A Systematic Review and Meta-Analysis. Am J Phys Med Rehabil. 2017;96:773–92.

63. Ferreira PH, Ferreira ML, Maher CG, Herbert RD, Refshauge K. Specific stabilisation exercise for spinal and pelvic pain: a systematic review. Aust J Physiother. 2006;52:79–88.

64. Skelly AC, Chou R, Dettori JR, Turner JA, Friedly JL, Rundell SD, et al. Noninvasive Nonpharmacological Treatment for Chronic Pain: A Systematic Review Update [Internet]. Rockville (MD): Agency for Healthcare Research and Quality (US); 2020 [cited 2022 Aug 23]. Available from: http://www.ncbi.nlm.nih.gov/books/NBK556229/

65. Weng Q, Goh SL, Wu J, Persson MSM, Wei J, Sarmanova A, et al. Comparative efficacy of exercise therapy and oral non-steroidal anti-inflammatory drugs and paracetamol for knee or hip osteoarthritis: a network meta-analysis of randomised controlled trials. Br J Sports Med. 2023;

66. Wen Y.-R., Shi J., Wang Y.-F., Lin Y.-Y., Hu Z.-Y., Lin Y.-T., et al. Are Mind-Body Exercise Beneficial for Treating Pain, Function, and Quality of Life in Middle-Aged and Old People With Chronic Pain? A Systematic Review and Meta-Analysis. Frontiers in Aging Neuroscience. 2022;14:921069.

67. Amiri S. Exercise training and depression and anxiety in musculoskeletal pain patients: a meta-analysis of randomized control trials. Neuropsychiatrie [Internet]. 2022; Available from: http://www.springerlink.com/content/0948-6259/

68. Thompson D, Rattu S, Tower J, Egerton T, Francis J, Merolli M. Mobile app use to support therapeutic exercise for musculoskeletal pain conditions may help improve pain intensity and self-reported physical function: a systematic review. J Physiother. 2023;69:23–34.

69. Thorlund JB, Simic M, Pihl K, Berthelsen DB, Day R, Koes B, et al. Similar Effects of Exercise Therapy, Nonsteroidal Anti-inflammatory Drugs, and Opioids for Knee Osteoarthritis Pain: A Systematic Review with Network Meta-analysis. J Orthop Sports Phys Ther. 2022;52:207–16.

70. French H, Abbott J, Galvin R. Adjunctive therapies in addition to land‐based exercise therapy for osteoarthritis of the hip or knee. Cochrane Database of Systematic Reviews [Internet]. 2022; Available from: http://dx.doi.org/10.1002/14651858.CD011915.pub2

71. Duan X, Wei W, Zhou P, Liu X, Yu J, Xu Y, et al. Effectiveness of aquatic exercise in lower limb osteoarthritis: a meta-analysis of randomized controlled trials. Int J Rehabil Res. 2022;45:126–36.

72. Song JA, Oh JW. Effects of Aquatic Exercises for Patients with Osteoarthritis: Systematic Review with Meta-Analysis. Healthcare (Basel). 2022;10.

73. Ortega-Castillo M, Cuesta-Vargas A, Luque-Teba A, Trinidad-Fernández M. The role of progressive, therapeutic exercise in the management of upper limb tendinopathies: A systematic review and meta-analysis. Musculoskelet Sci Pract. 2022;62:102645.

74. Cuenca-Martinez F., Sempere-Rubio N., Varangot-Reille C., Fernandez-Carnero J., Suso-Marti L., Alba-Quesada P., et al. Effects of High-Intensity Interval Training (HIIT) on Patients with Musculoskeletal Disorders: A Systematic Review and Meta-Analysis with a Meta-Regression and Mapping Report. Diagnostics. 2022;12:2532.

75. de F. Silva A., Maia L.B., Mendonca V.A., dos Santos J.M., Coelho-Oliveira A.C., Santos J.N.V., et al. Efficacy of Hip Strengthening on Pain Intensity, Disability, and Strength in Musculoskeletal Conditions of the Trunk and Lower Limbs: A Systematic Review with Meta-Analysis and Grade Recommendations. Diagnostics. 2022;12:2910.

76. Runge N, Aina A, May S. The Benefits of Adding Manual Therapy to Exercise Therapy for Improving Pain and Function in Patients With Knee or Hip Osteoarthritis: A Systematic Review With Meta-analysis. J Orthop Sports Phys Ther. 2022;52:675-A13.

77. Karanasios S, Korakakis V, Whiteley R, Vasilogeorgis I, Woodbridge S, Gioftsos G. Exercise interventions in lateral elbow tendinopathy have better outcomes than passive interventions, but the effects are small: a systematic review and meta-analysis of 2123 subjects in 30 trials. Br J Sports Med. 2021;55:477–85.

78. Yoon SY, Kim YW, Shin IS, Kang S, Moon HI, Lee SC. The Beneficial Effects of Eccentric Exercise in the Management of Lateral Elbow Tendinopathy: A Systematic Review and Meta-Analysis. J Clin Med. 2021;10.

79. Wood SM, Yoon AP, Tseng HJ, Yang LY, Chung KC. Comparative Effectiveness of Physical Therapy and Electrophysiotherapy for the Treatment of Lateral Epicondylitis: A Network Meta-Analysis. Plast Reconstr Surg. 2022;150:594e–607e.

80. Magni NE, McNair PJ, Rice DA. The effects of resistance training on muscle strength, joint pain, and hand function in individuals with hand osteoarthritis: a systematic review and meta-analysis. Arthritis Res Ther. 2017;19:131.

81. Østerås N, Kjeken I, Smedslund G, Moe RH, Slatkowsky-Christensen B, Uhlig T, et al. Exercise for hand osteoarthritis. Cochrane Database Syst Rev. 2017;1:CD010388.

82. Bertozzi L, Valdes K, Vanti C, Negrini S, Pillastrini P, Villafañe JH. Investigation of the effect of conservative interventions in thumb carpometacarpal osteoarthritis: systematic review and meta-analysis. Disabil Rehabil. 2015;37:2025–43.

83. Fransen M, McConnell S, Hernandez-Molina G, Reichenbach S. Does land-based exercise reduce pain and disability associated with hip osteoarthritis? A meta-analysis of randomized controlled trials. Osteoarthritis Cartilage. 2010;18:613–20.

84. Fransen M, McConnell S, Hernandez‐Molina G, Reichenbach S. Exercise for osteoarthritis of the hip. Cochrane Database of Systematic Reviews [Internet]. 2014; Available from: http://dx.doi.org/10.1002/14651858.CD007912.pub2

85. Hansen S, Mikkelsen LR, Overgaard S, Mechlenburg I. Effectiveness of supervised resistance training for patients with hip osteoarthritis - a systematic review. Dan Med J. 2020;67.

86. Moseng T, Dagfinrud H, Smedslund G, Østerås N. The importance of dose in land-based supervised exercise for people with hip osteoarthritis. A systematic review and meta-analysis. Osteoarthritis Cartilage. 2017;25:1563–76.

87. Sampath KK, Mani R, Miyamori T, Tumilty S. The effects of manual therapy or exercise therapy or both in people with hip osteoarthritis: a systematic review and meta-analysis. Clin Rehabil. 2016;30:1141–55.

88. Hernández-Molina G, Reichenbach S, Zhang B, LaValley M, Felson DT. Effect of Therapeutic Exercise for Hip Osteoarthritis Pain: Results of a Meta-Analysis. Arthritis Rheum. 2008;59:1221–8.

89. Beumer L, Wong J, Warden SJ, Kemp JL, Foster P, Crossley KM. Effects of exercise and manual therapy on pain associated with hip osteoarthritis: a systematic review and meta-analysis. Br J Sports Med. 2016;50:458–63.

90. Corbett MS, Rice SJ, Madurasinghe V, Slack R, Fayter DA, Harden M, et al. Acupuncture and other physical treatments for the relief of pain due to osteoarthritis of the knee: network meta-analysis. Osteoarthritis Cartilage. 2013;21:1290–8.

91. Tanaka R, Ozawa J, Kito N, Moriyama H. Effect of the Frequency and Duration of Land-based Therapeutic Exercise on Pain Relief for People with Knee Osteoarthritis: A Systematic Review and Meta-analysis of Randomized Controlled Trials. J Phys Ther Sci. 2014;26:969–75.

92. Lauche R, Langhorst J, Dobos G, Cramer H. A systematic review and meta-analysis of Tai Chi for osteoarthritis of the knee. Complement Ther Med. 2013;21:396–406.

93. Bhaskar Raj N, Rao USM, Saha S, Saha S. Impact of Isokinetic training on pain in patients’ with Knee osteoarthritis-A Meta-analysis. Research Journal of Pharmacy and Technology. 2018;11:3402–10.

94. NASCIMENTO LR, TEIXEIRA-SALMELA LF, SOUZA RB, RESENDE RA. Hip and Knee Strengthening Is More Effective Than Knee Strengthening Alone for Reducing Pain and Improving Activity in Individuals With Patellofemoral Pain: A Systematic Review With Meta-analysis. Journal of Orthopaedic & Sports Physical Therapy. 2018;48:19–31.

95. Tanaka R, Ozawa J, Kito N, Moriyama H. Efficacy of strengthening or aerobic exercise on pain relief in people with knee osteoarthritis: a systematic review and meta-analysis of randomized controlled trials. Clin Rehabil. 2013;27:1059–71.

96. Wang SY, Olson-Kellogg B, Shamliyan TA, Choi JY, Ramakrishnan R, Kane RL. Physical therapy interventions for knee pain secondary to osteoarthritis: a systematic review. Ann Intern Med. 2012;157:632–44.

97. Clijsen R, Fuchs J, Taeymans J. Effectiveness of exercise therapy in treatment of patients with patellofemoral pain syndrome: systematic review and meta-analysis. Phys Ther. 2014;94:1697–708.

98. Li S, Shaharudin S, Abdul Kadir MR. Effects of Blood Flow Restriction Training on Muscle Strength and Pain in Patients With Knee Injuries: A Meta-Analysis. Am J Phys Med Rehabil. 2021;100:337–44.

99. Imoto AM, Pardo JP, Brosseau L, Taki J, Desjardins B, Thevenot O, et al. Evidence synthesis of types and intensity of therapeutic land-based exercises to reduce pain in individuals with knee osteoarthritis. Rheumatol Int. 2019;39:1159–79.

100. Jeong HS, Lee SC, Jee H, Song JB, Chang HS, Lee SY. Proprioceptive Training and Outcomes of Patients With Knee Osteoarthritis: A Meta-Analysis of Randomized Controlled Trials. J Athl Train. 2019;54:418–28.

101. Li Y, Su Y, Chen S, Zhang Y, Zhang Z, Liu C, et al. The effects of resistance exercise in patients with knee osteoarthritis: a systematic review and meta-analysis. Clin Rehabil. 2016;30:947–59.

102. Grantham B, Korakakis V, O’Sullivan K. Does blood flow restriction training enhance clinical outcomes in knee osteoarthritis: A systematic review and meta-analysis. Phys Ther Sport. 2021;49:37–49.

103. Coudeyre E, Jegu AG, Giustanini M, Marrel JP, Edouard P, Pereira B. Isokinetic muscle strengthening for knee osteoarthritis: A systematic review of randomized controlled trials with meta-analysis. Ann Phys Rehabil Med. 2016;59:207–15.

104. Hu L, Wang Y, Liu X, Ji X, Ma Y, Man S, et al. Tai Chi exercise can ameliorate physical and mental health of patients with knee osteoarthritis: systematic review and meta-analysis. Clin Rehabil. 2021;35:64–79.

105. Jansen MJ, Viechtbauer W, Lenssen AF, Hendriks EJ, de Bie RA. Strength training alone, exercise therapy alone, and exercise therapy with passive manual mobilisation each reduce pain and disability in people with knee osteoarthritis: a systematic review. J Physiother. 2011;57:11–20.

106. Smith TO, King JJ, Hing CB. The effectiveness of proprioceptive-based exercise for osteoarthritis of the knee: a systematic review and meta-analysis. Rheumatol Int. 2012;32:3339–51.

107. Tanaka R, Ozawa J, Kito N, Yamasaki T, Moriyama H. Evidence of Improvement in Various Impairments by Exercise Interventions in Patients with Knee Osteoarthritis: A Systematic Review and Meta-analysis of Randomized Clinical Trials. J Jpn Phys Ther Assoc. 2013;16:7–21.

108. Dong R, Wu Y, Xu S, Zhang L, Ying J, Jin H, et al. Is aquatic exercise more effective than land-based exercise for knee osteoarthritis? Medicine (Baltimore). 2018;97:e13823.

109. Anwer S, Alghadir A, Brismée JM. Effect of Home Exercise Program in Patients With Knee Osteoarthritis: A Systematic Review and Meta-analysis. J Geriatr Phys Ther. 2016;39:38–48.

110. Cuyul-Vásquez I, Leiva-Sepúlveda A, Catalán-Medalla O, Araya-Quintanilla F, Gutiérrez-Espinoza H. The addition of blood flow restriction to resistance exercise in individuals with knee pain: a systematic review and meta-analysis. Braz J Phys Ther. 2020;24:465–78.

111. Fransen M, McConnell S, Harmer A, Van der Esch M, Simic M, Bennell K. Exercise for osteoarthritis of the knee. Cochrane Database of Systematic Reviews [Internet]. 2015; Available from: http://dx.doi.org/10.1002/14651858.CD004376.pub3

112. Manojlović D, Kozinc Ž, Šarabon N. Trunk, Hip and Knee Exercise Programs for Pain Relief, Functional Performance and Muscle Strength in Patellofemoral Pain: Systematic Review and Meta-Analysis. J Pain Res. 2021;14:1431–49.

113. Lu M, Su Y, Zhang Y, Zhang Z, Wang W, He Z, et al. Effectiveness of aquatic exercise for treatment of knee osteoarthritis: Systematic review and meta-analysis. Z Rheumatol. 2015;74:543–52.

114. Luan L, Bousie J, Pranata A, Adams R, Han J. Stationary cycling exercise for knee osteoarthritis: A systematic review and meta-analysis. Clin Rehabil. 2021;35:522–33.

115. Bartholdy C, Juhl C, Christensen R, Lund H, Zhang W, Henriksen M. The role of muscle strengthening in exercise therapy for knee osteoarthritis: A systematic review and meta-regression analysis of randomized trials. Semin Arthritis Rheum. 2017;47:9–21.

116. Rogan S, Haehni M, Luijckx E, Dealer J, Reuteler S, Taeymans J. Effects of Hip Abductor Muscles Exercises on Pain and Function in Patients With Patellofemoral Pain: A Systematic Review and Meta-Analysis. J Strength Cond Res. 2019;33:3174–87.

117. van der Heijden R, Lankhorst N, van Linschoten R, Bierma‐Zeinstra S, van Middelkoop M. Exercise for treating patellofemoral pain syndrome. Cochrane Database of Systematic Reviews [Internet]. 2015; Available from: http://dx.doi.org/10.1002/14651858.CD010387.pub2

118. Newberry SJ, FitzGerald J, SooHoo NF, Booth M, Marks J, Motala A, et al. Treatment of Osteoarthritis of the Knee: An Update Review [Internet]. Rockville (MD): Agency for Healthcare Research and Quality (US); 2017 [cited 2022 Aug 9]. Available from: http://www.ncbi.nlm.nih.gov/books/NBK447543/

119. Li X, Wang XQ, Chen BL, Huang LY, Liu Y. Whole-Body Vibration Exercise for Knee Osteoarthritis: A Systematic Review and Meta-Analysis. Evid Based Complement Alternat Med. 2015;2015:758147.

120. Roddy E, Zhang W, Doherty M. Aerobic walking or strengthening exercise for osteoarthritis of the knee? A systematic review. Ann Rheum Dis. 2005;64:544–8.

121. Winters M, Holden S, Lura CB, Welton NJ, Caldwell DM, Vicenzino BT, et al. Comparative effectiveness of treatments for patellofemoral pain: a living systematic review with network meta-analysis. Br J Sports Med. 2020;55:369–77.

122. Hislop AC, Collins NJ, Tucker K, Deasy M, Semciw AI. Does adding hip exercises to quadriceps exercises result in superior outcomes in pain, function and quality of life for people with knee osteoarthritis? A systematic review and meta-analysis. Br J Sports Med. 2020;54:263–71.

123. Devos-Comby L, Cronan T, Roesch SC. Do exercise and self-management interventions benefit patients with osteoarthritis of the knee? A metaanalytic review. J Rheumatol. 2006;33:744–56.

124. Li R, Chen H, Feng J, Xiao Y, Zhang H, Lam CW, et al. Effectiveness of Traditional Chinese Exercise for Symptoms of Knee Osteoarthritis: A Systematic Review and Meta-Analysis of Randomized Controlled Trials. Int J Environ Res Public Health. 2020;17.

125. Zeng ZP, Liu YB, Fang J, Liu Y, Luo J, Yang M. Effects of Baduanjin exercise for knee osteoarthritis: A systematic review and meta-analysis. Complement Ther Med. 2020;48:102279.

126. Anwer S, Alghadir A, Zafar H, Brismée JM. Effects of orthopaedic manual therapy in knee osteoarthritis: a systematic review and meta-analysis. Physiotherapy. 2018;104:264–76.

127. Cardoso RK, Caputo EL, Rombaldi AJ, Del Vecchio FB. Effects of strength training on the treatment of patellofemoral pain syndrome - a meta-analysis of randomized controlled trials. Fisioterapia em Movimento. 2017;30:391–8.

128. Chen T, Or CK, Chen J. Effects of technology-supported exercise programs on the knee pain, physical function, and quality of life of individuals with knee osteoarthritis and/or chronic knee pain: A systematic review and meta-analysis of randomized controlled trials. J Am Med Inform Assoc. 2021;28:414–23.

129. Wang H-N, Chen Y, Cheng L, Cai Y-H, Li W, Ni G-X. Efficacy and Safety of Blood Flow Restriction Training in Patients With Knee Osteoarthritis: A Systematic Review and Meta-Analysis. Arthritis Care Res (Hoboken). 2022;74:89–98.

130. Juhl C, Christensen R, Roos EM, Zhang W, Lund H. Impact of exercise type and dose on pain and disability in knee osteoarthritis: a systematic review and meta-regression analysis of randomized controlled trials. Arthritis Rheumatol. 2014;66:622–36.

131. Na Y, Han C, Shi Y, Zhu Y, Ren Y, Liu W. Is Isolated Hip Strengthening or Traditional Knee-Based Strengthening More Effective in Patients With Patellofemoral Pain Syndrome? A Systematic Review With Meta-analysis. Orthopaedic Journal of Sports Medicine. 2021;9:1–8.

132. Scali K, Roberts J, McFarland M, Marino K, Murray L. IS MULTI-JOINT OR SINGLE JOINT STRENGTHENING MORE EFFECTIVE IN REDUCING PAIN AND IMPROVING FUNCTION IN WOMEN WITH PATELLOFEMORAL PAIN SYNDROME? A SYSTEMATIC REVIEW AND META-ANALYSIS. Int J Sports Phys Ther. 2018;13:321–34.

133. DE OLIVEIRA SILVA D, FERRAZ PAZZINATTO M, SKOVDAL RATHLEFF M, HOLDEN S, BELL E, AZEVEDO F, et al. Patient Education for Patellofemoral Pain: A Systematic Review. Journal of Orthopaedic & Sports Physical Therapy. 2020;50:388–96.

134. Goff AJ, De Oliveira Silva D, Merolli M, Bell EC, Crossley KM, Barton CJ. Patient education improves pain and function in people with knee osteoarthritis with better effects when combined with exercise therapy: a systematic review. J Physiother. 2021;67:177–89.

135. Lack S, Barton C, Sohan O, Crossley K, Morrissey D. Proximal muscle rehabilitation is effective for patellofemoral pain: a systematic review with meta-analysis. Br J Sports Med. 2015;49:1365–76.

136. Rocha TC, Ramos PDS, Dias AG, Martins EA. The Effects of Physical Exercise on Pain Management in Patients with Knee Osteoarthritis: A Systematic Review with Metanalysis. Rev Bras Ortop (Sao Paulo). 2020;55:509–17.

137. Zhang Y, Huang L, Su Y, Zhan Z, Li Y, Lai X. The Effects of Traditional Chinese Exercise in Treating Knee Osteoarthritis: A Systematic Review and Meta-Analysis. PLoS One. 2017;12:e0170237.

138. Chen S-C, Ding S-B, Xie B-C, Tian H, Lu C-Y. Are aquatic exercises efficacious in postmenopausal women with knee osteoarthritis? A meta-analysis of randomized controlled trials. J Sports Med Phys Fitness. 2019;59:1763–70.

139. Alammari A, Spence N, Narayan A, Karnad SD, Ottayil ZC. Effect of hip abductors and lateral rotators’ muscle strengthening on pain and functional outcome in adult patients with patellofemoral pain: A systematic review and meta-analysis. J Back Musculoskelet Rehabil. 2023;36:35–60.

140. Guo X., Zhao P., Zhou X., Wang J., Wang R. A recommended exercise program appropriate for patients with knee osteoarthritis: A systematic review and meta-analysis. Frontiers in Physiology. 2022;13:934511.

141. Guo J, Peng C, Hu Z, Guo L, Dai R, Li Y. Effect of Wu Qin Xi exercises on pain and function in people with knee osteoarthritis: A systematic review and meta-analysis. Front Med (Lausanne). 2022;9:979207.

142. Wu H., Yao R., Wu J., Wen G., Wang Y. Does kinesio taping plus exercise improve pain and function in patients with knee osteoarthritis?: A systematic review and meta-analysis of randomized controlled trials. Frontiers in Physiology. 2022;13:961264.

143. Wang Y., Wu Z., Chen Z., Ye X., Chen G., Yang J., et al. Proprioceptive Training for Knee Osteoarthritis: A Systematic Review and Meta-Analysis of Randomized Controlled Trials. Frontiers in Medicine. 2021;8:699921.

144. AL-Mhanna SB, Mohamed M, Mohd Noor N, Aldhahi MI, Afolabi HA, Mutalub YB, et al. Effects of Circuit Training on Patients with Knee Osteoarthritis: A Systematic Review and Meta-Analysis. Healthcare (2227-9032). 2022;10:N.PAG-N.PAG.

145. Hua J, Sun L, Teng Y. Effects of high-intensity strength training in adults with knee osteoarthritis: a systematic review and meta-analysis of randomized controlled trials. Am J Phys Med Rehabil. 2022;

146. Challoumas D, Pedret C, Biddle M, Ng NYB, Kirwan P, Cooper B, et al. Management of patellar tendinopathy: a systematic review and network meta-analysis of randomised studies. BMJ Open Sport Exerc Med. 2021;7:e001110.

147. Pedersen JR, Sari DM, Juhl CB, Thorlund JB, Skou ST, Roos EM, et al. Variability in effect sizes of exercise therapy for knee osteoarthritis depending on comparator interventions. Ann Phys Rehabil Med. 2022;66:101708.

148. Jurado-Castro JM, Muñoz-López M, Ledesma AS, Ranchal-Sanchez A. Effectiveness of Exercise in Patients with Overweight or Obesity Suffering from Knee Osteoarthritis: A Systematic Review and Meta-Analysis. Int J Environ Res Public Health. 2022;19.

149. Neal BS, Bartholomew C, Barton CJ, Morrissey D, Lack SD. Six Treatments Have Positive Effects at 3 Months for People With Patellofemoral Pain: A Systematic Review With Meta-analysis. J Orthop Sports Phys Ther. 2022;52:750–68.

150. Xu Z, Wang Y, Zhang Y, Lu Y, Wen Y. Efficacy and safety of aquatic exercise in knee osteoarthritis: A systematic review and meta-analysis of randomized controlled trials. Clin Rehabil. 2023;37:330–47.

151. Thomas DT, R S, Prabhakar AJ, Dineshbhai PV, Eapen C. Hip abductor strengthening in patients diagnosed with knee osteoarthritis - a systematic review and meta-analysis. BMC Musculoskelet Disord. 2022;23:622.

152. Qiu CG, Chui CS, Chow SKH, Cheung WH, Wong RMY. Effects of Whole-Body Vibration Therapy on Knee Osteoarthritis: A Systematic Review and Meta-Analysis of Randomized Controlled Trials. J Rehabil Med. 2022;54:jrm00266.

153. Yang X, Yang G, Zuo Y. Whole-body vibration provides additional benefits to patients with patellofemoral pain: A protocol for systematic review and meta analysis of randomized controlled trials. Medicine. 2022;101:e31536–e31536.

154. Luan L, El-Ansary D, Adams R, Wu S, Han J. Knee osteoarthritis pain and stretching exercises: a systematic review and meta-analysis. Physiotherapy. 2022;114:16–29.

155. Amaral LKB, Souza MB, Campos MGM, Mendonça VA, Bastone A, Pereira LSM, et al. Efficacy of conservative therapy in older people with nonspecific low back pain: A systematic review with meta-analysis and GRADE recommendations. Arch Gerontol Geriatr. 2020;90:104177.

156. Hayden JA, Wilson MN, Stewart S, Cartwright JL, Smith AO, Riley RD, et al. Exercise treatment effect modifiers in persistent low back pain: an individual participant data meta-analysis of 3514 participants from 27 randomised controlled trials. Br J Sports Med. 2020;54:1277–8.

157. Hayden JA, Ellis J, Ogilvie R, Stewart SA, Bagg MK, Stanojevic S, et al. Some types of exercise are more effective than others in people with chronic low back pain: a network meta-analysis. J Physiother. 2021;67:252–62.

158. Holtzman S, Beggs RT. Yoga for chronic low back pain: a meta-analysis of randomized controlled trials. Pain Res Manag. 2013;18:267–72.

159. Jacobi S, Beynon A, Dombrowski SU, Wedderkopp N, Witherspoon R, Hébert JJ. Effectiveness of Conservative Nonpharmacologic Therapies for Pain, Disability, Physical Capacity, and Physical Activity Behavior in Patients With Degenerative Lumbar Spinal Stenosis: A Systematic Review and Meta-Analysis. Arch Phys Med Rehabil. 2021;102:2247-2260.e7.

160. Keller A, Hayden J, Bombardier C, van Tulder M. Effect sizes of non-surgical treatments of non-specific low-back pain. Eur Spine J. 2007;16:1776–88.

161. Lim EC, Poh RL, Low AY, Wong WP. Effects of Pilates-based exercises on pain and disability in individuals with persistent nonspecific low back pain: a systematic review with meta-analysis. J Orthop Sports Phys Ther. 2011;41:70–80.

162. Luomajoki HA, Bonet Beltran MB, Careddu S, Bauer CM. Effectiveness of movement control exercise on patients with non-specific low back pain and movement control impairment: A systematic review and meta-analysis. Musculoskelet Sci Pract. 2018;36:1–11.

163. Macedo L, Saragiotto B, Yamato T, Costa L, Menezes Costa L, Ostelo R, et al. Motor control exercise for acute non‐specific low back pain. Cochrane Database of Systematic Reviews [Internet]. 2016; Available from: http://dx.doi.org/10.1002/14651858.CD012085

164. Maciel RRBT, Dos Santos NC, Portella DDA, Alves PGJM, Martinez BP. Effects of physical exercise at the workplace for treatment of low back pain: a systematic review with meta-analysis. Rev Bras Med Trab. 2018;16:225–35.

165. Meng XG, Yue SW. Efficacy of aerobic exercise for treatment of chronic low back pain: a meta-analysis. Am J Phys Med Rehabil. 2015;94:358–65.

166. Miyamoto GC, Costa LO, Cabral CM. Efficacy of the Pilates method for pain and disability in patients with chronic nonspecific low back pain: a systematic review with meta-analysis. Braz J Phys Ther. 2013;17:517–32.

167. Mueller J, Niederer D. Dose-response-relationship of stabilisation exercises in patients with chronic non-specific low back pain: a systematic review with meta-regression. Sci Rep. 2020;10:16921.

168. Nduwimana I, Nindorera F, Thonnard JL, Kossi O. Effectiveness of walking versus mind-body therapies in chronic low back pain: A systematic review and meta-analysis of recent randomized controlled trials. Medicine (Baltimore). 2020;99:e21969.

169. Owen PJ, Miller CT, Mundell NL, Verswijveren SJJM, Tagliaferri SD, Brisby H, et al. Which specific modes of exercise training are most effective for treating low back pain? Network meta-analysis. Br J Sports Med. 2020;54:1279–87.

170. Parreira P, Heymans MW, van Tulder MW, Esmail R, Koes BW, Poquet N, et al. Back Schools for chronic non-specific low back pain. Cochrane Database Syst Rev. 2017;8:CD011674.

171. Pereira LM, Obara K, Dias JM, Menacho MO, Guariglia DA, Schiavoni D, et al. Comparing the Pilates method with no exercise or lumbar stabilization for pain and functionality in patients with chronic low back pain: systematic review and meta-analysis. Clin Rehabil. 2012;26:10–20.

172. Pourahmadi M, Sahebalam M, Bagheri R. Effectiveness of Proprioceptive Neuromuscular Facilitation on Pain Intensity and Functional Disability in Patients with Low Back Pain: A Systematic Review and Meta-Analysis. Arch Bone Jt Surg. 2020;8:479–501.

173. Quentin C, Bagheri R, Ugbolue UC, Coudeyre E, Pélissier C, Descatha A, et al. Effect of Home Exercise Training in Patients with Nonspecific Low-Back Pain: A Systematic Review and Meta-Analysis. Int J Environ Res Public Health. 2021;18.

174. Searle A, Spink M, Ho A, Chuter V. Exercise interventions for the treatment of chronic low back pain: a systematic review and meta-analysis of randomised controlled trials. Clin Rehabil. 2015;29:1155–67.

175. Shi Z, Zhou H, Lu L, Pan B, Wei Z, Yao X, et al. Aquatic Exercises in the Treatment of Low Back Pain: A Systematic Review of the Literature and Meta-Analysis of Eight Studies. Am J Phys Med Rehabil. 2018;97:116–22.

176. Sitthipornvorakul E, Klinsophon T, Sihawong R, Janwantanakul P. The effects of walking intervention in patients with chronic low back pain: A meta-analysis of randomized controlled trials. Musculoskelet Sci Pract. 2018;34:38–46.

177. Slade SC, Keating JL. Unloaded movement facilitation exercise compared to no exercise or alternative therapy on outcomes for people with nonspecific chronic low back pain: a systematic review. J Manipulative Physiol Ther. 2007;30:301–11.

178. Smith BE, Littlewood C, May S. An update of stabilisation exercises for low back pain: a systematic review with meta-analysis. BMC Musculoskelet Disord. 2014;15:416.

179. Sun W, Zhang H, Lv C, Tang L, Tian S. Comparative efficacy of 12 non-drug interventions on non-specific chronic low back pain in nurses: A systematic review and network meta-analysis. J Back Musculoskelet Rehabil. 2021;34:499–510.

180. Tataryn N, Simas V, Catterall T, Furness J, Keogh JWL. Posterior-Chain Resistance Training Compared to General Exercise and Walking Programmes for the Treatment of Chronic Low Back Pain in the General Population: A Systematic Review and Meta-Analysis. Sports Med Open. 2021;7:17.

181. Thornton JS, Caneiro JP, Hartvigsen J, Ardern CL, Vinther A, Wilkie K, et al. Treating low back pain in athletes: a systematic review with meta-analysis. Br J Sports Med. 2021;55:656–62.

182. van Middelkoop M, Rubinstein SM, Kuijpers T, Verhagen AP, Ostelo R, Koes BW, et al. A systematic review on the effectiveness of physical and rehabilitation interventions for chronic non-specific low back pain. Eur Spine J. 2011;20:19–39.

183. Wood L, Foster NE, Lewis M, Bishop A. Exercise Interventions for Persistent Non-Specific Low Back Pain - Does Matching Outcomes to Treatment Targets Make a Difference? A Systematic Review and Meta-Analysis. J Pain. 2021;22:107–26.

184. Yamato T, Maher C, Saragiotto B, Hancock M, Ostelo R, Cabral C, et al. Pilates for low back pain. Cochrane Database of Systematic Reviews [Internet]. 2015; Available from: http://dx.doi.org/10.1002/14651858.CD010265.pub2

185. Yue YS, Wang XD, Xie B, Li ZH, Chen BL, Wang XQ, et al. Sling exercise for chronic low back pain: a systematic review and meta-analysis. PLoS One. 2014;9:e99307.

186. Zhu F, Zhang M, Wang D, Hong Q, Zeng C, Chen W. Yoga compared to non-exercise or physical therapy exercise on pain, disability, and quality of life for patients with chronic low back pain: A systematic review and meta-analysis of randomized controlled trials. PLoS One. 2020;15:e0238544.

187. Zou L, Zhang Y, Yang L, Loprinzi PD, Yeung AS, Kong J, et al. Are Mindful Exercises Safe and Beneficial for Treating Chronic Lower Back Pain? A Systematic Review and Meta-Analysis of Randomized Controlled Trials. J Clin Med. 2019;8.

188. Li H, Ge D, Liu S, Zhang W, Wang J, Si J, et al. Baduanjin exercise for low back pain: A systematic review and meta-analysis. Complement Ther Med. 2019;43:109–16.

189. Tomazoni SS, Almeida MO, Bjordal JM, Stausholm MB, Machado CDSM, Leal-Junior ECP, et al. Photobiomodulation therapy does not decrease pain and disability in people with non-specific low back pain: a systematic review. J Physiother. 2020;66:155–65.

190. Zhang Y, Loprinzi PD, Yang L, Liu J, Liu S, Zou L. The Beneficial Effects of Traditional Chinese Exercises for Adults with Low Back Pain: A Meta-Analysis of Randomized Controlled Trials. Medicina (Kaunas). 2019;55.

191. Saragiotto BT, Maher CG, Yamato TP, Costa LO, Menezes Costa LC, Ostelo RW, et al. Motor control exercise for chronic non-specific low-back pain. Cochrane Database Syst Rev. 2016;CD012004.

192. Hayden JA, van Tulder MW, Malmivaara A, Koes BW. Exercise therapy for treatment of non-specific low back pain. Cochrane Database Syst Rev. 2005;CD000335.

193. Gianola S, Bargeri S, Del Castillo G, Corbetta D, Turolla A, Andreano A, et al. Effectiveness of treatments for acute and subacute mechanical non-specific low back pain: a systematic review with network meta-analysis. Br J Sports Med. 2021;

194. Wewege MA, Booth J, Parmenter BJ. Aerobic vs. resistance exercise for chronic non-specific low back pain: A systematic review and meta-analysis. J Back Musculoskelet Rehabil. 2018;31:889–99.

195. Domingues de Freitas C, Costa DA, Junior NC, Civile VT. Effects of the pilates method on kinesiophobia associated with chronic non-specific low back pain: Systematic review and meta-analysis. J Bodyw Mov Ther. 2020;24:300–6.

196. Niederer D, Mueller J. Sustainability effects of motor control stabilisation exercises on pain and function in chronic nonspecific low back pain patients: A systematic review with meta-analysis and meta-regression. PLoS One. 2020;15:e0227423.

197. Vanti C, Andreatta S, Borghi S, Guccione AA, Pillastrini P, Bertozzi L. The effectiveness of walking versus exercise on pain and function in chronic low back pain: a systematic review and meta-analysis of randomized trials. Disabil Rehabil. 2019;41:622–32.

198. de Jesus FLA, Fukuda TY, Souza C, Guimarães J, Aquino L, Carvalho G, et al. Addition of specific hip strengthening exercises to conventional rehabilitation therapy for low back pain: a systematic review and meta-analysis. Clin Rehabil. 2020;34:1368–77.

199. Fernandez M, Hartvigsen J, Ferreira ML, Refshauge KM, Machado AF, Lemes ÍR, et al. Advice to Stay Active or Structured Exercise in the Management of Sciatica: A Systematic Review and Meta-analysis. Spine (Phila Pa 1976). 2015;40:1457–66.

200. Wang XQ, Zheng JJ, Yu ZW, Bi X, Lou SJ, Liu J, et al. A meta-analysis of core stability exercise versus general exercise for chronic low back pain. PLoS One. 2012;7:e52082.

201. Aladro-Gonzalvo AR, Araya-Vargas GA, Machado-Díaz M, Salazar-Rojas W. Pilates-based exercise for persistent, non-specific low back pain and associated functional disability: a meta-analysis with meta-regression. J Bodyw Mov Ther. 2013;17:125–36.

202. Anheyer D, Haller H, Lauche R, Dobos G, Cramer H. Yoga for treating low back pain: a systematic review and meta-analysis. Pain. 2021;

203. Byström MG, Rasmussen-Barr E, Grooten WJ. Motor control exercises reduces pain and disability in chronic and recurrent low back pain: a meta-analysis. Spine (Phila Pa 1976). 2013;38:E350-8.

204. Drummond C, Lebedeva V, Kirker K, Masaracchio M. Sling Exercise in the Management of Chronic Low Back Pain: A Systematic Review and Meta-Analysis. J Strength Cond Res. 2021;

205. Ebadi S, Henschke N, Forogh B, Nakhostin Ansari N, van Tulder MW, Babaei-Ghazani A, et al. Therapeutic ultrasound for chronic low back pain. Cochrane Database Syst Rev. 2020;7:CD009169.

206. Ford JJ, Bower SE, Ford I, de Mello MM, Carneiro SR, Balasundaram AP, et al. Effects of specific muscle activation for low back pain on activity limitation, pain, work participation, or recurrence: A systematic review. Musculoskelet Sci Pract. 2020;50:102276.

207. Gomes-Neto M, Lopes JM, Conceição CS, Araujo A, Brasileiro A, Sousa C, et al. Stabilization exercise compared to general exercises or manual therapy for the management of low back pain: A systematic review and meta-analysis. Phys Ther Sport. 2017;23:136–42.

208. Ram AK, Summers SJ, Booth J, Gibbs MT, Jones MD. Higher intensity exercise reduces disability more than lower intensity exercise in adults with chronic low back pain: A systematic review and meta-analysis. Musculoskeletal Care. 2023;

209. Ling-Xin L., Ke-Yao H., Rui Z., Zuo-Yan L., Li-Hui P. Efficacy and safety of proprioceptive neuromuscular facilitation for chronic low back pain: A meta-analysis of randomized controlled trials. Turkish Journal of Physical Medicine and Rehabilitation. 2022;68:439–46.

210. Gao P, Tang F, Liu W, Mo Y. The effects of proprioceptive neuromuscular facilitation in treating chronic low back pain: A systematic review and meta-analysis. J Back Musculoskelet Rehabil. 2022;35:21–33.

211. Shanbehzadeh S, ShahAli S, Hides J, Ebrahimi-Takamjani I, Rasouli O. Effect of Motor Control Training on Trunk Muscle Morphometry, Pain, and Disability in People With Chronic Low Back Pain: A Systematic Review and Meta-Analysis. J Manipulative Physiol Ther. 2022;45:202–15.

212. Zhang F, Zhao J, Jiang N, Zhai Q, Hu J, Zhang J. Meta-Analysis of Tai Chi Chuan in Treating Lumbar Spondylosis and Back Pain. Appl Bionics Biomech. 2022;2022:2759977.

213. Temporiti F, Ferrari S, Kieser M, Gatti R. Efficacy and characteristics of physiotherapy interventions in patients with lumbar spinal stenosis: a systematic review. Eur Spine J. 2022;31:1370–90.

214. Arcanjo FL, Martins JVP, Moté P, Leporace G, Oliveira DA, Sousa CS, et al. Proprioceptive neuromuscular facilitation training reduces pain and disability in individuals with chronic low back pain: A systematic review and meta-analysis. Complement Ther Clin Pract. 2022;46:101505.

215. Pocovi NC, de Campos TF, Christine Lin CW, Merom D, Tiedemann A, Hancock MJ. Walking, Cycling, and Swimming for Nonspecific Low Back Pain: A Systematic Review With Meta-analysis. J Orthop Sports Phys Ther. 2022;52:85–99.

216. Prat-Luri A, de Los Rios-Calonge J, Moreno-Navarro P, Manresa-Rocamora A, Vera-Garcia FJ, Barbado D. Effect of Trunk-Focused Exercises on Pain, Disability, Quality of Life, and Trunk Physical Fitness in Low Back Pain and How Potential Effect Modifiers Modulate Their Effects: A Systematic Review With Meta-analyses. J Orthop Sports Phys Ther. 2023;53:64–93.

217. Pourahmadi M, Delavari S, Hayden JA, Keshtkar A, Ahmadi M, Aletaha A, et al. Does motor control training improve pain and function in adults with symptomatic lumbar disc herniation? A systematic review and meta-analysis of 861 subjects in 16 trials. Br J Sports Med. 2022;

218. Ranjan R, Singh S, Sandhya K, Chauhan G, Kumar A. Comparison of Proprioceptive Neuromuscular Facilitation with other exercises on Pain and Disability in patients with Non-specific Chronic Low Back Pain: A Meta Analysis. Indian Journal of Physiotherapy & Occupational Therapy. 2023;17:80–7.

219. Sutanto D, Ho RST, Poon ETC, Yang Y, Wong SHS. Effects of Different Trunk Training Methods for Chronic Low Back Pain: A Meta-Analysis. Int J Environ Res Public Health. 2022;19.

220. Wieland L, Skoetz N, Pilkington K, Harbin S, Vempati R, Berman B. Yoga for chronic non‐specific low back pain. Cochrane Database of Systematic Reviews [Internet]. 2022; Available from: http://dx.doi.org/10.1002/14651858.CD010671.pub3

221. Shi J., Hu Z.-Y., Wen Y.-R., Wang Y.-F., Lin Y.-Y., Zhao H.-Z., et al. Optimal modes of mind-body exercise for treating chronic non-specific low back pain: Systematic review and network meta-analysis. Frontiers in Neuroscience. 2022;16:1046518.

222. Zhang C, Li Y, Zhong Y, Feng C, Zhang Z, Wang C. Effectiveness of motor control exercise on non-specific chronic low back pain, disability and core muscle morphological characteristics: a meta-analysis of randomized controlled trials. Eur J Phys Rehabil Med. 2021;57:793–806.

223. Fernández-Rodríguez R, Álvarez-Bueno C, Cavero-Redondo I, Torres-Costoso A, Pozuelo-Carrascosa DP, Reina-Gutiérrez S, et al. Best Exercise Options for Reducing Pain and Disability in Adults With Chronic Low Back Pain: Pilates, Strength, Core-Based, and Mind-Body. A Network Meta-analysis. J Orthop Sports Phys Ther. 2022;52:505–21.

224. Gilliam JR, George SZ, Norman KS, Hendren S, Sahu PK, Silfies SP. Mind-Body Exercise Performed by Physical Therapists for Reducing Pain and Disability in Low Back Pain: A Systematic Review With Meta-analysis. Arch Phys Med Rehabil. 2022;

225. Fleckenstein J, Floessel P, Engel T, Krempel L, Stoll J, Behrens M, et al. Individualized Exercise in Chronic Non-Specific Low Back Pain: A Systematic Review with Meta-Analysis on the Effects of Exercise Alone or in Combination with Psychological Interventions on Pain and Disability. J Pain. 2022;23:1856–73.

226. Zhang SK, Yang Y, Gu ML, Mao SJ, Zhou WS. Effects of Low Back Pain Exercises on Pain Symptoms and Activities of Daily Living: A Systematic Review and Meta-Analysis. Percept Mot Skills. 2022;129:63–89.

227. de Zoete RMJ, Brown L, Oliveira K, Penglaze L, Rex R, Sawtell B, et al. The effectiveness of general physical exercise for individuals with chronic neck pain: a systematic review of randomised controlled trials. European Journal of Physiotherapy. 2020;22:141–7.

228. Cox LG, Kidgell DJ, Iles RA. Neck-specific strengthening exercises and cognitive therapy for chronic neck pain: a systematic review. Physical Therapy Reviews. 2019;24:335–45.

229. Gross A, Kay TM, Paquin J-P, Blanchette S, Lalonde P, Christie T, et al. Exercises for mechanical neck disorders. Cochrane Database Syst Rev. 2015;1:CD004250.

230. Leaver AM, Refshauge KM, Maher CG, McAuley JH. Conservative interventions provide short-term relief for non-specific neck pain: a systematic review. J Physiother. 2010;56:73–85.

231. Li Y, Li S, Jiang J, Yuan S. Effects of yoga on patients with chronic nonspecific neck pain: A PRISMA systematic review and meta-analysis. Medicine (Baltimore). 2019;98:e14649.

232. Lin KY, Tsai YJ, Hsu PY, Tsai CS, Kuo YL. Effects of Sling Exercise for Neck Pain: A Systematic Review and Meta-Analysis. Phys Ther. 2021;101.

233. Louw S, Makwela S, Manas L, Meyer L, Terblanche D, Brink Y. Effectiveness of exercise in office workers with neck pain: A systematic review and meta-analysis. S Afr J Physiother. 2017;73:392.

234. Martin-Gomez C, Sestelo-Diaz R, Carrillo-Sanjuan V, Navarro-Santana MJ, Bardon-Romero J, Plaza-Manzano G. Motor control using cranio-cervical flexion exercises versus other treatments for non-specific chronic neck pain: A systematic review and meta-analysis. Musculoskelet Sci Pract. 2019;42:52–9.

235. Nunes AMP, Moita JPAM. Effectiveness of physical and rehabilitation techniques in reducing pain in chronic trapezius myalgia: A systematic review and meta-analysis. International Journal of Osteopathic Medicine. 2015;18:189–206.

236. Salt E, Wright C, Kelly S, Dean A. A systematic literature review on the effectiveness of non-invasive therapy for cervicobrachial pain. Man Ther. 2011;16:53–65.

237. Tsiringakis G, Dimitriadis Z, Triantafylloy E, McLean S. Motor control training of deep neck flexors with pressure biofeedback improves pain and disability in patients with neck pain: A systematic review and meta-analysis. Musculoskelet Sci Pract. 2020;50:102220.

238. Wu B, Yuan H, Geng D, Zhang L, Zhang C. The Impact of a Stabilization Exercise on Neck Pain: A Systematic Review and Meta-analysis. J Neurol Surg A Cent Eur Neurosurg. 2020;81:342–7.

239. Chrcanovic B, Larsson J, Malmström EM, Westergren H, Häggman-Henrikson B. Exercise therapy for whiplash-associated disorders: a systematic review and meta-analysis. Scand J Pain. 2021;

240. Wilhelm MP, Donaldson M, Griswold D, Learman KE, Garcia AN, Learman SM, et al. The Effects of Exercise Dosage on Neck-Related Pain and Disability: A Systematic Review With Meta-analysis. J Orthop Sports Phys Ther. 2020;50:607–21.

241. Griffin A, Leaver A, Moloney N. General Exercise Does Not Improve Long-Term Pain and Disability in Individuals With Whiplash-Associated Disorders: A Systematic Review. J Orthop Sports Phys Ther. 2017;47:472–80.

242. Fredin K, Lorås H. Manual therapy, exercise therapy or combined treatment in the management of adult neck pain - A systematic review and meta-analysis. Musculoskelet Sci Pract. 2017;31:62–71.

243. Bertozzi L, Gardenghi I, Turoni F, Villafañe JH, Capra F, Guccione AA, et al. Effect of therapeutic exercise on pain and disability in the management of chronic nonspecific neck pain: systematic review and meta-analysis of randomized trials. Phys Ther. 2013;93:1026–36.

244. Garzonio S, Arbasetti C, Geri T, Testa M, Carta G. Effectiveness of Specific Exercise for Deep Cervical Muscles in Nonspecific Neck Pain: A Systematic Review and Meta-Analysis. Phys Ther. 2022;102.

245. Villanueva-Ruiz I, Falla D, Lascurain-Aguirrebeña I. Effectiveness of Specific Neck Exercise for Nonspecific Neck Pain; Usefulness of Strategies for Patient Selection and Tailored Exercise-A Systematic Review With Meta-Analysis. Phys Ther. 2022;102.

246. Castellini G, Pillastrini P, Vanti C, Bargeri S, Giagio S, Bordignon E, et al. Some conservative interventions are more effective than others for people with chronic non-specific neck pain: a systematic review and network meta-analysis. J Physiother. 2022;68:244–54.

247. Xie YH, Liao MX, Wang MY, Fernando WCHA, Gu YM, Wang XQ, et al. Traditional Chinese Mind and Body Exercises for Neck Pain: A Meta-Analysis of Randomized Controlled Trials. Pain Res Manag. 2021;2021:5426595.

248. Kong L., Ren J., Fang S., He T., Zhou X., Fang M. Traditional Chinese Exercises on Pain and Disability in Middle-Aged and Elderly Patients With Neck Pain: A Systematic Review and Meta-Analysis of Randomized Controlled Trials. Frontiers in Aging Neuroscience. 2022;14:912945.

249. Yang J, Yang M, Lin Q, Fu J, Xi R. Effects of isometric training on the treatment of patients with neck pain: A meta-analysis. Medicine (Baltimore). 2022;101:e30864.

250. Baillet A, Zeboulon N, Gossec L, Combescure C, Bodin LA, Juvin R, et al. Efficacy of cardiorespiratory aerobic exercise in rheumatoid arthritis: meta-analysis of randomized controlled trials. Arthritis Care Res (Hoboken). 2010;62:984–92.

251. Baillet A, Vaillant M, Guinot M, Juvin R, Gaudin P. Efficacy of resistance exercises in rheumatoid arthritis: meta-analysis of randomized controlled trials. Rheumatology (Oxford). 2012;51:519–27.

252. Hurkmans E, van der Giesen FJ, Vliet Vlieland TP, Schoones J, Van den Ende EC. Dynamic exercise programs (aerobic capacity and/or muscle strength training) in patients with rheumatoid arthritis. Cochrane Database Syst Rev. 2009;2009:CD006853.

253. Mudano A, Tugwell P, Wells G, Singh J. Tai Chi for rheumatoid arthritis. Cochrane Database of Systematic Reviews [Internet]. 2019; Available from: http://dx.doi.org/10.1002/14651858.CD004849.pub2

254. Williams M, Srikesavan C, Heine P, Bruce J, Brosseau L, Hoxey‐Thomas N, et al. Exercise for rheumatoid arthritis of the hand. Cochrane Database of Systematic Reviews [Internet]. 2018; Available from: http://dx.doi.org/10.1002/14651858.CD003832.pub3

255. Sobue Y, Kojima T, Ito H, Nishida K, Matsushita I, Kaneko Y, et al. Does exercise therapy improve patient-reported outcomes in rheumatoid arthritis? A systematic review and meta-analysis for the update of the 2020 JCR guidelines for the management of rheumatoid arthritis. Mod Rheumatol. 2022;32:96–104.

256. Wu H., Wang Q., Wen G., Wu J., Wang Y. The effects of Tai Chi on physical function and safety in patients with rheumatoid arthritis: A systematic review and meta-analysis. Frontiers in Physiology. 2023;14:1079841.

257. Ye H., Weng H., Xu Y., Wang L., Wang Q., Xu G. Effectiveness and safety of aerobic exercise for rheumatoid arthritis: a systematic review and meta-analysis of randomized controlled trials. BMC Sports Science, Medicine and Rehabilitation. 2022;14:17.

258. Gutiérrez-Espinoza H, Araya-Quintanilla F, Cereceda-Muriel C, Álvarez-Bueno C, Martínez-Vizcaíno V, Cavero-Redondo I. Effect of supervised physiotherapy versus home exercise program in patients with subacromial impingement syndrome: A systematic review and meta-analysis. Phys Ther Sport. 2020;41:34–42.

259. Desmeules F, Boudreault J, Roy JS, Dionne C, Frémont P, MacDermid JC. The efficacy of therapeutic ultrasound for rotator cuff tendinopathy: A systematic review and meta-analysis. Phys Ther Sport. 2015;16:276–84.

260. Dong W, Goost H, Lin XB, Burger C, Paul C, Wang ZL, et al. Treatments for shoulder impingement syndrome: a PRISMA systematic review and network meta-analysis. Medicine (Baltimore). 2015;94:e510.

261. Naunton J, Street G, Littlewood C, Haines T, Malliaras P. Effectiveness of progressive and resisted and non-progressive or non-resisted exercise in rotator cuff related shoulder pain: a systematic review and meta-analysis of randomized controlled trials. Clin Rehabil. 2020;34:1198–216.

262. Hanratty CE, McVeigh JG, Kerr DP, Basford JR, Finch MB, Pendleton A, et al. The effectiveness of physiotherapy exercises in subacromial impingement syndrome: a systematic review and meta-analysis. Semin Arthritis Rheum. 2012;42:297–316.

263. Mertens MG, Meert L, Struyf F, Schwank A, Meeus M. Exercise therapy is effective for improvement in range of motion, function and pain in patients with frozen shoulder: a systematic review and meta-analysis. Arch Phys Med Rehabil. 2021;

264. Larsson R, Bernhardsson S, Nordeman L. Effects of eccentric exercise in patients with subacromial impingement syndrome: a systematic review and meta-analysis. BMC Musculoskelet Disord. 2019;20:446.

265. Liaghat B, Ussing A, Petersen BH, Andersen HK, Barfod KW, Jensen MB, et al. Supervised Training Compared With No Training or Self-training in Patients With Subacromial Pain Syndrome: A Systematic Review and Meta-analysis. Arch Phys Med Rehabil. 2021;

266. Brudvig TJ, Kulkarni H, Shah S. The effect of therapeutic exercise and mobilization on patients with shoulder dysfunction : a systematic review with meta-analysis. J Orthop Sports Phys Ther. 2011;41:734–48.

267. Marinko LN, Chacko JM, Dalton D, Chacko CC. The effectiveness of therapeutic exercise for painful shoulder conditions: a meta-analysis. J Shoulder Elbow Surg. 2011;20:1351–9.

268. Shire AR, Stæhr TAB, Overby JB, Bastholm Dahl M, Sandell Jacobsen J, Høyrup Christiansen D. Specific or general exercise strategy for subacromial impingement syndrome-does it matter? A systematic literature review and meta analysis. BMC Musculoskelet Disord. 2017;18:158.

269. Sharma S, Hussain ME, Sharma S. Manual therapy combined with therapeutic exercise vs therapeutic exercise alone for shoulder impingement syndrome: A systematic review and meta-analysis. Journal of Clinical and Diagnostic Research. 2021;15:YE10–7.

270. Picón SPB, Batista GA, Pitangui ACR, de Araújo RC. Effects of Workplace-Based Intervention for Shoulder Pain: A Systematic Review and Meta-analysis. J Occup Rehabil. 2021;31:243–62.

271. Steuri R, Sattelmayer M, Elsig S, Kolly C, Tal A, Taeymans J, et al. Effectiveness of conservative interventions including exercise, manual therapy and medical management in adults with shoulder impingement: a systematic review and meta-analysis of RCTs. Br J Sports Med. 2017;51:1340–7.

272. Babatunde OO, Ensor J, Littlewood C, Chesterton L, Jordan JL, Corp N, et al. Comparative effectiveness of treatment options for subacromial shoulder conditions: a systematic review and network meta-analysis. Ther Adv Musculoskelet Dis. 2021;13:1759720X211037530.

273. Celik D, Karaborklu Argut S, Coban O, Eren I. The clinical efficacy of kinesio taping in shoulder disorders: a systematic review and meta analysis. Clin Rehabil. 2020;34:723–40.

274. Liu J, Sai-Chuen Hui S, Yang Y, Rong X, Zhang R. Effectiveness of Home-Based Exercise for Nonspecific Shoulder Pain: A Systematic Review and Meta-analysis. Arch Phys Med Rehabil. 2022;103:2036–50.

# Supplementary file 7. AMSTAR-2 ratings for included reviews

| Review | Item 1 | Item 2 | Item 3 | Item 4 | Item 5 | Item 6 | Item 7 | Item 8 | Item 9 | Item 10 | Item 11 | Item 12 | Item 13 | Item 14 | Item 15 | Item 16 | Overall rating |
| --- | --- | --- | --- | --- | --- | --- | --- | --- | --- | --- | --- | --- | --- | --- | --- | --- | --- |
| Aladro-Gonzalvo 2013 | + | - | - | / | + | + | + | / | + | - | + | + | + | + | + | + | Low |
| Alammari 2023 | + | + | - | - | + | - | - | + | + | - | - | - | - | - | - | + | Critically low |
| Albuquerque 2022 | + | / | - | - | + | - | - | / | + | - | + | - | - | + | - | + | Critically low |
| AL-Mhanna 2022 | - | / | - | / | + | + | - | + | + | - | + | - | + | + | - | + | Critically low |
| Amaral 2020 | + | + | - | / | + | + | - | + | + | - | - | + | + | + | + | + | Critically low |
| Amiri 2022 | + | - | - | - | - | - | - | / | + | - | - | - | - | - | + | + | Critically low |
| Anheyer 2021 | + | + | - | / | + | + | + | + | + | + | + | + | + | + | - | + | Low |
| Anwer 2016 | + | - | - | - | + | + | - | / | + | - | - | - | - | + | - | + | Critically low |
| Anwer 2018 | + | / | - | - | + | + | / | / | + | - | - | - | - | - | - | + | Critically low |
| Arcanjo 2022 | - | - | - | / | + | + | - | / | + | - | + | - | - | - | - | + | Critically low |
| Arora 2022 | + | / | - | - | + | + | - | / | + | - | - | - | - | - | - | + | Critically low |
| Babatunde 2021 | + | + | - | + | + | + | / | / | + | - | + | + | + | + | - | + | Low |
| Baillet 2010 | + | - | + | - | - | - | - | / | / | - | + | + | + | + | + | + | Critically low |
| Baillet 2012 | + | - | - | - | - | + | - | / | / | - | + | + | + | + | + | + | Critically low |
| Barker 2014 | + | - | - | - | + | + | + | / | + | - | + | + | + | + | - | - | Critically low |
| Bartels 2016 | + | + | - | + | + | + | + | + | + | + | + | - | - | + | - | + | Critically low |
| Bartholdy 2017 | + | + | - | / | + | + | - | / | + | - | + | - | + | + | - | + | Critically low |
| Batterham 2011 | + | - | - | / | + | + | - | / | + | - | + | - | - | + | - | + | Critically low |
| Bertozzi 2013 | + | - | - | / | + | + | - | + | + | - | + | - | - | + | + | - | Critically low |
| Bertozzi 2015 | + | - | - | / | + | + | - | + | + | - | - | - | - | - | + | + | Critically low |
| Beumer 2016 | + | - | - | / | + | + | / | + | + | - | + | - | - | + | - | + | Critically low |
| Bidonde 2014 | + | / | - | / | + | + | + | + | + | + | + | - | - | + | - | + | Critically low |
| Bidonde 2017 | + | + | - | / | + | + | + | + | + | + | + | + | + | + | + | + | High |
| Bidonde 2019 | + | + | - | + | + | + | + | + | + | + | + | + | + | + | + | + | High |
| Thorlund 2022 | + | / | - | / | + | + | - | - | + | - | + | - | - | + | + | + | Critically low |
| Bravo 2019 | + | - | - | - | + | - | - | + | + | - | + | - | + | + | - | + | Critically low |
| Brosseau 2004 | + | - | - | - | + | + | + | + | / | - | - | - | - | - | - | - | Critically low |
| Brudvig 2011 | + | - | - | - | + | + | - | / | - | - | + | - | - | + | - | - | Critically low |
| Busch 2007 | + | / | - | / | + | + | + | + | + | - | + | + | + | + | + | + | Moderate |
| Busch 2013 | + | + | - | / | + | + | + | + | + | + | + | + | + | + | - | + | Low |
| Byström 2013 | + | - | - | / | + | - | - | + | + | - | - | - | - | + | - | + | Critically low |
| Cardoso 2017 | + | - | - | - | - | + | - | - | - | - | - | - | - | - | - | - | Critically low |
| Castellini 2022 | + | + | - | / | + | + | - | + | + | - | + | + | + | + | + | + | Low |
| Celik 2020 | + | - | - | - | + | + | - | / | + | - | - | - | - | - | - | + | Critically low |
| Challoumas 2021 | + | - | - | - | - | - | - | / | + | - | + | - | - | + | - | + | Critically low |
| Chen 2019 | + | - | - | - | - | + | - | + | + | - | + | - | - | - | - | + | Critically low |
| Chen 2021 | + | / | - | / | - | + | - | + | + | - | + | - | - | + | + | + | Critically low |
| Cheng 2019 | + | / | - | / | - | + | - | / | + | - | + | - | - | + | - | + | Critically low |
| Chrcanovic 2021 | + | + | - | / | + | + | + | + | + | - | - | - | - | + | - | + | Critically low |
| Clijsen 2014 | + | / | - | / | - | - | - | + | + | - | + | + | + | + | + | - | Low |
| Collado-Mateo 2018 | - | - | - | - | + | + | - | / | + | - | - | - | - | - | - | + | Critically low |
| Corbett 2013 | + | - | - | / | + | + | - | / | + | - | + | + | + | + | + | + | Critically low |
| Coudeyre 2016 | - | / | - | / | + | - | - | / | / | - | + | - | + | - | + | + | Low |
| Couto 2022 | + | + | - | - | + | - | - | / | + | - | + | - | - | + | - | + | Critically low |
| Cox 2019 | + | - | - | - | + | - | - | / | + | - | - | - | - | - | - | + | Critically low |
| Cuenca-Martinez 2022 | + | / | - | / | + | - | - | / | + | - | + | - | - | + | + | + | Critically low |
| Cuyul-Vásquez 2020 | + | + | - | - | + | + | / | / | + | - | - | - | - | + | - | + | Critically low |
| Silva 2022 | + | / | - | / | + | + | + | / | + | - | + | + | + | + | + | + | Moderate |
| Denham-Jones 2021 (a) | + | - | + | / | + | + | - | + | + | - | - | - | - | - | - | + | Critically low |
| Denham‐Jones 2021 (b) | + | - | - | / | + | - | - | - | + | - | - | - | - | + | - | + | Critically low |
| Desmeules 2015 | + | - | + | - | + | - | + | + | + | - | - | - | - | - | - | + | Critically low |
| Devos-Comby 2006 | + | - | - | - | - | - | - | + | - | - | + | - | - | + | + | - | Critically low |
| deZoete 2020 | + | + | - | - | + | + | - | / | + | - | - | - | - | - | + | + | Critically low |
| Dong 2015 | + | - | - | / | + | + | - | / | + | - | + | + | + | + | - | + | Critically low |
| Dong 2018 | + | / | - | / | + | + | - | + | + | - | - | - | - | + | + | + | Critically low |
| Dong 2019 | + | - | - | - | + | + | - | + | + | - | + | - | - | + | + | + | Critically low |
| Drummond 2021 | + | - | - | / | + | + | - | / | + | - | - | - | - | - | + | - | Critically low |
| Duan 2022 | + | / | - | / | + | + | - | + | + | - | - | - | - | + | + | + | Critically low |
| Ebadi 2020 | + | + | - | + | + | + | + | + | + | + | + | + | + | + | - | + | Low |
| Escalante 2010 | + | - | - | / | - | - | - | - | / | - | - | - | - | - | - | - | Critically low |
| Fernandez 2015 | + | - | - | / | + | + | - | + | + | - | - | - | - | + | - | + | Critically low |
| Fernandez-Rodriguez 2022 | + | / | - | / | - | + | + | / | + | - | + | + | + | + | + | + | Moderate |
| Fernandopulle 2017 | + | - | - | / | + | + | - | + | + | - | - | - | - | - | - | + | Critically low |
| Ferreira 2006 | + | - | - | + | + | - | - | - | + | - | - | - | - | - | - | + | Critically low |
| Fleckenstein 2022 | + | / | - | - | + | + | / | / | + | - | + | + | + | + | + | + | Low |
| Ford 2020 | + | + | - | / | + | + | - | / | + | - | - | - | - | - | + | + | Critically low |
| Franco 2021 | + | + | + | / | + | + | - | + | + | - | - | - | - | - | - | + | Critically low |
| Fransen 2002 | + | - | - | / | + | + | + | / | + | - | - | - | - | - | - | - | Critically low |
| Fransen 2010 | + | - | - | / | + | + | + | - | + | - | - | - | - | - | - | + | Critically low |
| Fransen 2014 | + | + | - | / | + | + | + | / | + | - | + | + | + | + | - | + | Low |
| Fransen 2015 | + | + | - | - | + | + | + | + | + | - | + | + | + | + | - | + | Critically low |
| Fredin 2017 | + | - | - | / | + | + | - | + | + | - | - | + | + | - | - | + | Critically low |
| Freitas 2020 | + | + | - | / | + | + | - | - | + | - | - | + | + | + | - | + | Critically low |
| French 2022 | + | + | - | / | + | + | + | + | + | + | + | + | + | + | + | + | High |
| Galvao-Moreira 2021 | + | + | - | - | + | + | + | / | + | - | + | - | - | + | + | + | Critically low |
| Gao 2022 | + | / | - | / | + | - | - | / | + | - | + | - | - | + | + | + | Critically low |
| Garzonio 2022 | + | + | - | - | + | + | + | / | + | - | + | - | - | + | - | + | Critically low |
| Gianola 2021 | + | + | - | / | + | + | - | / | + | - | + | - | - | + | + | + | Critically low |
| Gilliam 2022 | + | - | - | / | + | + | - | / | + | - | + | - | - | + | - | + | Critically low |
| Goff 2021 | + | / | - | / | + | + | + | / | + | - | + | - | - | + | - | + | Critically low |
| Goh 2019 | + | + | - | - | - | + | - | / | + | - | - | - | - | + | + | + | Critically low |
| Gomes-Neto 2017 | + | - | - | / | + | + | - | / | + | - | - | - | - | - | - | + | Critically low |
| Grantham 2021 | + | / | - | + | - | - | - | / | + | - | + | + | + | + | + | + | Low |
| Griffin 2017 | + | / | - | / | + | - | - | + | + | - | - | - | - | + | - | + | Critically low |
| Gross 2015 | + | / | - | / | + | + | + | + | + | - | + | + | + | + | + | + | Moderate |
| Guo 2022 (a) | + | / | - | / | + | + | + | / | + | - | + | - | - | + | + | + | Low |
| Guo 2022 (b) | + | / | - | / | + | + | - | / | + | - | + | - | - | + | - | + | Critically low |
| Gutierrez-Espinoza 2020 | + | / | - | - | + | + | - | + | + | - | + | - | - | - | - | + | Critically low |
| Guzman-Pavon 2020 | + | + | - | - | + | + | - | / | + | - | + | - | - | + | + | + | Critically low |
| Hall 2008 | + | - | - | - | + | - | - | + | / | - | + | - | - | - | - | + | Critically low |
| Hall 2009 | - | - | - | / | - | + | - | / | + | - | + | - | - | + | - | - | Critically low |
| Hanratty 2012 | - | + | - | - | - | - | - | + | + | - | + | - | - | + | - | + | Critically low |
| Hansen 2020 | + | / | - | / | + | + | / | + | + | - | - | - | - | - | - | + | Critically low |
| Hayden 2005 | + | / | - | / | + | + | + | + | + | - | + | + | + | + | + | + | Moderate |
| Hayden 2020 | + | + | - | / | - | - | - | / | + | - | + | - | - | + | - | + | Critically low |
| Hayden 2021 | + | + | + | / | + | + | + | + | + | + | + | + | + | + | + | + | High |
| Heijden 2015 | + | + | - | + | + | + | + | + | + | - | + | + | + | + | - | + | Low |
| Hernandez-Molina 2008 | + | - | - | / | + | + | - | / | - | - | + | - | - | + | - | - | Critically low |
| Hislop 2020 | + | / | - | - | - | - | - | / | + | - | + | - | - | + | - | + | Critically low |
| Holtzman 2013 | - | - | + | - | - | - | - | + | / | - | + | - | - | - | - | - | Critically low |
| Hu 2021 | + | - | - | / | + | + | - | + | + | - | + | - | + | - | + | + | Critically low |
| Hua 2022 | + | - | - | - | - | + | - | + | + | - | + | - | - | + | - | + | Critically low |
| Hurkmans 2009 | + | + | - | / | + | + | + | + | + | - | + | + | + | + | - | + | Low |
| Hurley 2018 | + | + | - | + | + | + | + | + | + | - | + | - | + | + | + | + | Moderate |
| Imoto 2019 | - | - | - | / | + | - | - | - | + | - | + | + | + | + | + | + | Critically low |
| Jacobi 2021 | + | + | - | / | + | + | - | + | + | - | - | - | - | - | - | - | Critically low |
| Jansen 2011 | + | - | - | - | - | + | - | + | + | - | + | + | + | + | - | + | Critically low |
| Jeong 2019 | + | - | - | / | + | + | - | + | + | - | - | - | - | - | - | - | Critically low |
| Jesus 2020 | + | - | - | / | + | + | / | / | + | - | - | - | - | + | - | + | Critically low |
| Juhl 2014 | + | - | - | / | + | + | - | / | + | - | + | + | + | + | - | - | Critically low |
| Jurado-Castro 2022 | + | / | - | - | - | - | - | / | + | - | + | - | - | + | - | + | Critically low |
| Karanasios 2021 | + | + | - | / | + | + | - | + | + | - | + | - | - | + | - | + | Critically low |
| Keller 2007 | + | - | - | / | - | - | - | / | / | - | - | - | - | - | - | - | Critically low |
| Kim 2019 | + | + | - | + | + | + | + | + | + | + | + | + | + | + | + | + | High |
| Kong 2016 | + | + | - | / | - | + | + | / | + | - | + | - | - | + | + | + | Low |
| Kong 2022 | + | + | - | / | + | + | - | / | + | - | + | - | - | + | + | + | Critically low |
| Kundakci 2022 | + | + | - | / | + | + | - | - | + | + | + | + | + | + | + | + | Low |
| Lack 2015 | + | - | - | - | + | + | - | + | + | - | - | - | - | - | - | + | Critically low |
| Lafrance 2021 | + | + | - | - | + | - | - | / | + | - | + | - | - | + | + | + | Critically low |
| Langhorst 2013 | + | - | - | / | + | + | - | + | + | - | + | + | + | + | + | - | Critically low |
| Larsson 2019 | + | / | + | / | + | + | - | + | + | - | + | - | - | + | - | + | Critically low |
| Lauche 2013 | + | - | - | / | + | + | + | + | + | - | + | + | + | + | - | + | Critically low |
| Lauche 2019 | + | - | - | / | + | + | - | / | + | - | + | + | + | + | + | + | Critically low |
| Leaver 2010 | + | - | - | / | + | + | - | / | + | - | - | - | - | - | - | + | Critically low |
| Li 2015 | + | / | - | + | + | + | - | + | + | - | - | - | - | - | - | + | Critically low |
| Li 2016 | + | - | - | / | + | + | - | / | + | - | + | - | - | + | - | + | Critically low |
| Li 2019 (a) | + | + | - | / | + | + | - | / | + | - | + | - | - | + | - | + | Critically low |
| Li 2019 (b) | + | / | - | / | + | + | - | / | + | - | + | - | - | + | - | + | Critically low |
| Li 2020 | + | - | - | - | + | + | - | / | + | - | + | - | - | + | + | + | Critically low |
| Li 2021 | - | + | - | - | + | + | - | / | + | - | + | - | - | + | + | + | Critically low |
| Liaghat 2021 | + | / | - | + | + | + | - | / | + | - | + | - | - | + | - | - | Critically low |
| Lim 2011 | + | - | - | - | + | + | / | + | + | - | + | - | - | + | + | - | Critically low |
| Lin 2021 | + | + | - | / | + | + | - | + | + | - | + | - | - | + | - | + | Critically low |
| Ling-Xin 2022 | + | - | - | - | + | + | - | - | + | - | + | - | - | + | + | + | Critically low |
| Liu 2022 | + | + | - | - | + | + | - | + | + | - | + | - | - | + | - | - | Critically low |
| Louw 2017 | + | - | - | - | + | + | - | / | + | - | - | - | - | - | - | + | Critically low |
| Lu 2015 | + | - | - | / | + | + | - | / | + | + | - | - | - | - | - | + | Critically low |
| Luan 2021 | + | - | - | / | + | + | - | / | + | - | - | - | - | + | - | + | Critically low |
| Luan 2022 | + | - | - | / | + | + | - | / | + | - | - | - | - | + | - | + | Critically low |
| Luomajoki 2018 | + | / | - | - | + | - | - | / | + | - | + | - | - | + | - | + | Critically low |
| Ma 2022 | + | / | - | - | + | + | - | / | + | - | + | - | - | + | - | + | Critically low |
| Macedo 2016 | + | / | + | / | + | + | + | + | + | + | + | - | - | - | - | + | Critically low |
| Maciel 2018 | + | / | - | - | - | + | - | / | + | + | - | - | - | + | - | - | Critically low |
| Magni 2017 | + | - | - | + | + | - | - | + | + | - | - | - | - | - | + | + | Critically low |
| Manojlovic 2021 | + | - | - | - | + | - | / | / | + | - | + | - | - | + | - | + | Critically low |
| Marinko 2011 | + | - | - | - | + | + | + | / | + | - | - | - | - | - | - | + | Critically low |
| Martin-Gomez 2019 | + | / | - | - | + | + | - | + | + | - | + | - | - | + | + | + | Critically low |
| MataDiz 2017 | + | / | - | / | + | + | - | + | + | - | + | - | - | + | - | + | Critically low |
| McDowell 2017 | + | - | - | - | - | - | + | / | / | - | + | - | - | + | + | + | Critically low |
| Meng 2015 | + | - | - | + | - | + | - | / | - | - | + | - | - | + | + | + | Critically low |
| Mertens 2021 | + | - | - | - | + | + | - | + | + | - | - | - | - | - | - | + | Critically low |
| Miller 2021 | + | + | - | / | + | + | - | / | + | - | + | - | - | + | + | + | Critically low |
| Miyamoto 2013 | + | - | - | / | + | - | - | / | + | - | + | + | + | + | + | - | Critically low |
| Moseng 2017 | + | / | - | - | + | + | + | + | + | - | + | - | - | + | - | + | Critically low |
| Mudano 2019 | + | + | - | / | + | + | + | + | + | + | + | + | + | + | - | + | Low |
| Mueller 2020 | + | - | - | - | + | + | - | / | + | - | + | + | + | + | + | + | Critically low |
| Murillo-Garcia 2022 | + | - | - | - | - | - | + | + | + | - | + | - | - | + | - | + | Critically low |
| Murphy 2019 | + | + | - | + | + | + | - | / | + | - | + | + | + | + | - | + | Critically low |
| Na 2021 | + | - | - | / | + | + | - | / | + | - | - | - | - | + | - | + | Critically low |
| Nascimento 2018 | + | / | - | / | + | + | + | / | + | - | - | - | - | + | - | + | Critically low |
| Naunton 2020 | + | + | - | - | + | + | - | + | + | + | + | + | + | - | - | + | Critically low |
| Nduwimana 2020 | + | + | - | - | + | + | - | - | + | - | + | + | + | + | - | + | Critically low |
| Neal 2022 | - | / | - | - | + | - | - | / | + | - | - | - | - | - | - | + | Critically low |
| Newberry 2017 | + | / | - | - | + | + | + | + | + | + | - | - | - | - | - | + | Critically low |
| Niederer 2020 | + | - | - | - | + | + | - | / | + | - | + | + | + | + | + | + | Critically low |
| Nunes 2015 | + | - | - | - | - | + | - | + | + | - | - | - | - | - | - | + | Critically low |
| O'Connor 2015 | + | / | - | / | + | + | + | / | / | + | + | + | + | + | + | + | High |
| Ortega-Castillo 2022 | + | / | - | - | - | - | - | / | + | - | - | - | - | + | - | + | Critically low |
| Osteras 2017 | + | + | - | + | + | + | + | + | + | + | + | - | - | + | - | + | Critically low |
| Ouellet 2021 | + | / | - | - | + | + | - | + | + | - | + | - | - | + | - | - | Critically low |
| Owen 2019 | + | + | - | / | + | + | - | / | + | - | + | - | - | - | + | + | Critically low |
| Parreira 2017 | + | + | - | + | + | + | + | + | + | + | + | + | + | + | - | + | Low |
| Pedersen 2022 | + | + | - | / | + | + | - | - | + | + | + | + | + | + | + | + | Low |
| Pereira 2011 | + | - | - | / | - | - | - | / | + | - | + | - | - | - | - | + | Critically low |
| Picón 2021 | + | / | - | / | + | - | - | + | + | - | + | - | - | + | - | + | Critically low |
| Pocovi 2022 | + | / | - | / | + | + | + | + | + | - | + | - | - | + | + | + | Low |
| Polaski 2019 | + | - | - | - | - | - | - | / | + | - | - | - | - | + | + | + | Critically low |
| Pourahmadi 2020 | + | + | - | - | + | + | - | / | + | - | + | - | - | + | + | + | Critically low |
| Pourahmadi 2022 | + | + | - | + | + | + | + | / | + | + | + | - | - | + | + | + | Low |
| Prat-Luri 2023 | + | + | - | - | + | + | + | / | + | - | + | - | - | + | + | + | Critically low |
| Prudencio 2023 | + | / | + | / | - | - | + | / | + | - | + | - | - | + | - | + | Critically low |
| Qiu 2022 | - | - | - | - | + | - | - | / | + | - | + | - | - | + | - | + | Critically low |
| Quentin 2021 | + | - | - | / | + | + | - | + | + | - | + | - | - | + | + | + | Critically low |
| Raj 2018 | - | - | - | - | - | - | - | / | + | - | - | - | - | - | - | - | Critically low |
| Ram 2023 | + | + | - | - | + | + | - | / | + | - | - | - | + | + | - | + | Critically low |
| Ranjan 2023 | + | / | - | - | + | + | - | - | + | - | + | - | - | + | + | + | Critically low |
| Regnaux 2015 | + | + | - | + | + | + | + | + | + | + | + | + | + | + | - | + | Low |
| Rocha 2020 | + | - | - | - | + | + | - | / | + | - | - | - | - | + | - | + | Critically low |
| Roddy 2005 | + | - | - | - | - | + | / | / | / | - | - | - | - | - | - | - | Critically low |
| Rogan 2018 | + | / | - | - | + | + | - | / | + | - | + | - | - | + | - | + | Critically low |
| Runge 2022 | + | + | - | / | + | + | - | / | + | - | + | + | + | + | - | + | Critically low |
| Salt 2011 | + | - | - | / | + | + | - | + | + | - | + | - | - | - | - | - | Critically low |
| Sampath 2015 | + | / | - | - | + | + | - | / | + | - | - | - | - | - | - | + | Critically low |
| Saragiotto 2016 | + | + | - | / | + | + | + | + | + | + | + | + | + | - | + | + | Moderate |
| Scali 2018 | + | / | - | - | + | + | - | / | - | - | - | - | - | + | + | + | Critically low |
| Schafer 2018 | + | / | - | - | + | + | - | + | + | + | + | - | - | - | - | + | Critically low |
| Searle 2015 | + | - | - | / | + | - | - | - | + | - | + | - | - | + | + | + | Critically low |
| Shanbehzadeh 2022 | - | / | - | - | + | + | - | / | + | - | + | - | - | + | + | + | Critically low |
| Sharma 2021 | + | - | - | - | + | + | - | + | + | - | - | - | - | - | - | + | Critically low |
| Shi 2018 | + | - | - | - | + | + | - | / | + | - | + | - | - | + | - | + | Critically low |
| Shi 2022 | + | + | - | / | - | + | - | / | + | - | - | - | - | - | + | + | Critically low |
| Shire 2017 | + | - | - | / | + | + | + | + | + | - | + | - | - | + | - | + | Critically low |
| Siddall 2021 | + | + | - | / | + | + | - | + | + | - | + | - | - | + | - | + | Critically low |
| Sieczkowska 2020 | + | / | - | / | - | + | - | / | + | - | + | - | - | + | + | + | Critically low |
| Silva 2020 | + | / | - | / | + | + | - | + | + | - | - | - | - | - | - | + | Critically low |
| Siriphorn 2020 | + | + | - | / | - | - | - | / | + | - | + | - | - | + | + | + | Critically low |
| Sitthipornvorakul 2018 | + | - | - | - | + | + | - | / | + | - | - | + | - | - | + | + | Critically low |
| Skelly 2020 | + | + | - | - | + | + | + | + | + | + | + | - | - | + | - | + | Critically low |
| Slade 2007 | + | - | - | - | - | - | - | / | + | - | - | - | - | - | - | + | Critically low |
| Smith 2012 | - | - | - | + | + | + | - | + | + | - | + | - | - | + | - | + | Critically low |
| Smith 2014 | + | - | - | - | + | + | + | / | + | - | + | + | + | + | - | + | Critically low |
| Smith 2017 | + | / | - | - | - | + | - | + | + | - | + | + | + | + | - | + | Critically low |
| Sobue 2022 | + | - | - | - | + | - | - | / | + | - | - | - | - | - | + | + | Critically low |
| Song 2022 | + | - | - | - | + | - | - | / | + | - | + | - | - | + | - | + | Critically low |
| Sosa-Reina 2017 | + | - | - | / | + | + | - | / | + | - | + | - | - | + | + | + | Critically low |
| Steuri 2017 | + | / | - | - | + | + | - | / | + | - | + | + | + | + | + | + | Critically low |
| Sun 2021 | + | - | - | - | + | + | - | - | + | - | + | - | - | + | + | + | Critically low |
| Sussmilch-Leitch 2012 | + | - | - | - | + | - | - | / | + | - | - | - | - | - | - | + | Critically low |
| Sutanto 2022 | + | / | - | / | + | - | - | + | + | - | + | + | + | + | - | + | Critically low |
| Tanaka 2013 (a) | + | - | - | - | + | - | - | - | + | - | - | - | - | - | - | - | Critically low |
| Tanaka 2013 (b) | + | - | - | / | + | + | - | / | + | - | - | - | - | - | + | + | Critically low |
| Tanaka 2014 | + | - | - | - | + | - | - | / | + | - | + | - | - | + | - | - | Critically low |
| Tataryn 2021 | + | / | - | - | + | + | - | + | + | - | + | - | - | + | + | + | Critically low |
| Temporiti 2022 | + | - | - | - | + | - | - | + | + | - | - | - | - | - | - | + | Critically low |
| Thomas 2022 | + | / | - | - | + | - | - | + | + | - | - | - | - | - | - | + | Critically low |
| Thompson 2023 | + | / | - | / | + | + | - | + | + | + | + | + | + | + | + | + | Low |
| Thornton 2021 | - | / | - | / | + | + | - | - | + | - | + | - | - | + | - | + | Critically low |
| Tomazoni 2020 | + | - | - | / | + | + | + | / | + | - | + | + | + | + | - | + | Critically low |
| Tsiringakis 2020 | + | - | - | + | + | + | + | / | + | - | - | - | - | - | + | + | Critically low |
| vanMiddelkoop 2011 | + | - | - | / | + | + | - | / | + | - | + | + | + | + | - | + | Critically low |
| Vanti 2019 | + | - | - | / | + | + | - | / | + | - | - | - | - | - | - | + | Critically low |
| Verhagen 2013 | + | / | - | + | + | + | + | + | + | - | + | + | + | + | + | + | Moderate |
| Vilarino 2022 | + | / | - | / | + | + | - | / | + | - | - | - | - | - | - | + | Critically low |
| Villanueva-Ruiz 2022 | + | / | - | / | + | - | - | + | + | - | + | + | + | + | - | + | Critically low |
| Waller 2014 | + | - | - | - | + | + | - | / | + | - | - | - | - | - | - | + | Critically low |
| Wang 2012 (a) | + | - | - | - | + | + | - | / | + | - | + | - | - | + | - | + | Critically low |
| Wang 2012 (b) | + | / | - | / | + | + | - | / | + | - | + | - | - | + | - | + | Critically low |
| Wang 2018 | + | / | - | - | + | + | - | / | - | - | - | - | - | + | + | + | Critically low |
| Wang 2021 (a) | + | / | - | + | + | + | - | / | + | - | + | - | - | + | + | + | Critically low |
| Wang 2021 (b) | + | + | - | - | + | + | - | / | + | - | + | - | - | + | + | + | Critically low |
| Wen 2022 | + | + | - | / | + | + | - | / | + | - | + | + | + | + | + | + | Low |
| Weng 2023 | + | / | - | / | + | + | - | / | + | + | + | - | - | + | + | + | Critically low |
| Wewege 2018 | + | - | - | - | + | + | - | / | + | - | + | - | - | + | + | + | Critically low |
| Wieland 2022 | + | / | - | + | + | + | + | + | + | + | + | + | + | + | + | + | High |
| Wilhelm 2020 | + | + | - | - | + | + | - | + | + | - | + | - | - | + | + | + | Critically low |
| Williams 2018 | + | + | - | + | + | + | + | + | + | + | + | + | + | + | - | + | Low |
| Wilson 2018 | + | + | - | + | + | + | - | / | + | - | + | - | - | + | - | + | Critically low |
| Winters 2020 | + | + | - | / | + | + | + | + | + | + | + | - | - | + | - | + | Critically low |
| Wood 2021 | + | + | - | / | + | + | - | + | + | - | + | - | - | + | + | + | Critically low |
| Wood 2022 | + | / | - | / | + | + | - | - | + | - | + | - | - | + | + | + | Critically low |
| Wu 2020 | + | - | - | / | - | - | - | / | / | - | + | - | - | + | - | + | Critically low |
| Wu 2022 (a) | + | / | - | - | + | + | / | / | + | - | + | - | - | + | + | - | Critically low |
| Wu 2022 (b) | + | / | - | / | + | + | + | / | + | - | - | - | - | - | + | + | Critically low |
| Wu 2023 | + | / | - | / | + | + | - | / | + | - | - | - | - | - | - | + | Critically low |
| Xie 2021 | + | / | - | - | + | - | - | / | + | - | - | - | - | + | - | + | Critically low |
| Xu 2023 | + | / | - | - | + | + | - | / | + | - | - | + | + | - | + | + | Critically low |
| Yamato 2015 | + | + | - | / | + | + | + | / | + | + | + | - | - | + | - | + | Critically low |
| Yan 2013 | + | - | - | / | + | + | - | + | / | - | + | - | - | + | + | + | Critically low |
| Yang 2022 (a) | + | - | - | - | + | + | - | / | + | - | + | + | + | - | - | + | Critically low |
| Yang 2022 (b) | + | - | - | / | + | - | - | / | + | - | + | + | + | + | + | + | Critically low |
| Ye 2022 | + | / | - | - | + | + | - | / | + | - | + | - | - | + | - | + | Critically low |
| Yoon 2021 | + | - | - | / | + | - | - | + | + | - | + | - | - | + | - | + | Critically low |
| Yue 2014 | + | - | - | + | + | + | - | + | + | + | + | - | - | + | - | + | Critically low |
| Zampogna 2020 | + | - | - | / | + | + | - | + | + | - | + | - | + | + | - | + | Critically low |
| Zeng 2020 | + | / | - | - | + | + | - | / | + | - | + | + | + | + | + | + | Critically low |
| Zhang 2017 | + | - | - | - | + | + | + | + | + | - | + | - | - | + | - | + | Critically low |
| Zhang 2019 (a) | + | / | - | / | + | + | + | + | + | - | + | + | + | + | + | + | Moderate |
| Zhang 2019 (b) | + | - | - | / | + | + | - | / | + | - | + | + | + | + | + | + | Critically low |
| Zhang 2021 | + | - | - | - | + | + | - | / | + | - | + | - | - | + | + | + | Critically low |
| Zhang 2022 (a) | + | / | - | / | + | + | - | / | + | - | - | - | - | + | + | + | Critically low |
| Zhang 2022 (b) | + | - | - | / | - | - | - | / | + | - | - | - | - | + | + | + | Critically low |
| Zhang 2022 (c) | - | - | - | - | - | - | - | / | + | - | + | - | - | + | - | + | Critically low |
| Zhu 2020 | + | + | - | / | + | + | - | / | + | - | + | - | - | + | + | + | Critically low |
| Zou 2019 | + | - | - | / | + | + | - | / | + | - | + | - | - | + | + | + | Critically low |

+ Yes, / Partial yes, - No

# Supplementary file 8. Narrative summary of the included reviews (per diagnosis)

**Lower back pain**

72 systematic reviews that performed meta-analysis on 1664 RCTs were included for different pain conditions like low back pain, lumbar disc herniation, lumbar spondylosis, sciatica or lumbar spinal stenosis.[155–216,218–226] Out of these, 45 reviews studied chronic low back pain and 27 reviews summarized evidence from RCTs of mixed chronicity (i.e., acute, subacute and chronic). Majority of back pain reviews studied the effect of a combination of exercise interventions. For reviews of individual exercise training, mind-body therapies (pilates, yoga, Tai chi, Baduanjin) and stabilization exercises were the most commonly studied exercise interventions followed by aerobic exercises, proprioceptive neuromuscular facilitation exercises, resistance exercises, and water-based exercises. Only 7 reviews compared the effects of exercise with exercise and 2 reviews compared exercise to no treatment or usual care control. Three of the included reviews reported on all the dose variables (frequency, intensity, volume, and duration). Seven reviews did not report on any of the dose variables. Most of the reviews analysed pain (N=71) and physical function (N=68). However, mental health (N=5) and adverse events (N=3) were the least analyzed outcomes. None of the low back pain reviews analysed the relationship between exercise intervention and adherence rate.

**Knee pain**

Out of the 65 knee pain reviews [90–154](1157 RCTs in meta-analysis), 48 reviews included RCTs of knee osteoarthritis (OA) and 14 reviews included RCTs of patellofemoral pain and one review included RCTs with patellar tendinopathy. Frequency was reported in majority of the studies (57/65) followed by duration (63/65) with only 25 and 16 studies reporting intensity and volume respectively. Fifteen studies reported on all the exercise prescription variables. Pain intensity was analysed in all the included reviews. Physical function was analysed in 55 reviews followed by mental health (6/65), adherence (3/65) and adverse events (4/65).

**Combination of chronic musculoskeletal disorders**

48 systematic reviews (n=1263 studies) analysed the role of exercise interventions in RCTs of a combination of different musculoskeletal disorders like low back pain, neck pain, osteoarthritis, fibromyalgia, rheumatoid arthritis, shoulder pain, hand pain and upper and lower limb tendinopathy.[29–76] Out of these, 42 reviews summarized RCTs on chronic pain conditions and six of the reviews had RCTs with mixed duration of chronicity. Most of the reviews reported on the duration and frequency of exercise interventions. However, only a few reviews reported the intensity (N=10) and volume (N=8) variables. Most of the reviews analysed the relationship of exercise interventions for difference in pain intensity (N=45), physical function (N=36). A small proportion of reviews mental health (N=9), adverse events (N=3), and adherence rates (N=1).

**Neck pain**

23 systematic reviews (n=344 studies) analysed the role of exercise in different conditions like non-specific neck pain (N=10 reviews), whiplash associated disorders (N=2 reviews), mechanical neck disorders (N=1 review), cervical radiculopathy (N=1 review), combination of neck pain conditions (N=9 reviews).[227–249] Seven reviews addressed chronic neck pain and sixteen reviews had mixed duration of chronicity. Most of the reviews reported duration (N=21) and frequency (N=20) of exercise interventions. Only a few reviews reported on intensity (N=4) and volume (N=4) of training. Pain (N=23) and physical function (N=19) were the most commonly analysed variables, whereas only five reviews analysed the role of exercise on mental health and none of the reviews analysed adverse events and adherence rates.

**Chronic widespread pain**

22 reviews (n=525 RCTs in meta-analysis) analysed the role of exercise interventions in chronic widespread pain conditions like fibromyalgia (n=20) and myofascial pain syndrome (n=2).[7–28] Most of the reviews reported on the frequency (20/22) and duration (21/22) of exercise interventions but only 8 reported the intensity and 10 reported volume of training. A major proportion of reviews analysed the effects of exercise on pain, physical function and mental health. However, only three of the reviews analysed adherence rate and none of these analysed the effects of exercise on adverse events in chronic widespread pain.

**Shoulder pain**

Seventeen reviews analysed evidence from 434 RCTs for musculoskeletal disorders affecting the shoulder like frozen shoulder (n=1), rotator cuff tendinopathies (n=2), subacromial impingement syndrome (n=9) and mixed shoulder pain conditions (n=5).[258–274] Of these, fifteen reviews reported on duration, fourteen on frequency, three on intensity and three on volume of exercise interventions. Three reviews did not report on any exercise prescription variable. All the reviews analysed pain, seventeen reported on function and only one on mental health. None of the reviews analysed the effect of exercise intervention on adverse events and adherence rate.

**Rheumatoid arthritis**

Eight reviews (n=77 RCTs) analysed data for effect of exercise interventions on rheumatoid arthritis.[250–257] All the reviews reported on duration of exercises, 7 reported on frequency of training and only 4 reported on intensity of exercises. Only one review reported the volume of training. Pain and function were analysed in all these reviews but only one of the reviews analysed data for mental health. Adherence rates were also analysed in 4 reviews whereas only 2 reviews analysed adverse events.

**Hip pain**

Seven systematic reviews (n=65 studies) analysed the effects of exercise interventions on hip osteoarthritis.[83–89] All the included reviews reported on frequency and duration of exercise interventions but only two reported the intensity of training and a single review reported on volume of exercise training. All seven reviews analysed pain outcome, five analysed physical function, one analysed adherence rate but none of the reviews analysed mental health or adverse events following exercise interventions. None of the reviews analysed the role of exercise prescription dose in hip osteoarthritis.

**Ankle and foot pain**

Six reviews (n=52 studies) analysed the effect of exercise interventions on achilles tendinopathy (N=5) and plantar fasciitis (N=1).[1–6]All the reviews reported the duration and four of the reviews reported the frequency of the exercise intervention. Volume was reported in three reviews and none of the reviews reported the intensity of exercise training. All the reviews analysed pain, three reviews analysed physical function but none of the included reviews analysed the effect of exercise interventions on mental health, adverse events and adherence rates.

**Elbow pain**

Three systematic reviews (n=50 studies) analysed the effects of exercise interventions on lateral elbow tendinopathy.[77–79] Two of the studies reported on the frequency, duration and volume of exercise interventions. All the reviews analysed pain and two of these analysed physical function but none of the reviews analysed the relationship of exercise with mental health, adverse events and adherence rates. None of the included reviews analysed the impact of exercise prescription dose on lateral elbow tendinopathy.

**Hand pain**

Three systematic reviews (n=23 studies) analysed the effectiveness of exercise interventions in hand osteoarthritis (N=2) and carpometacarpal osteoarthritis (N=1).[80–82] All three reviews reported on frequency and duration, two reported on intensity and only one reported on exercise volume. All the included reviews analysed the effects of exercise on pain and physical function outcomes but none of these analysed mental health, adverse events or adherence rates. Moreover, none of the reviews analysed the role of exercise dosage in musculoskeletal disorders of hand.

# Supplementary file 9. Narrative summary of the included reviews (exercise dose prescription per diagnosis)

**Lower back pain** *(Based on two low and nine critically low AMSTAR-2 quality reviews)* [167,173,190,194,196,201,202,208,210,219,226]

One review found that performing stabilization exercises 3-5 times per week for 20–30 minutes each has the greatest impact on pain and disability in people with chronic non-specific low back pain.[167] Other reviews showed that exercise training duration does not significantly alter the pain intensity in chronic non-specific low back pain (NSLBP).[173,196] A high-intensity exercise training was found to be beneficial for reducing disability and improving mental health in chronic individuals with NSLBP.[194,208] Meta-regression analyses also found no additional effect of pilates or session duration of yoga for pain and function in subjects with low back pain.[201,202] Longer-duration (>45 minutes) traditional Chinese exercise sessions performed three to four times per week were found to be beneficial for reducing pain intensity in subjects with low back pain.[190] A comparison of duration of proprioceptive neuromuscular facilitation exercise revealed that both shorter (upto four weeks) and longer duration (six to eight weeks) produced significant improvements in individuals with chronic low back pain.[210] Isometric exercises lasting more than eight weeks resulted in significant improvements in pain intensity, in contrast to shorter durations of training (less than eight weeks). However, no additional impact of exercise duration was found for improvement of disability following isometric training and for reduction of pain intensity following motor control exercises.[219] Exercise therapy, performed at least three times per week for a duration exceeding 12 weeks, demonstrated superiority over fewer than three sessions of less than 12 weeks for reducing pain and improving physical function in chronic low back pain.[226]

**Knee pain** *(Based on one low and seven critically low AMSTAR-2 quality reviews)* [91,99,101,104,105,124,130,147]

Evidence from a subgroup and meta-regression analysis of duration and frequency of exercise interventions in RCTs demonstrated resistance training (less than 8 or 12 weeks, >4 sessions per week),[91,101] aerobic training (less than 8 weeks, >2 sessions per week),[91,130] land based exercises (8 to 11 weeks, >3 sessions per week),[99] Tai chi (<24 weeks),[104] and Baduanjin (8 or 12 weeks) [124] were beneficial for reducing pain and improving function in knee osteoarthritis. The findings from a recent univariate meta-regression analysis also revealed a favorable association, indicating that longer intervention durations are associated with a small positive effect on reducing pain and enhancing physical function.[147] However, a systematic review and moderator analysis of a combination of exercise interventions did not demonstrate an additional impact of dose prescription on pain intensity in subjects with knee OA.[105] None of the reviews analysed the role of exercise dose on mental health, adverse effects or adherence rate.

**Shoulder pain** *(Based on one critically low AMSTAR-2 quality review)* [264]

Subjects with subacromial impingement syndrome experienced a significant decrease in pain intensity after performing eccentric exercises for a period of 12 weeks.[264] However, no significant improvements were observed in physical function. Conversely, when eccentric exercises were performed for 6-8 weeks, no statistically significant differences were found in both pain and physical function.

**Chronic widespread pain** *(Based on three critically low AMSTAR-2 quality reviews)*[17,18,26]

Aquatic exercise (1-3 session/week) at a self-selected intensity for a duration of more than seven weeks led to a significant reduction in pain intensity among individuals with fibromyalgia. Notably, a higher dose of exercise (three sessions per week for 12 weeks) was associated with greater improvements compared to a lower dose (once a week for 7 to 12 weeks).[17] Likewise, one review examining the effects of exercise combinations found that a higher dose of exercise (four sessions per week for more than 26 weeks) resulted in greater improvements in anxiety symptoms, compared to a lower dose of exercise training (one or three sessions per week for 13 to 26 weeks).[18] A combination of exercises performed for a duration of 13 to 26 weeks, at a frequency of 30 to 60 minutes a day, has also been shown to provide significant reduction in pain intensity for individuals with fibromyalgia.[26]

**Rheumatoid arthritis** *(Based on two critically low AMSTAR-2 quality reviews)*[250,251]

Aerobic exercises performed three or more times per week for a duration of upto 13 weeks lead to a significant improvements in pain intensity in individuals with rheumatoid arthritis.[250] Resistance exercises for less than six weeks performed twice a week at more than 80% intensity showed significant improvements in disability.[251]

**Neck pain** *(Based on two critically low AMSTAR-2 quality review)*[240,249]

A meta-regression analysis of RCTs demonstrated no significant effects of exercise dose (intervention duration and session duration) for the improving disability and reducing pain in individuals with neck pain.[240] Similarly, a subgroup analysis revealed a significant reduction in pain intensity and disability irrespective of the duration of isometric exercises (upto eight weeks vs more than eight weeks).[249]

**Ankle and foot pain** *(Based on one critically low AMSTAR-2 quality review)*[4]

No differences were observed between low and high intensity eccentric exercise training in improvement of physical function for individuals with Achilles tendinopathy.[4]

**Combination of chronic musculoskeletal disorders** *(Based on one low and one critically low AMSTAR-2 quality reviews)*[38,44]

Exercises performed more frequently with a shorter intervention and session duration demonstrated significant effects in a meta-regression analysis.[38] Tai Chi exercises for longer than 6 weeks produced significant improvements in pain intensity.[44]

# Supplementary file 10. Meta-regression of exercise dose in different pain conditions (Physical function outcome)

| Author Year | Exercise | Variables | Coefficient | CI | p-value | N of studies | R^2^ | Type of regression | Follow-up time | |
| --- | --- | --- | --- | --- | --- | --- | --- | --- | --- | --- |
| Aladro-Gonzalvo et al. 2013 | Pilates | Frequency | - | - | 0.398 | 4 | 0.363 | - | Short-term | |
| Session duration (minutes) | - | - | 0.773 | 4 | 0.051 | - | Short-term | |  |  |
| Quentin et al. 2021 | Home exercise training | Duration (weeks) | -1.57 | (-2.89 to -0.16) | 0.03 | - | - | - | Median time of follow-up | |
| Session duration (minutes) | -0.12 | (-0.41 to 0.14) | 0.33 | - | - | - | Median time of follow-up | |  |  |
| Frequency | 0.19 | (-1.01 to 1.39) | 0.74 | - | - | - | Median time of follow-up | |  |  |
| Anheyer et al. 2021 | Yoga | Intervention duration (hours) | 0.01 | (-0.01 to 0.02) | 0.21 | 15 | - | Mixed-effects model meta-regression | Short-term | |
| Pedersen et al. 2022 | Exercise therapy | Duration (weeks) | -0.04 | (-0.11 to 0.02) | - | - | - | Univariate meta-regression | - | |
| Mueller and Niederer 2020 | Stabilization exercises | Duration (weeks) | 0.1 | (-0.3 to 0.95) | 0.3 | 37 | 0.15 | Sensitivity meta-regression | Post intervention | |
| Frequency | 0.26 | (-0.61 to 1.1) | 0.6 | 37 | 0.15 | Sensitivity meta-regression | Post intervention | |  |  |
| Session duration (minutes) | -1 | (-3.1 to 0.95) | 0.3 | 37 | 0.15 | Sensitivity meta-regression | Post intervention | |  |  |
| Wilhelm et al. 2020 | Exercise therapy | Duration (weeks) | 0.01 | (-0.03 to 0.07) | - | 7 |  | - | - |  |
|  | | Intervention duration (minutes per week) | 0 | (0.00 to 0.01) | - | 7 |  | - | - |  |

All the coefficients are scaled in the same direction of effect. – (minus) indicates an improvement in outcome measures. Abbreviations: CI, confidence interval; N, number.

# Supplementary file 11. Meta-regression of exercise dose in different pain conditions (pain outcome)

| Author Year | Exercise | Variables | Coefficient | CI | p value | N of studies | R² | Type of regression | Follow-up time |
| --- | --- | --- | --- | --- | --- | --- | --- | --- | --- |
| Aladro-Gonzalvo et al. 2013 | Pilates | Frequency | - | - | 0.438 | 5 | 0.21 | - | Short-term |
| Quentin et al. 2021 | Home exercise training | Duration (weeks) | 0.12 | (-0.01 to 0.2) | 0.07 | - | - | - | Median time of follow-up |
| Session duration (minutes) | 0.03 | (-0.02 to 0.08) | 0.24 | - | - | - | Median time of follow-up |  |  |
| Frequency | 0.93 | (0.03 to 1.80) | 0.04 | - | - | - | Median time of follow-up |  |  |
| Anheyer et al. 2021 | Yoga | Intervention duration (hours) | 0.002 | (-0.01 to 0.01) | 0.75 | 15 | - | Mixed-effects model meta-regression | Short-term |
| Pedersen et al. 2022 | Exercise therapy | Duration (weeks) | -0.03 | (-0.07 to 0.00) | - | - | - | Univariate meta-regression | - |
| Yang et al. 2022 | Isometric training | Duration (weeks) | 0.03 | (-0.15 to 0.21) | 0.708 | - | - | Meta-regression | - |
| Mueller and Niederer 2020 | Stabilization exercises | Duration (weeks) | -0.009 | (-0.1 to 0.08) | 0.8 | 40 | 0.445 | Sensitivity meta-regression | Post intervention |
| Frequency | 0.164 | (-0.239 to 0.567) | 0.4 | 40 | 0.445 | Sensitivity meta-regression | Post intervention |  |  |
| Session duration (minutes) | -1.75 | (-2.61 to -0.879) | 0.0001 | 40 | 0.445 | Sensitivity meta-regression | Post intervention |  |  |
| Niederer and Mueller 2020 | Motor control stabilization exercises | Duration  (weeks) | -0.09 | (-0.22 to 0.03) | 0.15 | 8 | - | Sensitivity meta-regression | Post intervention |
| Frequency | 0.0007 | (-0.11 to 0.11) | 0.99 | 8 | - | Sensitivity meta-regression | Post intervention |  |  |
| Session duration (minutes) | -0.0004 | (-0.001 to 0.0004) | 0.35 | 8 | - | Sensitivity meta-regression | Post intervention |  |  |
| Wilhelm et al. 2020 | Exercise therapy | Duration (weeks) | 0 | (-0.05 to 0.04) | - | 13 | - | - | - |
|  | | Intervention duration (minutes per week) | 0 | (0.00 to 0.00) | - | 13 | - | - | - |

All the coefficients are scaled in the same direction of effect. – (minus) indicates an improvement in outcome measures. Abbreviations: CI, confidence interval; N, number.

# Supplementary file 12a. Heat map for primary study overlap analysis





Abbreviations: RA, rheumatoid arthritis; SP, shoulder pain; NP, neck pain; LBP, low back pain; KP, knee pain; FM, fibromyalgia; Com, combination; AT, Achilles tendinopathy. Citation matrix demonstrating pairwise overlap of primary studies in reviews of different pain conditions

# Supplementary file 12b. Sequential pairwise comparisons for primary study overlap analysis

| Reviews | CCA Percentage | Degree of overlap |
| --- | --- | --- |
| (KP) Li 2016 vs. (KP) Tanaka 2014 | 37.5 | Very high |
| (KP) Imoto 2019 vs. (KP) Juhl 2014 | 31.6 | Very high |
| (KP) Hu 2021 vs. (KP) Li 2020 | 30.4 | Very high |
| (KP) Juhl 2014 vs. (KP) Tanaka 2014 | 25.0 | Very high |
| (KP) Juhl 2014 vs. (KP) Li 2016 | 23.1 | Very high |
| (Com) Kong 2016 vs. (KP) Hu 2021 | 22.2 | Very high |
| (KP) Imoto 2019 vs. (KP) Tanaka 2014 | 21.7 | Very high |
| (KP) Juhl 2014 vs. (KP) Pedersen 2022 | 21.7 | Very high |
| (FM) Bidonde 2014 vs. (FM) McDowell 2017 | 18.2 | Very high |
| (LBP) Mueller 2020 vs. (LBP) Niederer 2020 | 17.6 | Very high |
| (KP) Jansen 2011 vs. (KP) Li 2016 | 16.7 | Very high |
| (KP) Imoto 2019 vs. (KP) Li 2016 | 16.1 | Very high |
| (KP) Jansen 2011 vs. (KP) Juhl 2014 | 15.4 | Very high |
| (KP) Imoto 2019 vs. (KP) Jansen 2011 | 15.3 | Very high |
| (Com) Kong 2016 vs. (KP) Li 2020 | 14.8 | High |
| (KP) Imoto 2019 vs. (KP) Pedersen 2022 | 13.6 | High |
| (KP) Li 2016 vs. (KP) Pedersen 2022 | 13.0 | High |
| (FM) Albuquerque 2022 vs. (FM) McDowell 2017 | 12.0 | High |
| (Com) Polaski 2019 vs. (KP) Imoto 2019 | 11.6 | High |
| (KP) Jansen 2011 vs. (KP) Tanaka 2014 | 11.5 | High |
| (KP) Li 2020 vs. (KP) Tanaka 2014 | 10.7 | High |
| (KP) Pedersen 2022 vs. (KP) Tanaka 2014 | 10.4 | High |
| (LBP) Ram 2023 vs. (LBP) Wewege 2018 | 10.0 | Moderate |
| (FM) Albuquerque 2022 vs. (FM) Bidonde 2014 | 9.7 | Moderate |
| (Com) Polaski 2019 vs. (KP) Juhl 2014 | 8.3 | Moderate |
| (Com) Polaski 2019 vs. (FM) Bidonde 2014 | 7.6 | Moderate |
| (Com) Polaski 2019 vs. (KP) Tanaka 2014 | 7.5 | Moderate |
| (KP) Hu 2021 vs. (KP) Imoto 2019 | 7.5 | Moderate |
| (LBP) Zhang 2019 vs. (LBP) Zhang 2022 | 7.4 | Moderate |
| (LBP) Mueller 2020 vs. (LBP) Sutanto 2022 | 6.8 | Moderate |
| (KP) Hu 2021 vs. (KP) Tanaka 2014 | 6.5 | Moderate |
| (Com) Kong 2016 vs. (KP) Tanaka 2014 | 6.2 | Moderate |
| (KP) Imoto 2019 vs. (KP) Li 2020 | 6.1 | Moderate |
| (LBP) Niederer 2020 vs. (LBP) Sutanto 2022 | 5.9 | Moderate |
| (Com) Kong 2016 vs. (KP) Imoto 2019 | 5.8 | Moderate |
| (Com) Polaski 2019 vs. (FM) McDowell 2017 | 5.3 | Moderate |
| (LBP) Anheyer 2021 vs. (LBP) Quentin 2021 | 5.0 | Slight |
| (Com) Polaski 2019 vs. (KP) Li 2016 | 4.9 | Slight |
| (Com) Polaski 2019 vs. (FM) Albuquerque 2022 | 4.8 | Slight |
| (LBP) Anheyer 2021 vs. (LBP) Zhang 2022 | 4.3 | Slight |
| (RA) Baillet 2010 vs. (RA) Baillet 2012 | 4.3 | Slight |
| (Com) Polaski 2019 vs. (LBP) Aladro-Gonzalvo 2013 | 4.0 | Slight |
| (LBP) Gao 2022 vs. (LBP) Zhang 2022 | 3.6 | Slight |
| (LBP) Gao 2022 vs. (LBP) Mueller 2020 | 3.4 | Slight |
| (KP) Juhl 2014 vs. (KP) Li 2020 | 3.3 | Slight |
| (KP) Hu 2021 vs. (KP) Juhl 2014 | 3.2 | Slight |
| (KP) Hu 2021 vs. (KP) Li 2016 | 3.2 | Slight |
| (NP) Wilhelm 2020 vs. (NP) Yang 2022 | 3.2 | Slight |
| (Com) Polaski 2019 vs. (KP) Pedersen 2022 | 2.9 | Slight |
| (Com) Polaski 2019 vs. (KP) Li 2020 | 2.5 | Slight |
| (Com) Polaski 2019 vs. (RA) Baillet 2010 | 2.5 | Slight |
| (LBP) Anheyer 2021 vs. (LBP) Zhang 2019 | 2.5 | Slight |
| (LBP) Mueller 2020 vs. (LBP) Quentin 2021 | 2.5 | Slight |
| (Com) Kong 2016 vs. (Com) Polaski 2019 | 2.4 | Slight |
| (KP) Jansen 2011 vs. (KP) Pedersen 2022 | 2.1 | Slight |
| (Com) Kong 2016 vs. (LBP) Sutanto 2022 | 1.7 | Slight |
| (Com) Polaski 2019 vs. (LBP) Mueller 2020 | 1.7 | Slight |
| (LBP) Mueller 2020 vs. (LBP) Zhang 2019 | 1.7 | Slight |
| (Com) Kong 2016 vs. (KP) Juhl 2014 | 1.6 | Slight |
| (LBP) Sutanto 2022 vs. (LBP) Zhang 2022 | 1.6 | Slight |
| (LBP) Mueller 2020 vs. (LBP) Zhang 2022 | 1.5 | Slight |
| (Com) Polaski 2019 vs. (LBP) Niederer 2020 | 1.3 | Slight |
| (LBP) Anheyer 2021 vs. (LBP) Mueller 2020 | 1.3 | Slight |
| (Com) Polaski 2019 vs. (KP) Hu 2021 | 1.2 | Slight |
| (Com) Polaski 2019 vs. (KP) Jansen 2011 | 1.2 | Slight |
| (Com) Polaski 2019 vs. (LBP) Sutanto 2022 | 0.9 | Slight |
| (AT) Wilson 2018 vs. (Com) Kong 2016 | 0.0 | Slight |
| (AT) Wilson 2018 vs. (Com) Polaski 2019 | 0.0 | Slight |
| (AT) Wilson 2018 vs. (FM) Albuquerque 2022 | 0.0 | Slight |
| (AT) Wilson 2018 vs. (FM) Bidonde 2014 | 0.0 | Slight |
| (AT) Wilson 2018 vs. (FM) McDowell 2017 | 0.0 | Slight |
| (AT) Wilson 2018 vs. (KP) Hu 2021 | 0.0 | Slight |
| (AT) Wilson 2018 vs. (KP) Imoto 2019 | 0.0 | Slight |
| (AT) Wilson 2018 vs. (KP) Jansen 2011 | 0.0 | Slight |
| (AT) Wilson 2018 vs. (KP) Juhl 2014 | 0.0 | Slight |
| (AT) Wilson 2018 vs. (KP) Li 2016 | 0.0 | Slight |
| (AT) Wilson 2018 vs. (KP) Li 2020 | 0.0 | Slight |
| (AT) Wilson 2018 vs. (KP) Pedersen 2022 | 0.0 | Slight |
| (AT) Wilson 2018 vs. (KP) Tanaka 2014 | 0.0 | Slight |
| (AT) Wilson 2018 vs. (LBP) Aladro-Gonzalvo 2013 | 0.0 | Slight |
| (AT) Wilson 2018 vs. (LBP) Anheyer 2021 | 0.0 | Slight |
| (AT) Wilson 2018 vs. (LBP) Gao 2022 | 0.0 | Slight |
| (AT) Wilson 2018 vs. (LBP) Mueller 2020 | 0.0 | Slight |
| (AT) Wilson 2018 vs. (LBP) Niederer 2020 | 0.0 | Slight |
| (AT) Wilson 2018 vs. (LBP) Quentin 2021 | 0.0 | Slight |
| (AT) Wilson 2018 vs. (LBP) Ram 2023 | 0.0 | Slight |
| (AT) Wilson 2018 vs. (LBP) Sutanto 2022 | 0.0 | Slight |
| (AT) Wilson 2018 vs. (LBP) Wewege 2018 | 0.0 | Slight |
| (AT) Wilson 2018 vs. (LBP) Zhang 2019 | 0.0 | Slight |
| (AT) Wilson 2018 vs. (LBP) Zhang 2022 | 0.0 | Slight |
| (AT) Wilson 2018 vs. (NP) Wilhelm 2020 | 0.0 | Slight |
| (AT) Wilson 2018 vs. (NP) Yang 2022 | 0.0 | Slight |
| (AT) Wilson 2018 vs. (RA) Baillet 2010 | 0.0 | Slight |
| (AT) Wilson 2018 vs. (RA) Baillet 2012 | 0.0 | Slight |
| (AT) Wilson 2018 vs. (SP) Larsson 2019 | 0.0 | Slight |
| (Com) Kong 2016 vs. (FM) Albuquerque 2022 | 0.0 | Slight |
| (Com) Kong 2016 vs. (FM) Bidonde 2014 | 0.0 | Slight |
| (Com) Kong 2016 vs. (FM) McDowell 2017 | 0.0 | Slight |
| (Com) Kong 2016 vs. (KP) Jansen 2011 | 0.0 | Slight |
| (Com) Kong 2016 vs. (KP) Li 2016 | 0.0 | Slight |
| (Com) Kong 2016 vs. (KP) Pedersen 2022 | 0.0 | Slight |
| (Com) Kong 2016 vs. (LBP) Aladro-Gonzalvo 2013 | 0.0 | Slight |
| (Com) Kong 2016 vs. (LBP) Anheyer 2021 | 0.0 | Slight |
| (Com) Kong 2016 vs. (LBP) Gao 2022 | 0.0 | Slight |
| (Com) Kong 2016 vs. (LBP) Mueller 2020 | 0.0 | Slight |
| (Com) Kong 2016 vs. (LBP) Niederer 2020 | 0.0 | Slight |
| (Com) Kong 2016 vs. (LBP) Quentin 2021 | 0.0 | Slight |
| (Com) Kong 2016 vs. (LBP) Ram 2023 | 0.0 | Slight |
| (Com) Kong 2016 vs. (LBP) Wewege 2018 | 0.0 | Slight |
| (Com) Kong 2016 vs. (LBP) Zhang 2019 | 0.0 | Slight |
| (Com) Kong 2016 vs. (LBP) Zhang 2022 | 0.0 | Slight |
| (Com) Kong 2016 vs. (NP) Wilhelm 2020 | 0.0 | Slight |
| (Com) Kong 2016 vs. (NP) Yang 2022 | 0.0 | Slight |
| (Com) Kong 2016 vs. (RA) Baillet 2010 | 0.0 | Slight |
| (Com) Kong 2016 vs. (RA) Baillet 2012 | 0.0 | Slight |
| (Com) Kong 2016 vs. (SP) Larsson 2019 | 0.0 | Slight |
| (Com) Polaski 2019 vs. (LBP) Anheyer 2021 | 0.0 | Slight |
| (Com) Polaski 2019 vs. (LBP) Gao 2022 | 0.0 | Slight |
| (Com) Polaski 2019 vs. (LBP) Quentin 2021 | 0.0 | Slight |
| (Com) Polaski 2019 vs. (LBP) Ram 2023 | 0.0 | Slight |
| (Com) Polaski 2019 vs. (LBP) Wewege 2018 | 0.0 | Slight |
| (Com) Polaski 2019 vs. (LBP) Zhang 2019 | 0.0 | Slight |
| (Com) Polaski 2019 vs. (LBP) Zhang 2022 | 0.0 | Slight |
| (Com) Polaski 2019 vs. (NP) Wilhelm 2020 | 0.0 | Slight |
| (Com) Polaski 2019 vs. (NP) Yang 2022 | 0.0 | Slight |
| (Com) Polaski 2019 vs. (RA) Baillet 2012 | 0.0 | Slight |
| (Com) Polaski 2019 vs. (SP) Larsson 2019 | 0.0 | Slight |
| (FM) Albuquerque 2022 vs. (KP) Hu 2021 | 0.0 | Slight |
| (FM) Albuquerque 2022 vs. (KP) Imoto 2019 | 0.0 | Slight |
| (FM) Albuquerque 2022 vs. (KP) Jansen 2011 | 0.0 | Slight |
| (FM) Albuquerque 2022 vs. (KP) Juhl 2014 | 0.0 | Slight |
| (FM) Albuquerque 2022 vs. (KP) Li 2016 | 0.0 | Slight |
| (FM) Albuquerque 2022 vs. (KP) Li 2020 | 0.0 | Slight |
| (FM) Albuquerque 2022 vs. (KP) Pedersen 2022 | 0.0 | Slight |
| (FM) Albuquerque 2022 vs. (KP) Tanaka 2014 | 0.0 | Slight |
| (FM) Albuquerque 2022 vs. (LBP) Aladro-Gonzalvo 2013 | 0.0 | Slight |
| (FM) Albuquerque 2022 vs. (LBP) Anheyer 2021 | 0.0 | Slight |
| (FM) Albuquerque 2022 vs. (LBP) Gao 2022 | 0.0 | Slight |
| (FM) Albuquerque 2022 vs. (LBP) Mueller 2020 | 0.0 | Slight |
| (FM) Albuquerque 2022 vs. (LBP) Niederer 2020 | 0.0 | Slight |
| (FM) Albuquerque 2022 vs. (LBP) Quentin 2021 | 0.0 | Slight |
| (FM) Albuquerque 2022 vs. (LBP) Ram 2023 | 0.0 | Slight |
| (FM) Albuquerque 2022 vs. (LBP) Sutanto 2022 | 0.0 | Slight |
| (FM) Albuquerque 2022 vs. (LBP) Wewege 2018 | 0.0 | Slight |
| (FM) Albuquerque 2022 vs. (LBP) Zhang 2019 | 0.0 | Slight |
| (FM) Albuquerque 2022 vs. (LBP) Zhang 2022 | 0.0 | Slight |
| (FM) Albuquerque 2022 vs. (NP) Wilhelm 2020 | 0.0 | Slight |
| (FM) Albuquerque 2022 vs. (NP) Yang 2022 | 0.0 | Slight |
| (FM) Albuquerque 2022 vs. (RA) Baillet 2010 | 0.0 | Slight |
| (FM) Albuquerque 2022 vs. (RA) Baillet 2012 | 0.0 | Slight |
| (FM) Albuquerque 2022 vs. (SP) Larsson 2019 | 0.0 | Slight |
| (FM) Bidonde 2014 vs. (KP) Hu 2021 | 0.0 | Slight |
| (FM) Bidonde 2014 vs. (KP) Imoto 2019 | 0.0 | Slight |
| (FM) Bidonde 2014 vs. (KP) Jansen 2011 | 0.0 | Slight |
| (FM) Bidonde 2014 vs. (KP) Juhl 2014 | 0.0 | Slight |
| (FM) Bidonde 2014 vs. (KP) Li 2016 | 0.0 | Slight |
| (FM) Bidonde 2014 vs. (KP) Li 2020 | 0.0 | Slight |
| (FM) Bidonde 2014 vs. (KP) Pedersen 2022 | 0.0 | Slight |
| (FM) Bidonde 2014 vs. (KP) Tanaka 2014 | 0.0 | Slight |
| (FM) Bidonde 2014 vs. (LBP) Aladro-Gonzalvo 2013 | 0.0 | Slight |
| (FM) Bidonde 2014 vs. (LBP) Anheyer 2021 | 0.0 | Slight |
| (FM) Bidonde 2014 vs. (LBP) Gao 2022 | 0.0 | Slight |
| (FM) Bidonde 2014 vs. (LBP) Mueller 2020 | 0.0 | Slight |
| (FM) Bidonde 2014 vs. (LBP) Niederer 2020 | 0.0 | Slight |
| (FM) Bidonde 2014 vs. (LBP) Quentin 2021 | 0.0 | Slight |
| (FM) Bidonde 2014 vs. (LBP) Ram 2023 | 0.0 | Slight |
| (FM) Bidonde 2014 vs. (LBP) Sutanto 2022 | 0.0 | Slight |
| (FM) Bidonde 2014 vs. (LBP) Wewege 2018 | 0.0 | Slight |
| (FM) Bidonde 2014 vs. (LBP) Zhang 2019 | 0.0 | Slight |
| (FM) Bidonde 2014 vs. (LBP) Zhang 2022 | 0.0 | Slight |
| (FM) Bidonde 2014 vs. (NP) Wilhelm 2020 | 0.0 | Slight |
| (FM) Bidonde 2014 vs. (NP) Yang 2022 | 0.0 | Slight |
| (FM) Bidonde 2014 vs. (RA) Baillet 2010 | 0.0 | Slight |
| (FM) Bidonde 2014 vs. (RA) Baillet 2012 | 0.0 | Slight |
| (FM) Bidonde 2014 vs. (SP) Larsson 2019 | 0.0 | Slight |
| (FM) McDowell 2017 vs. (KP) Hu 2021 | 0.0 | Slight |
| (FM) McDowell 2017 vs. (KP) Imoto 2019 | 0.0 | Slight |
| (FM) McDowell 2017 vs. (KP) Jansen 2011 | 0.0 | Slight |
| (FM) McDowell 2017 vs. (KP) Juhl 2014 | 0.0 | Slight |
| (FM) McDowell 2017 vs. (KP) Li 2016 | 0.0 | Slight |
| (FM) McDowell 2017 vs. (KP) Li 2020 | 0.0 | Slight |
| (FM) McDowell 2017 vs. (KP) Pedersen 2022 | 0.0 | Slight |
| (FM) McDowell 2017 vs. (KP) Tanaka 2014 | 0.0 | Slight |
| (FM) McDowell 2017 vs. (LBP) Aladro-Gonzalvo 2013 | 0.0 | Slight |
| (FM) McDowell 2017 vs. (LBP) Anheyer 2021 | 0.0 | Slight |
| (FM) McDowell 2017 vs. (LBP) Gao 2022 | 0.0 | Slight |
| (FM) McDowell 2017 vs. (LBP) Mueller 2020 | 0.0 | Slight |
| (FM) McDowell 2017 vs. (LBP) Niederer 2020 | 0.0 | Slight |
| (FM) McDowell 2017 vs. (LBP) Quentin 2021 | 0.0 | Slight |
| (FM) McDowell 2017 vs. (LBP) Ram 2023 | 0.0 | Slight |
| (FM) McDowell 2017 vs. (LBP) Sutanto 2022 | 0.0 | Slight |
| (FM) McDowell 2017 vs. (LBP) Wewege 2018 | 0.0 | Slight |
| (FM) McDowell 2017 vs. (LBP) Zhang 2019 | 0.0 | Slight |
| (FM) McDowell 2017 vs. (LBP) Zhang 2022 | 0.0 | Slight |
| (FM) McDowell 2017 vs. (NP) Wilhelm 2020 | 0.0 | Slight |
| (FM) McDowell 2017 vs. (NP) Yang 2022 | 0.0 | Slight |
| (FM) McDowell 2017 vs. (RA) Baillet 2010 | 0.0 | Slight |
| (FM) McDowell 2017 vs. (RA) Baillet 2012 | 0.0 | Slight |
| (FM) McDowell 2017 vs. (SP) Larsson 2019 | 0.0 | Slight |
| (KP) Hu 2021 vs. (KP) Jansen 2011 | 0.0 | Slight |
| (KP) Hu 2021 vs. (KP) Pedersen 2022 | 0.0 | Slight |
| (KP) Hu 2021 vs. (LBP) Aladro-Gonzalvo 2013 | 0.0 | Slight |
| (KP) Hu 2021 vs. (LBP) Anheyer 2021 | 0.0 | Slight |
| (KP) Hu 2021 vs. (LBP) Gao 2022 | 0.0 | Slight |
| (KP) Hu 2021 vs. (LBP) Mueller 2020 | 0.0 | Slight |
| (KP) Hu 2021 vs. (LBP) Niederer 2020 | 0.0 | Slight |
| (KP) Hu 2021 vs. (LBP) Quentin 2021 | 0.0 | Slight |
| (KP) Hu 2021 vs. (LBP) Ram 2023 | 0.0 | Slight |
| (KP) Hu 2021 vs. (LBP) Sutanto 2022 | 0.0 | Slight |
| (KP) Hu 2021 vs. (LBP) Wewege 2018 | 0.0 | Slight |
| (KP) Hu 2021 vs. (LBP) Zhang 2019 | 0.0 | Slight |
| (KP) Hu 2021 vs. (LBP) Zhang 2022 | 0.0 | Slight |
| (KP) Hu 2021 vs. (NP) Wilhelm 2020 | 0.0 | Slight |
| (KP) Hu 2021 vs. (NP) Yang 2022 | 0.0 | Slight |
| (KP) Hu 2021 vs. (RA) Baillet 2010 | 0.0 | Slight |
| (KP) Hu 2021 vs. (RA) Baillet 2012 | 0.0 | Slight |
| (KP) Hu 2021 vs. (SP) Larsson 2019 | 0.0 | Slight |
| (KP) Imoto 2019 vs. (LBP) Aladro-Gonzalvo 2013 | 0.0 | Slight |
| (KP) Imoto 2019 vs. (LBP) Anheyer 2021 | 0.0 | Slight |
| (KP) Imoto 2019 vs. (LBP) Gao 2022 | 0.0 | Slight |
| (KP) Imoto 2019 vs. (LBP) Mueller 2020 | 0.0 | Slight |
| (KP) Imoto 2019 vs. (LBP) Niederer 2020 | 0.0 | Slight |
| (KP) Imoto 2019 vs. (LBP) Quentin 2021 | 0.0 | Slight |
| (KP) Imoto 2019 vs. (LBP) Ram 2023 | 0.0 | Slight |
| (KP) Imoto 2019 vs. (LBP) Sutanto 2022 | 0.0 | Slight |
| (KP) Imoto 2019 vs. (LBP) Wewege 2018 | 0.0 | Slight |
| (KP) Imoto 2019 vs. (LBP) Zhang 2019 | 0.0 | Slight |
| (KP) Imoto 2019 vs. (LBP) Zhang 2022 | 0.0 | Slight |
| (KP) Imoto 2019 vs. (NP) Wilhelm 2020 | 0.0 | Slight |
| (KP) Imoto 2019 vs. (NP) Yang 2022 | 0.0 | Slight |
| (KP) Imoto 2019 vs. (RA) Baillet 2010 | 0.0 | Slight |
| (KP) Imoto 2019 vs. (RA) Baillet 2012 | 0.0 | Slight |
| (KP) Imoto 2019 vs. (SP) Larsson 2019 | 0.0 | Slight |
| (KP) Jansen 2011 vs. (KP) Li 2020 | 0.0 | Slight |
| (KP) Jansen 2011 vs. (LBP) Aladro-Gonzalvo 2013 | 0.0 | Slight |
| (KP) Jansen 2011 vs. (LBP) Anheyer 2021 | 0.0 | Slight |
| (KP) Jansen 2011 vs. (LBP) Gao 2022 | 0.0 | Slight |
| (KP) Jansen 2011 vs. (LBP) Mueller 2020 | 0.0 | Slight |
| (KP) Jansen 2011 vs. (LBP) Niederer 2020 | 0.0 | Slight |
| (KP) Jansen 2011 vs. (LBP) Quentin 2021 | 0.0 | Slight |
| (KP) Jansen 2011 vs. (LBP) Ram 2023 | 0.0 | Slight |
| (KP) Jansen 2011 vs. (LBP) Sutanto 2022 | 0.0 | Slight |
| (KP) Jansen 2011 vs. (LBP) Wewege 2018 | 0.0 | Slight |
| (KP) Jansen 2011 vs. (LBP) Zhang 2019 | 0.0 | Slight |
| (KP) Jansen 2011 vs. (LBP) Zhang 2022 | 0.0 | Slight |
| (KP) Jansen 2011 vs. (NP) Wilhelm 2020 | 0.0 | Slight |
| (KP) Jansen 2011 vs. (NP) Yang 2022 | 0.0 | Slight |
| (KP) Jansen 2011 vs. (RA) Baillet 2010 | 0.0 | Slight |
| (KP) Jansen 2011 vs. (RA) Baillet 2012 | 0.0 | Slight |
| (KP) Jansen 2011 vs. (SP) Larsson 2019 | 0.0 | Slight |
| (KP) Juhl 2014 vs. (LBP) Aladro-Gonzalvo 2013 | 0.0 | Slight |
| (KP) Juhl 2014 vs. (LBP) Anheyer 2021 | 0.0 | Slight |
| (KP) Juhl 2014 vs. (LBP) Gao 2022 | 0.0 | Slight |
| (KP) Juhl 2014 vs. (LBP) Mueller 2020 | 0.0 | Slight |
| (KP) Juhl 2014 vs. (LBP) Niederer 2020 | 0.0 | Slight |
| (KP) Juhl 2014 vs. (LBP) Quentin 2021 | 0.0 | Slight |
| (KP) Juhl 2014 vs. (LBP) Ram 2023 | 0.0 | Slight |
| (KP) Juhl 2014 vs. (LBP) Sutanto 2022 | 0.0 | Slight |
| (KP) Juhl 2014 vs. (LBP) Wewege 2018 | 0.0 | Slight |
| (KP) Juhl 2014 vs. (LBP) Zhang 2019 | 0.0 | Slight |
| (KP) Juhl 2014 vs. (LBP) Zhang 2022 | 0.0 | Slight |
| (KP) Juhl 2014 vs. (NP) Wilhelm 2020 | 0.0 | Slight |
| (KP) Juhl 2014 vs. (NP) Yang 2022 | 0.0 | Slight |
| (KP) Juhl 2014 vs. (RA) Baillet 2010 | 0.0 | Slight |
| (KP) Juhl 2014 vs. (RA) Baillet 2012 | 0.0 | Slight |
| (KP) Juhl 2014 vs. (SP) Larsson 2019 | 0.0 | Slight |
| (KP) Li 2016 vs. (KP) Li 2020 | 0.0 | Slight |
| (KP) Li 2016 vs. (LBP) Aladro-Gonzalvo 2013 | 0.0 | Slight |
| (KP) Li 2016 vs. (LBP) Anheyer 2021 | 0.0 | Slight |
| (KP) Li 2016 vs. (LBP) Gao 2022 | 0.0 | Slight |
| (KP) Li 2016 vs. (LBP) Mueller 2020 | 0.0 | Slight |
| (KP) Li 2016 vs. (LBP) Niederer 2020 | 0.0 | Slight |
| (KP) Li 2016 vs. (LBP) Quentin 2021 | 0.0 | Slight |
| (KP) Li 2016 vs. (LBP) Ram 2023 | 0.0 | Slight |
| (KP) Li 2016 vs. (LBP) Sutanto 2022 | 0.0 | Slight |
| (KP) Li 2016 vs. (LBP) Wewege 2018 | 0.0 | Slight |
| (KP) Li 2016 vs. (LBP) Zhang 2019 | 0.0 | Slight |
| (KP) Li 2016 vs. (LBP) Zhang 2022 | 0.0 | Slight |
| (KP) Li 2016 vs. (NP) Wilhelm 2020 | 0.0 | Slight |
| (KP) Li 2016 vs. (NP) Yang 2022 | 0.0 | Slight |
| (KP) Li 2016 vs. (RA) Baillet 2010 | 0.0 | Slight |
| (KP) Li 2016 vs. (RA) Baillet 2012 | 0.0 | Slight |
| (KP) Li 2016 vs. (SP) Larsson 2019 | 0.0 | Slight |
| (KP) Li 2020 vs. (KP) Pedersen 2022 | 0.0 | Slight |
| (KP) Li 2020 vs. (LBP) Aladro-Gonzalvo 2013 | 0.0 | Slight |
| (KP) Li 2020 vs. (LBP) Anheyer 2021 | 0.0 | Slight |
| (KP) Li 2020 vs. (LBP) Gao 2022 | 0.0 | Slight |
| (KP) Li 2020 vs. (LBP) Mueller 2020 | 0.0 | Slight |
| (KP) Li 2020 vs. (LBP) Niederer 2020 | 0.0 | Slight |
| (KP) Li 2020 vs. (LBP) Quentin 2021 | 0.0 | Slight |
| (KP) Li 2020 vs. (LBP) Ram 2023 | 0.0 | Slight |
| (KP) Li 2020 vs. (LBP) Sutanto 2022 | 0.0 | Slight |
| (KP) Li 2020 vs. (LBP) Wewege 2018 | 0.0 | Slight |
| (KP) Li 2020 vs. (LBP) Zhang 2019 | 0.0 | Slight |
| (KP) Li 2020 vs. (LBP) Zhang 2022 | 0.0 | Slight |
| (KP) Li 2020 vs. (NP) Wilhelm 2020 | 0.0 | Slight |
| (KP) Li 2020 vs. (NP) Yang 2022 | 0.0 | Slight |
| (KP) Li 2020 vs. (RA) Baillet 2010 | 0.0 | Slight |
| (KP) Li 2020 vs. (RA) Baillet 2012 | 0.0 | Slight |
| (KP) Li 2020 vs. (SP) Larsson 2019 | 0.0 | Slight |
| (KP) Pedersen 2022 vs. (LBP) Aladro-Gonzalvo 2013 | 0.0 | Slight |
| (KP) Pedersen 2022 vs. (LBP) Anheyer 2021 | 0.0 | Slight |
| (KP) Pedersen 2022 vs. (LBP) Gao 2022 | 0.0 | Slight |
| (KP) Pedersen 2022 vs. (LBP) Mueller 2020 | 0.0 | Slight |
| (KP) Pedersen 2022 vs. (LBP) Niederer 2020 | 0.0 | Slight |
| (KP) Pedersen 2022 vs. (LBP) Quentin 2021 | 0.0 | Slight |
| (KP) Pedersen 2022 vs. (LBP) Ram 2023 | 0.0 | Slight |
| (KP) Pedersen 2022 vs. (LBP) Sutanto 2022 | 0.0 | Slight |
| (KP) Pedersen 2022 vs. (LBP) Wewege 2018 | 0.0 | Slight |
| (KP) Pedersen 2022 vs. (LBP) Zhang 2019 | 0.0 | Slight |
| (KP) Pedersen 2022 vs. (LBP) Zhang 2022 | 0.0 | Slight |
| (KP) Pedersen 2022 vs. (NP) Wilhelm 2020 | 0.0 | Slight |
| (KP) Pedersen 2022 vs. (NP) Yang 2022 | 0.0 | Slight |
| (KP) Pedersen 2022 vs. (RA) Baillet 2010 | 0.0 | Slight |
| (KP) Pedersen 2022 vs. (RA) Baillet 2012 | 0.0 | Slight |
| (KP) Pedersen 2022 vs. (SP) Larsson 2019 | 0.0 | Slight |
| (KP) Tanaka 2014 vs. (LBP) Aladro-Gonzalvo 2013 | 0.0 | Slight |
| (KP) Tanaka 2014 vs. (LBP) Anheyer 2021 | 0.0 | Slight |
| (KP) Tanaka 2014 vs. (LBP) Gao 2022 | 0.0 | Slight |
| (KP) Tanaka 2014 vs. (LBP) Mueller 2020 | 0.0 | Slight |
| (KP) Tanaka 2014 vs. (LBP) Niederer 2020 | 0.0 | Slight |
| (KP) Tanaka 2014 vs. (LBP) Quentin 2021 | 0.0 | Slight |
| (KP) Tanaka 2014 vs. (LBP) Ram 2023 | 0.0 | Slight |
| (KP) Tanaka 2014 vs. (LBP) Sutanto 2022 | 0.0 | Slight |
| (KP) Tanaka 2014 vs. (LBP) Wewege 2018 | 0.0 | Slight |
| (KP) Tanaka 2014 vs. (LBP) Zhang 2019 | 0.0 | Slight |
| (KP) Tanaka 2014 vs. (LBP) Zhang 2022 | 0.0 | Slight |
| (KP) Tanaka 2014 vs. (NP) Wilhelm 2020 | 0.0 | Slight |
| (KP) Tanaka 2014 vs. (NP) Yang 2022 | 0.0 | Slight |
| (KP) Tanaka 2014 vs. (RA) Baillet 2010 | 0.0 | Slight |
| (KP) Tanaka 2014 vs. (RA) Baillet 2012 | 0.0 | Slight |
| (KP) Tanaka 2014 vs. (SP) Larsson 2019 | 0.0 | Slight |
| (LBP) Aladro-Gonzalvo 2013 vs. (LBP) Anheyer 2021 | 0.0 | Slight |
| (LBP) Aladro-Gonzalvo 2013 vs. (LBP) Gao 2022 | 0.0 | Slight |
| (LBP) Aladro-Gonzalvo 2013 vs. (LBP) Mueller 2020 | 0.0 | Slight |
| (LBP) Aladro-Gonzalvo 2013 vs. (LBP) Niederer 2020 | 0.0 | Slight |
| (LBP) Aladro-Gonzalvo 2013 vs. (LBP) Quentin 2021 | 0.0 | Slight |
| (LBP) Aladro-Gonzalvo 2013 vs. (LBP) Ram 2023 | 0.0 | Slight |
| (LBP) Aladro-Gonzalvo 2013 vs. (LBP) Sutanto 2022 | 0.0 | Slight |
| (LBP) Aladro-Gonzalvo 2013 vs. (LBP) Wewege 2018 | 0.0 | Slight |
| (LBP) Aladro-Gonzalvo 2013 vs. (LBP) Zhang 2019 | 0.0 | Slight |
| (LBP) Aladro-Gonzalvo 2013 vs. (LBP) Zhang 2022 | 0.0 | Slight |
| (LBP) Aladro-Gonzalvo 2013 vs. (NP) Wilhelm 2020 | 0.0 | Slight |
| (LBP) Aladro-Gonzalvo 2013 vs. (NP) Yang 2022 | 0.0 | Slight |
| (LBP) Aladro-Gonzalvo 2013 vs. (RA) Baillet 2010 | 0.0 | Slight |
| (LBP) Aladro-Gonzalvo 2013 vs. (RA) Baillet 2012 | 0.0 | Slight |
| (LBP) Aladro-Gonzalvo 2013 vs. (SP) Larsson 2019 | 0.0 | Slight |
| (LBP) Anheyer 2021 vs. (LBP) Gao 2022 | 0.0 | Slight |
| (LBP) Anheyer 2021 vs. (LBP) Niederer 2020 | 0.0 | Slight |
| (LBP) Anheyer 2021 vs. (LBP) Ram 2023 | 0.0 | Slight |
| (LBP) Anheyer 2021 vs. (LBP) Sutanto 2022 | 0.0 | Slight |
| (LBP) Anheyer 2021 vs. (LBP) Wewege 2018 | 0.0 | Slight |
| (LBP) Anheyer 2021 vs. (NP) Wilhelm 2020 | 0.0 | Slight |
| (LBP) Anheyer 2021 vs. (NP) Yang 2022 | 0.0 | Slight |
| (LBP) Anheyer 2021 vs. (RA) Baillet 2010 | 0.0 | Slight |
| (LBP) Anheyer 2021 vs. (RA) Baillet 2012 | 0.0 | Slight |
| (LBP) Anheyer 2021 vs. (SP) Larsson 2019 | 0.0 | Slight |
| (LBP) Gao 2022 vs. (LBP) Niederer 2020 | 0.0 | Slight |
| (LBP) Gao 2022 vs. (LBP) Quentin 2021 | 0.0 | Slight |
| (LBP) Gao 2022 vs. (LBP) Ram 2023 | 0.0 | Slight |
| (LBP) Gao 2022 vs. (LBP) Sutanto 2022 | 0.0 | Slight |
| (LBP) Gao 2022 vs. (LBP) Wewege 2018 | 0.0 | Slight |
| (LBP) Gao 2022 vs. (LBP) Zhang 2019 | 0.0 | Slight |
| (LBP) Gao 2022 vs. (NP) Wilhelm 2020 | 0.0 | Slight |
| (LBP) Gao 2022 vs. (NP) Yang 2022 | 0.0 | Slight |
| (LBP) Gao 2022 vs. (RA) Baillet 2010 | 0.0 | Slight |
| (LBP) Gao 2022 vs. (RA) Baillet 2012 | 0.0 | Slight |
| (LBP) Gao 2022 vs. (SP) Larsson 2019 | 0.0 | Slight |
| (LBP) Mueller 2020 vs. (LBP) Ram 2023 | 0.0 | Slight |
| (LBP) Mueller 2020 vs. (LBP) Wewege 2018 | 0.0 | Slight |
| (LBP) Mueller 2020 vs. (NP) Wilhelm 2020 | 0.0 | Slight |
| (LBP) Mueller 2020 vs. (NP) Yang 2022 | 0.0 | Slight |
| (LBP) Mueller 2020 vs. (RA) Baillet 2010 | 0.0 | Slight |
| (LBP) Mueller 2020 vs. (RA) Baillet 2012 | 0.0 | Slight |
| (LBP) Mueller 2020 vs. (SP) Larsson 2019 | 0.0 | Slight |
| (LBP) Niederer 2020 vs. (LBP) Quentin 2021 | 0.0 | Slight |
| (LBP) Niederer 2020 vs. (LBP) Ram 2023 | 0.0 | Slight |
| (LBP) Niederer 2020 vs. (LBP) Wewege 2018 | 0.0 | Slight |
| (LBP) Niederer 2020 vs. (LBP) Zhang 2019 | 0.0 | Slight |
| (LBP) Niederer 2020 vs. (LBP) Zhang 2022 | 0.0 | Slight |
| (LBP) Niederer 2020 vs. (NP) Wilhelm 2020 | 0.0 | Slight |
| (LBP) Niederer 2020 vs. (NP) Yang 2022 | 0.0 | Slight |
| (LBP) Niederer 2020 vs. (RA) Baillet 2010 | 0.0 | Slight |
| (LBP) Niederer 2020 vs. (RA) Baillet 2012 | 0.0 | Slight |
| (LBP) Niederer 2020 vs. (SP) Larsson 2019 | 0.0 | Slight |
| (LBP) Quentin 2021 vs. (LBP) Ram 2023 | 0.0 | Slight |
| (LBP) Quentin 2021 vs. (LBP) Sutanto 2022 | 0.0 | Slight |
| (LBP) Quentin 2021 vs. (LBP) Wewege 2018 | 0.0 | Slight |
| (LBP) Quentin 2021 vs. (LBP) Zhang 2019 | 0.0 | Slight |
| (LBP) Quentin 2021 vs. (LBP) Zhang 2022 | 0.0 | Slight |
| (LBP) Quentin 2021 vs. (NP) Wilhelm 2020 | 0.0 | Slight |
| (LBP) Quentin 2021 vs. (NP) Yang 2022 | 0.0 | Slight |
| (LBP) Quentin 2021 vs. (RA) Baillet 2010 | 0.0 | Slight |
| (LBP) Quentin 2021 vs. (RA) Baillet 2012 | 0.0 | Slight |
| (LBP) Quentin 2021 vs. (SP) Larsson 2019 | 0.0 | Slight |
| (LBP) Ram 2023 vs. (LBP) Sutanto 2022 | 0.0 | Slight |
| (LBP) Ram 2023 vs. (LBP) Zhang 2019 | 0.0 | Slight |
| (LBP) Ram 2023 vs. (LBP) Zhang 2022 | 0.0 | Slight |
| (LBP) Ram 2023 vs. (NP) Wilhelm 2020 | 0.0 | Slight |
| (LBP) Ram 2023 vs. (NP) Yang 2022 | 0.0 | Slight |
| (LBP) Ram 2023 vs. (RA) Baillet 2010 | 0.0 | Slight |
| (LBP) Ram 2023 vs. (RA) Baillet 2012 | 0.0 | Slight |
| (LBP) Ram 2023 vs. (SP) Larsson 2019 | 0.0 | Slight |
| (LBP) Sutanto 2022 vs. (LBP) Wewege 2018 | 0.0 | Slight |
| (LBP) Sutanto 2022 vs. (LBP) Zhang 2019 | 0.0 | Slight |
| (LBP) Sutanto 2022 vs. (NP) Wilhelm 2020 | 0.0 | Slight |
| (LBP) Sutanto 2022 vs. (NP) Yang 2022 | 0.0 | Slight |
| (LBP) Sutanto 2022 vs. (RA) Baillet 2010 | 0.0 | Slight |
| (LBP) Sutanto 2022 vs. (RA) Baillet 2012 | 0.0 | Slight |
| (LBP) Sutanto 2022 vs. (SP) Larsson 2019 | 0.0 | Slight |
| (LBP) Wewege 2018 vs. (LBP) Zhang 2019 | 0.0 | Slight |
| (LBP) Wewege 2018 vs. (LBP) Zhang 2022 | 0.0 | Slight |
| (LBP) Wewege 2018 vs. (NP) Wilhelm 2020 | 0.0 | Slight |
| (LBP) Wewege 2018 vs. (NP) Yang 2022 | 0.0 | Slight |
| (LBP) Wewege 2018 vs. (RA) Baillet 2010 | 0.0 | Slight |
| (LBP) Wewege 2018 vs. (RA) Baillet 2012 | 0.0 | Slight |
| (LBP) Wewege 2018 vs. (SP) Larsson 2019 | 0.0 | Slight |
| (LBP) Zhang 2019 vs. (NP) Wilhelm 2020 | 0.0 | Slight |
| (LBP) Zhang 2019 vs. (NP) Yang 2022 | 0.0 | Slight |
| (LBP) Zhang 2019 vs. (RA) Baillet 2010 | 0.0 | Slight |
| (LBP) Zhang 2019 vs. (RA) Baillet 2012 | 0.0 | Slight |
| (LBP) Zhang 2019 vs. (SP) Larsson 2019 | 0.0 | Slight |
| (LBP) Zhang 2022 vs. (NP) Wilhelm 2020 | 0.0 | Slight |
| (LBP) Zhang 2022 vs. (NP) Yang 2022 | 0.0 | Slight |
| (LBP) Zhang 2022 vs. (RA) Baillet 2010 | 0.0 | Slight |
| (LBP) Zhang 2022 vs. (RA) Baillet 2012 | 0.0 | Slight |
| (LBP) Zhang 2022 vs. (SP) Larsson 2019 | 0.0 | Slight |
| (NP) Wilhelm 2020 vs. (RA) Baillet 2010 | 0.0 | Slight |
| (NP) Wilhelm 2020 vs. (RA) Baillet 2012 | 0.0 | Slight |
| (NP) Wilhelm 2020 vs. (SP) Larsson 2019 | 0.0 | Slight |
| (NP) Yang 2022 vs. (RA) Baillet 2010 | 0.0 | Slight |
| (NP) Yang 2022 vs. (RA) Baillet 2012 | 0.0 | Slight |
| (NP) Yang 2022 vs. (SP) Larsson 2019 | 0.0 | Slight |
| (RA) Baillet 2010 vs. (SP) Larsson 2019 | 0.0 | Slight |
| (RA) Baillet 2012 vs. (SP) Larsson 2019 | 0.0 | Slight |
| Overall | 1.3 | Slight |

Abbreviations: RA, rheumatoid arthritis; SP, shoulder pain; NP, neck pain; LBP, low back pain; KP, knee pain; FM, fibromyalgia; Com, combination; AT, Achilles tendinopathy; CCA, corrected covered area.
